# Digital Health Platform for Improving the Effect of the Active Health Management of Chronic Diseases in the Community: Mixed Methods Exploratory Study

**DOI:** 10.2196/50959

**Published:** 2024-11-18

**Authors:** Zhiheng Zhou, Danian Jin, Jinghua He, Shengqing Zhou, Jiang Wu, Shuangxi Wang, Yang Zhang, Tianyuan Feng

**Affiliations:** 1 Pingshan Hospital of Southern Medical University Shenzhen China; 2 University of Washington Seattle, WA United States

**Keywords:** information platform, active health, chronic disease management, effectiveness, community, digital health, health literacy, cardio-cerebrovascular disease, China

## Abstract

**Background:**

China is vigorously promoting the health management of chronic diseases and exploring digital active health management. However, as most medical information systems in China have been built separately, there is poor sharing of medical information. It is difficult to achieve interconnectivity among community residents’ self-testing information, community health care information, and hospital health information, and digital chronic disease management has not been widely applied in China.

**Objective:**

This study aimed to build a digital health platform and improve the effectiveness of full-cycle management for community chronic diseases through digital active health management.

**Methods:**

This was a single-arm pre-post intervention study involving the development and use of a digital health platform (2-year intervention; 2020 to 2022). The digital health platform included the “i Active Health” applet for residents and the active health information system (cardio-cerebrovascular disease risk management system) for medical teams. The digital active health management of chronic diseases involved creating health streets, providing internet-assisted full-cycle active health services for residents, implementing internet-based community management for hypertension and diabetes, and performing real-time quantitative assessment and hierarchical management of residents’ risks of cardio-cerebrovascular disease. After the 2-year intervention, management effectiveness was evaluated.

**Results:**

We constructed a digital health platform with interconnected health information and implemented a digital active health management model. After the intervention, the 2-way referral between community health care institutions and hospitals increased. Residents’ health literacy rate increased from 30.6% (3062/10,000) in 2020 to 49.9% (4992/10,000) in 2022, with improvements in health knowledge, health behavior, and health skills. Moreover, the risk of cardio-cerebrovascular disease decreased after the intervention. The community hypertension and diabetes standardized management rates increased from 59.6% (2124/3566) and 55.8% (670/1200) in 2020 to 75.0% (3212/4285) and 69.4% (1686/2430) in 2022, respectively. The control rates of blood pressure in patients with hypertension and blood sugar in patients with diabetes increased from 51.7% (1081/2091) and 42.0% (373/888) in 2020 to 81.2% (1698/2091) and 73.0% (648/888) in 2022, respectively. The intervention improved patients’ BMI, waist circumference, blood uric acid levels, and low-density lipoprotein cholesterol levels. The drug compliance rate of patients with hypertension and diabetes increased from 33.6% (703/2091) and 36.0% (320/888) in 2020 to 73.3% (1532/2091) and 75.8% (673/888) in 2022, respectively. The intervention greatly improved the diet behavior, exercise behavior, and drinking behavior of patients with hypertension and diabetes.

**Conclusions:**

Our digital health platform can effectively achieve the interconnection and exchange of different health information. The digital active health management carried out with the assistance of this platform improved the effectiveness of community chronic disease management. Thus, the platform is worth promoting and applying in practice.

## Introduction

Since the promulgation of “Healthy China Action (2019-2030)” by the Chinese government, China has actively promoted a comprehensive and full life cycle health strategy, taking “popularizing knowledge and improving literacy, self-discipline, healthy living, early intervention, services, mass participation, joint construction, and sharing” as the implementation principle of Healthy China [1,2]. The concept of “active health” was first proposed by Chinese scholars in 2015 [3,4]. Active health is a novel medical model that involves actively applying controllable stimuli to the human body, increasing its micro complexity, promoting diverse adaptation, and achieving enhanced human function or reversal of chronic diseases. It emphasizes the long-term continuous dynamic tracking of the entire life cycle behavior system of individuals, and identifying and evaluating their own state, evolutionary direction, and degree, with a focus on selecting various elements of lifestyle, fully exerting their subjective initiative, and improving health behavior. By comprehensively using various medical methods, controllable and active interventions are carried out on human behavior, promoting self-organized adaptive changes in the human body and thereby achieving functional improvement in practical activities and knowledge systems to eliminate diseases and maintain a healthy state of the human body [5,6]. Internet technology and artificial intelligence technology are indispensable tools in achieving active detection and intervention among residents. Although international scholars did not directly put forward the word “active health,” they carried out a lot of related research on active health, including digital health, internet-based medicine, artificial intelligence–assisted health care, and new digital management models, to promote patients’ health knowledge, improve residents’ health behaviors, and improve self-health management [7-9]. It could be predicted that internet-based active health management would be an important way to promote active health.

Internet-based health management is a new medical service model, which is based on the internet and is supported by cloud computing, big data, the Internet of Things, and other information technologies through cross-penetration, integration, and innovation with traditional medical services. This model has openness, interactivity, convenience, and cross-border characteristics, which could maximize the allocation and use of medical resources, diversify medical methods and pathways, facilitate medical processes and mechanisms, and personalize medical needs and services. The practice has proved that internet-based health management has changed the past model of the hospital serving patients, making it transform into a collaborative medical model including doctor-patient collaboration, general practitioner-specialist collaboration, and preventive medicine-clinical medicine integration. It could guide high-quality medical resources to primary care, realize medical resource sharing, and improve the accessibility of high-quality hospital and community health resources. The construction of an internet-based health service model will profoundly change the traditional way of community chronic disease health management, which would be a new trend and direction of health care development [10,11]. The internet and wearable detection devices automatically upload data, allowing residents to receive medical services at their homes. The application of these information technologies will play important guarantee and support roles in promoting the graded diagnosis and treatment of chronic diseases and improving the level of health management of chronic diseases [12,13].

With the development of China’s social economy and the changes in medical conditions, the disease spectrum and death spectrum of residents have significantly changed. There is a continuous upward trend in both the prevalence and mortality of chronic diseases such as diabetes and hypertension. Chronic diseases have become a public health problem seriously threatening the health and life of Chinese residents. The main goal of China’s health strategy is to carry out effective health management for chronic diseases and reduce the incidence of complications and disease burden [14,15]. At present, the overall level of community hypertension and diabetes health management in China is still unsatisfactory, internet-based health care has been applied to chronic disease management in China, and the initial results have been obtained. However, most medical information systems in China have been built separately, and there is limited information sharing available. Even in the same region, it is difficult to achieve interconnection between patients’ information in community health service institutions and hospital medical information systems. This is a bottleneck that hinders the application and promotion of internet-assisted chronic disease management models in China [16,17]. Therefore, this study aimed to build a digital health platform and improve the effectiveness of chronic disease full-cycle management in the community through the implementation of a digital active health management model.

## Methods

### Overview

This was a single-arm pre-post intervention study that involved digital health platform building and use assessment conducted through a 2-year digital active health management intervention for residents from 2 streets. The intervention effectiveness was evaluated by qualitative and quantitative methods after 2 years. This mixed methods approach included quantitative evaluations of health literacy; changes in blood glucose, blood pressure, and blood lipids; and health behaviors of patients with chronic diseases, as well as qualitative evaluations of feedback from residents and health care staff.

### Digital Health Platform Building

#### Objectives and Requirements of the Digital Health Platform

**Figure 1:**
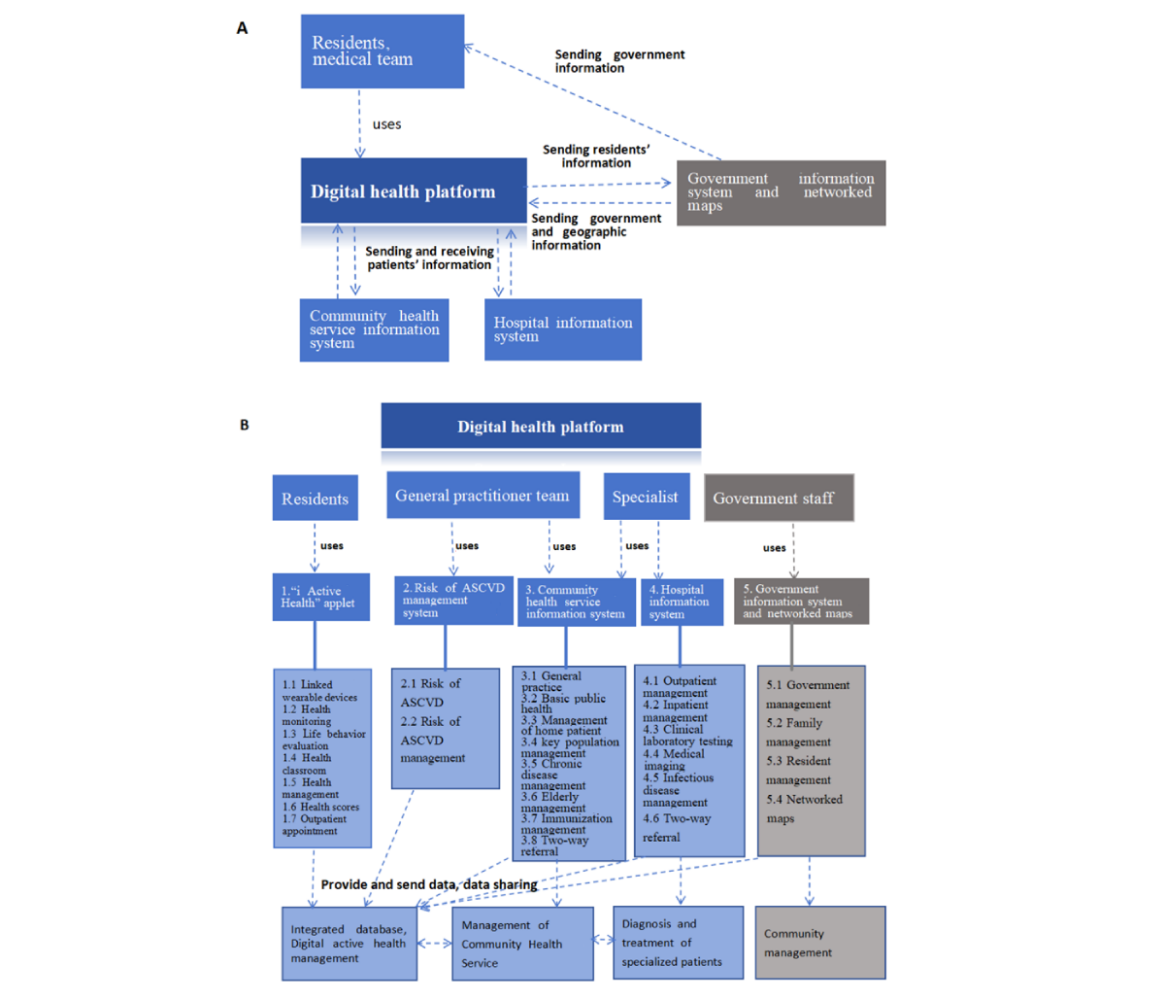
The architecture of the digital health platform. The C4 model and “classification of digital interventions, services, and applications in health” from the World Health Organization were referenced to draw the digital health platform architecture. (A) The system context of the architecture of the digital health platform. (B) The containers and components of the architecture of the digital health platform, which included users, systems (containers), contents (components), and their functions. The blue text boxes represent the systems within the platform, while the gray text boxes represent the external systems. Arrows represent data applications, and the solid line represents the content included. ASCVD: atherosclerotic cardiovascular disease.

The objective of the digital health platform was to realize the interconnection of residents’ wearable devices, applets for residents, community health service center information systems, hospital information systems, and government information systems, and promote residents to actively obtain health knowledge, monitor health and behaviors, and participate in health interventions through the platform. The platform would allow the medical team to comprehensively collect residents’ health information, conduct intelligent health risk assessment and hierarchical management, and create personalized health interventions to finally achieve the goal of accurate health management for patients with hypertension and diabetes in the community. This platform included the “i Active Health” applet, risk of cardio-cerebrovascular disease management system, community health service information system, and hospital information system, which connected with the government information system and network maps. The architecture of the digital health platform is shown in Figure 1.

#### Establishment of the “i Active Health” Applet

**Figure 2:**
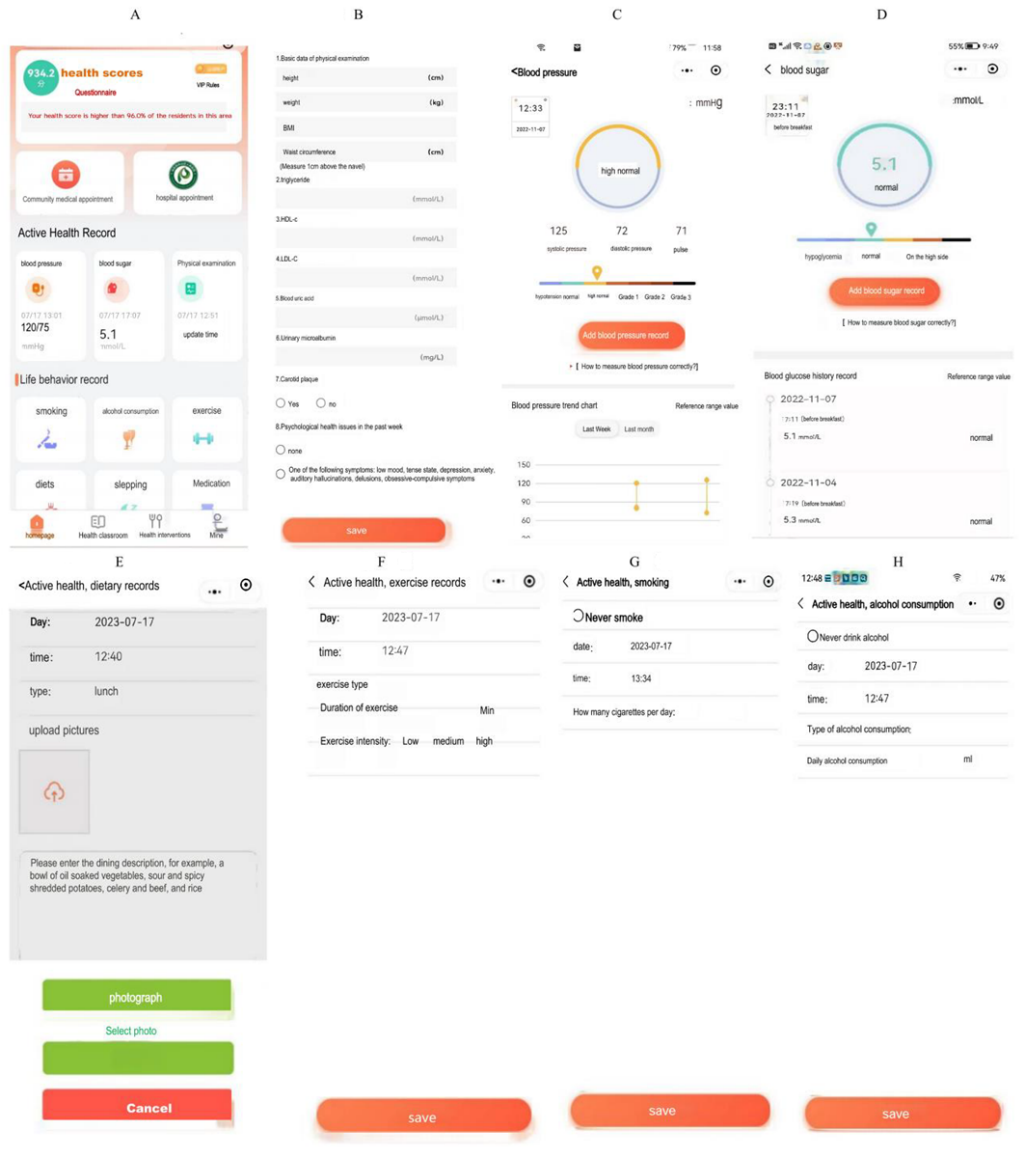
“i Active Health” applet. (A) The homepage of the “i Active Health” applet, which displays health indicators, health scores, and the rank of local residents (top left of the homepage). (B) Health examination results. (C) Self-measured blood pressure results and health management recommendations. (D) Self-test blood glucose results and health management recommendations. (E) Record and analysis of 3 meal recipes. (F) Exercise record and analysis. (G) Smoking record. (H) Record of alcohol consumption.

We established China’s first “i Active Health” applet for residents’ active learning, active health monitoring, and active participation in interventions. The applet linked various wearable devices, such as a sphygmomanometer, glucose meter, health bracelet, sleep monitor, electrocardiogram device, and motion detection equipment. The results could be automatically transferred to the “i Active Health” applet after home testing by the residents, which could promote proactive health testing among residents. There were functions of life behavior evaluation and health prescription formulation. Residents’ exercise and diet information could be evaluated, and targeted dietary prescriptions and exercise prescriptions could be formulated. The risk of cardio-cerebrovascular disease was automatically assessed by allowing residents to enter health information or by capturing residents’ health information from community health information systems and hospital information systems, which could promote residents to actively improve their living behaviors and participate in health management. There was a health classroom with health-related courses (graphics, text, video, and voice) available for residents, which could promote residents to actively obtain health knowledge and skills. There was a health management function. The general practitioner team developed health management plans for patients, provided online guidance, and scheduled follow-up appointments through the “i Active Health” applet, which could encourage patients to actively participate in health management (Figure 2). The “i Active Health” applet registration guidelines are provided in Multimedia Appendix 1. The training materials for using the “i Active Health” applet are presented in Multimedia Appendix 2. The health scores are presented in Multimedia Appendix 3.

#### Establishment of a Risk Management System for Atherosclerotic Cardiovascular Disease

**Figure 3:**
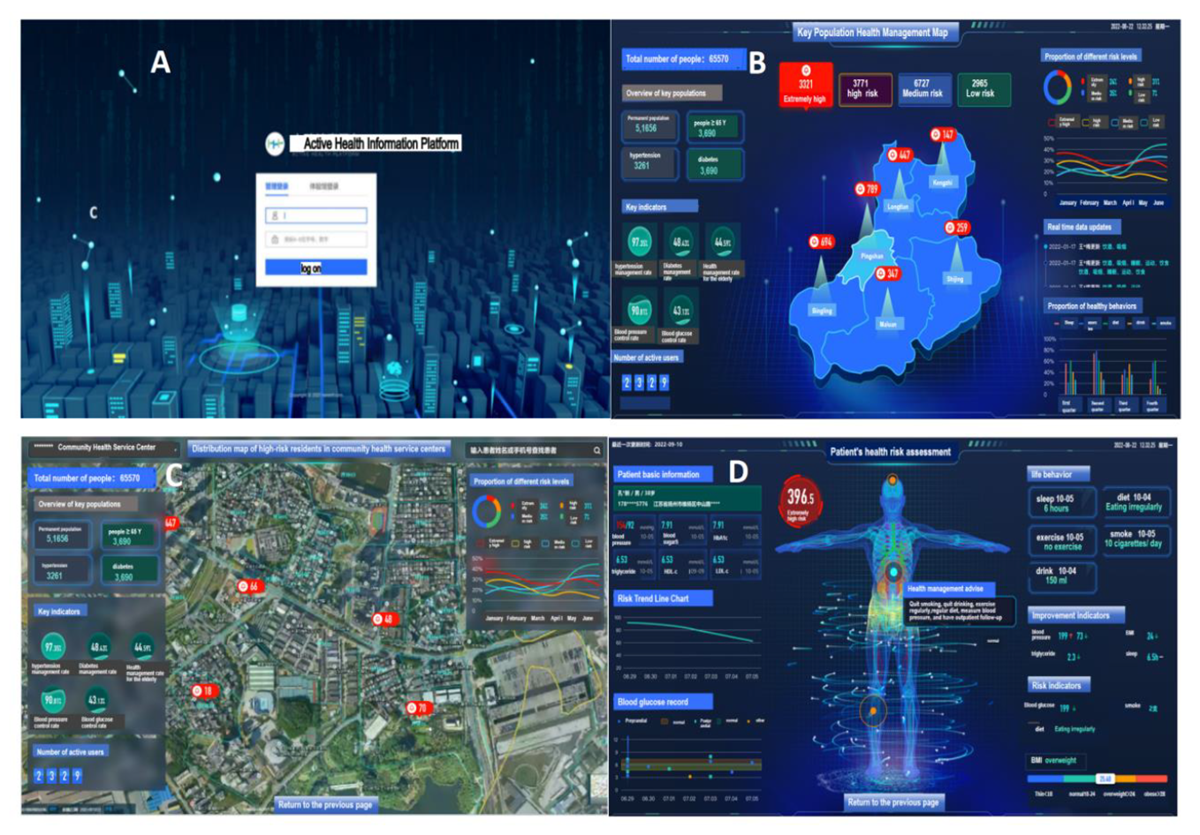
Risk management system of cardio-cerebrovascular disease. (A) The homepage of the active health information platform. (B) The regional risk assessment page shows the risk status of cardio-cerebrovascular disease and the main data of health management of residents in all streets of Pingshan district. (C) The community risk assessment page shows the risk status of cardio-cerebrovascular disease and the main data of health management of residents in some communities. (D) The individual health risk page shows the health indicators and risk scores of cardio-cerebrovascular disease in individual residents.

We screened 24 risk assessment indicators (including basic medical history risk, health behavior risk, and health effect risk) through a series of methods and established a risk assessment model for cardio-cerebrovascular disease (RACCVD). A risk management information system was established, and 4 risk assessment tools, including Stroke Risk Scorecard (SRSC) (China) [18], 10-year atherosclerotic cardiovascular disease (ASCVD) (China) [19], Systematic Coronary Risk Evaluation (SCORE) (Europe) [20], and Framingham scale (FRS) (United States) [21], were configured in the system. The system interconnected the “i Active Health” applet, community health information system, hospital information system, and network map. The risk assessment score of cardio-cerebrovascular disease could be calculated, and the system could automatically group hazard levels based on resident’s risk scores, which could realize early identification of high-risk groups and accurate positioning, early warning, and early diagnosis and treatment (Figure 3).

#### Modification of the Community Health Information System

**Figure 4:**
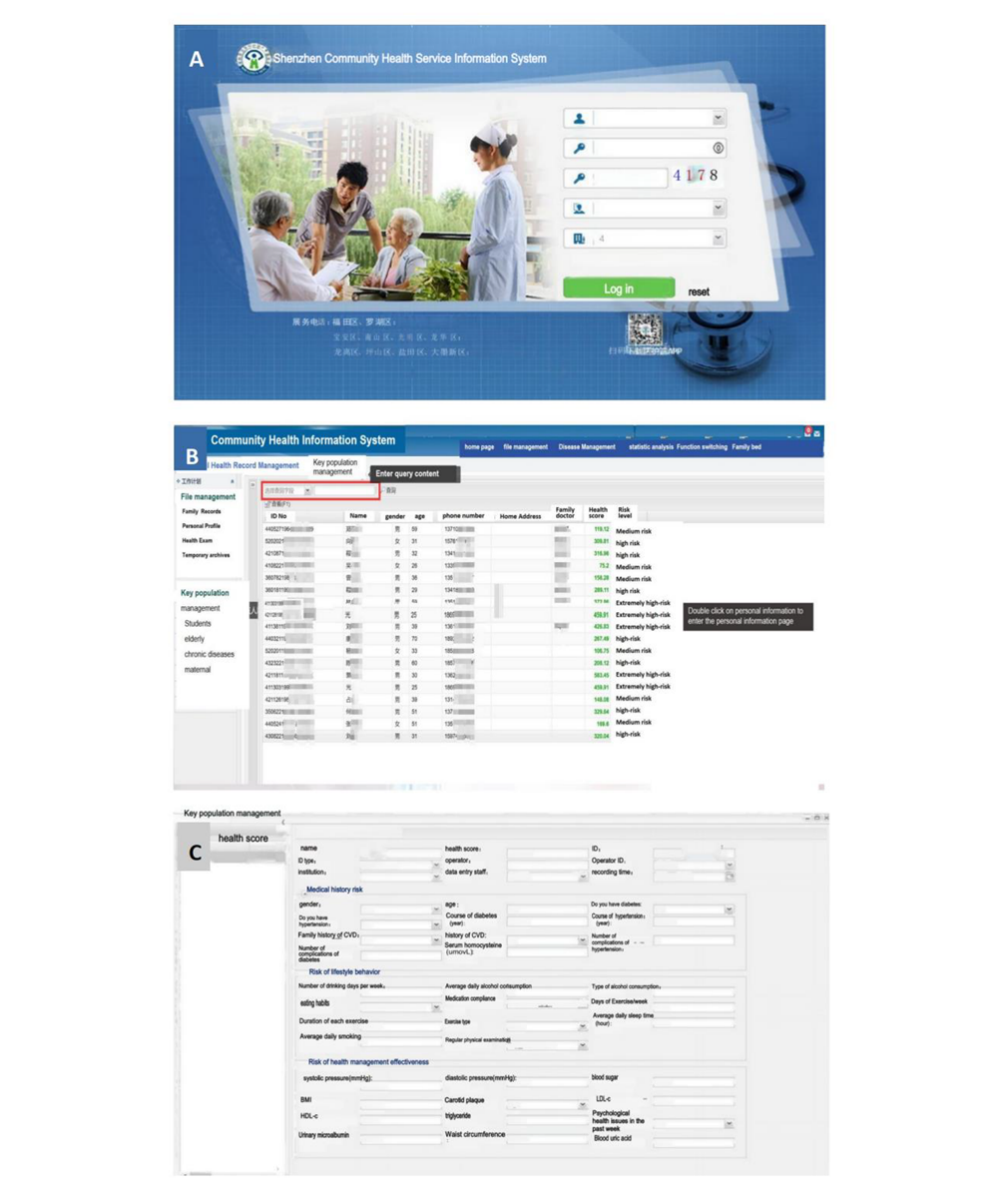
The key population management module in the community health service information system. (A) The homepage of the community health service information system. (B) Residents’ health risk scores and risk levels shown in the key population management module. (C) The risk indicators of cardio-cerebrovascular disease in the key population management module.

The key population management module was added to the community health information system, which was used to interface with the data generated in the “i Active Health” applet and residents’ self-test health data and health records online in a centralized manner, and the “risk score” and “risk level” were shown, based on which family doctors could quickly identify and pay attention to extremely high-risk and high-risk populations, and recommend transfer to higher-level hospitals for diagnosis and treatment (Figure 4).

#### Modification of the Hospital Information System

A 2-way referral module was added to the hospital information system to improve the up and down transfer of patients between the hospital and the community health service center. A remote diagnosis and treatment information system module was added, which promoted hospital experts to participate in remote diagnosis and treatment in community consultations. Therefore, the health data collected by medical institutions and third-party systems (wearable devices) were interconnected, allowing unified collection and comprehensive management.

#### Connection of Government Information Platforms

Patients’ demographic data, personal health behavior, and family status data were connected with the resident management information systems of the government department where they were located to ensure the consistency of resident information in different departments and provide a basis for government departments to carry out health work and ensure the accuracy of patient medical information. At the same time, residents’ information was integrated with the information of the data bureau for precise positioning of patient risk assessment and hierarchical management.

### Platform Application: Digital Active Health Management

#### Selection of Experimental Streets

Biling street and Shijing street in Pingshan District, Shenzhen, China were selected as digital active health management intervention experimental streets.

#### Inclusion Criteria

The inclusion criteria were as follows: (1) diagnosis of hypertension or type 2 diabetes, age ≥65 years, and registration in the community health service center; (2) normal hearing and self-awareness; (3) presence of a smartphone in the family and ability to receive and read online information; (4) ability to complete self-health monitoring and online health classroom learning; and (5) willingness to accept health management and outpatient follow-up provided by general practitioner teams.

#### Exclusion Criteria

The exclusion criteria were as follows: (1) poor hearing and consciousness, and inability to communicate as usual; (2) inability to complete regular self-health tests; and (3) unwillingness to accept health managers or follow-up.

### Digital Active Health Management Intervention Methods

#### Deep Cooperation Between the Government and Health Institutions

The active health service plans for the street have been formulated, which were under the government’s annual key work for promotion. The street office released active health-related information through government information platforms and collaborated with street enterprises, schools, and other parties to build an active health system for residents.

#### Active Health Activities Provided by the Government

The services included establishing teams of health science popularization instructors, regularly providing health training to residents, building health volunteer service teams, cultivating health coaches and health clubs, and inviting community residents to participate in health activities. A total of 50 popular science videos were made. Moreover, a street health culture festival was held; healthy communities, health parks, and health canteens were introduced; and a good health culture atmosphere was created.

#### Active Health Services by Medical Institutions

Digital active health services included conducting health classrooms (video, voice, or text) to promote residents’ active learning on health knowledge and skills, and screening hypertension, diabetes, dyslipidemia, and common tumors among residents. Moreover, the services included health monitoring, health warnings, diet monitoring, and sports health management for residents. Health activities were held on special days, such as world hypertension day, diabetes day, heart disease day, and chronic obstructive pulmonary disease day, and online and offline health consultations and interventions were provided for residents.

#### Optimization of the 2-Way Referral Process

The process of patient referral between community health institutions and hospitals was optimized by a 2-way referral information platform. With regard to transfer from the community health center to the hospital, the hospital prioritized the opening of specialized account sources and focused on priority reception, priority examination, and priority hospitalization for transferred patients. With regard to transfer from the hospital to the community health center, patients who completed diagnosis and treatment in the hospital and met the criteria for referral were referred to the community general practitioner team by specialist doctors through the information system, and joint management of general practitioners and specialists was carried out.

#### ASCVD Risk Assessment and Hierarchical Management

ASCVD risk assessment was conducted for adult residents aged 18 years or older who completed annual health examinations by the “i Active Health” applet and digital health platform. Hierarchical management was carried out according to the risk level of ASCVD. Residents with medium and low risk were managed by community general practitioner teams, while residents with high and extremely high risk were automatically sent to the cardiovascular and neurology departments of the hospital and were managed by specialists.

### Internet-Based Management of Hypertension and Diabetes

#### Chronic Disease Management Team

Each practitioner team included a general practitioner (team leader), nurse, and health manager. At the same time, cardiovascular doctors, endocrinologists, clinical pharmacists, dietitians, and sports rehabilitation specialists were allocated according to the needs of residents to provide technical support for the chronic disease management team.

#### Intervention Process

**Figure 5:**
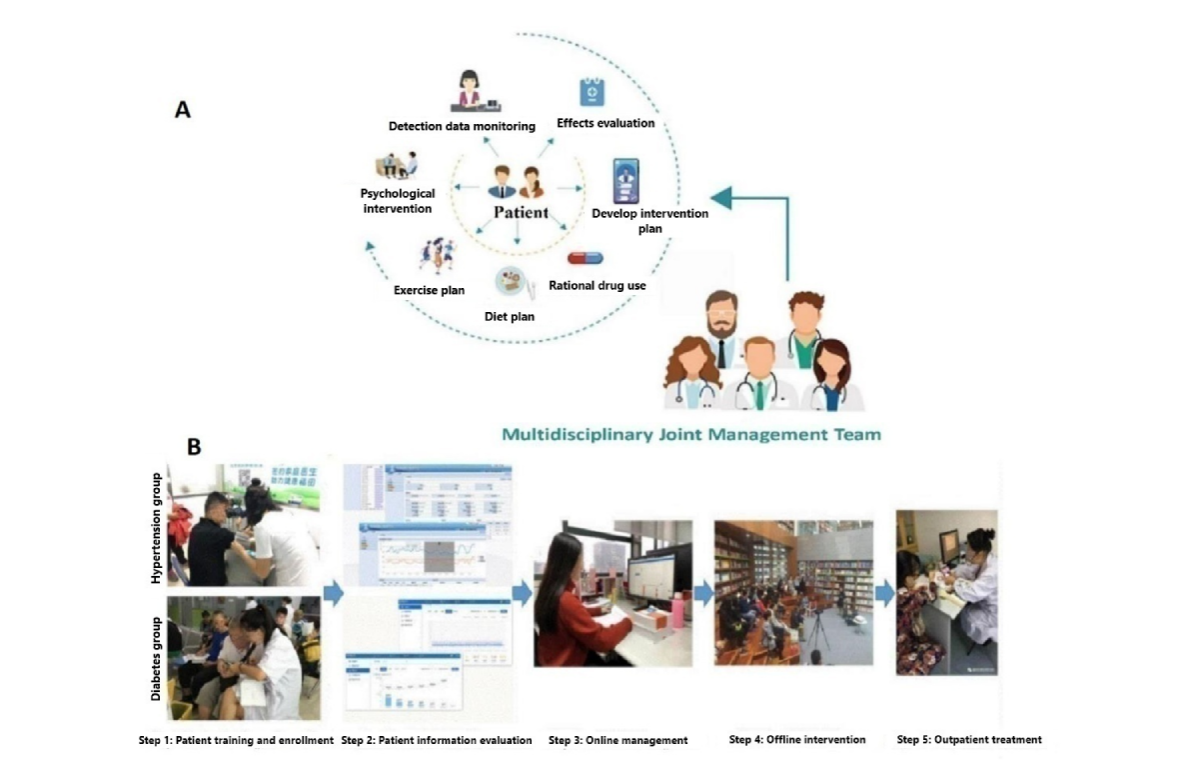
Internet-based management of hypertension and diabetes. (A) Internet-based management team including a general practitioner, a nurse, a health manager, a dietitian, sports rehabilitation specialists, clinical pharmacists, and specialists (cardiovascular doctors and endocrinologists). (B) The 5 steps of internet-based management. It includes patient training and enrollment, patient data collection and evaluation, online monitoring and health management, offline health intervention, and face-to-face outpatient diagnosis and treatment by physicians.

The general practitioner team provided refined management through a combination of online and offline approaches. More than one face-to-face visit was simultaneously conducted per quarter, including condition consultation, physical examination, and clinical diagnosis and treatment. The management process is shown in Figure 5.

The patient self-measured blood pressure or blood sugar through an intelligent sphygmomanometer and glucose meter detection terminal, and the data were automatically transmitted. The active health applet and digital health platform obtained detection data and automatically sent it to the patient’s family members and general practitioner team. The digital health platform grouped and managed patients. The active health applet provided health education courses for patients and their families. The health managers and nurses followed up with patients and their families through WeChat and phone calls, provided health education, and promptly provided feedback on the patient’s condition to the general practitioner. The patient’s family members assisted in urging the patient to take medicine, follow the diet, and perform exercise at home. On-site health education lectures and exchange meetings were regularly held. General practitioners controlled the patient’s condition in real time, provided health management for patients, and referred patients to specialized doctors. Medical experts were invited to conduct joint diagnosis and treatment at community health service centers. The operation manual is presented in Multimedia Appendix 4.

### Effectiveness Evaluation

#### Qualitative Methods

The methods included conducting interviews and on-site supervisions to understand the construction of the digital health platform and the implementation of active health activities among residents and patients in the experimental area.

#### Quantitative Methods

For quantitative assessments, data were collected on residents’ health, questionnaire survey findings, physical examination findings, laboratory testing results, etc, and evaluations were performed of the changes in residents’ health literacy, the ASCVD risk, and the effectiveness of management for hypertension, diabetes, and old age.

### Assessment Tools

#### Health Literacy Questionnaire

The Shenzhen Citizen Health Literacy Questionnaire was applied, which had a total of 32 questions with 5 parts: general condition (5 questions), physical health condition (5 questions), health knowledge (8 questions), daily living habits (8 questions), and health skills (6 questions). The Cronbach α of this questionnaire was .873, Kaiser-Meyer-Olkin (KMO) value was 0.893, and Bartlett test χ^2^_435_ was 4267.20 (*P*<.001).

#### Risk Assessment Model of Cardio-Cerebrovascular Disease

The risk assessment model was developed through a series of methods and validated in the cohort population, which included 24 evaluation indicators for the dimensions of basic medical history risk (age, sex, history of hypertension, history of diabetes, history of cardiovascular and cerebrovascular events, family history of cardiovascular and cerebrovascular diseases, and hyperhomocysteinemia), health behavior risk (smoking, drinking, exercise, diet, sleep, medication, and physical examination), and health effect risk (current blood pressure, current blood sugar, BMI or waist circumference, triglyceride, high-density lipoprotein cholesterol [HDL-C], low-density lipoprotein cholesterol [LDL-C], hyperuricemia, microalbuminuria, carotid plaque, and psychological disorders). The total score was 0-1000, and a higher score indicated a higher risk. According to the risk score, the risk level was divided into low risk, medium risk, high risk, and extremely high risk. The Cronbach α was .873, and the reliability coefficient was 0.891. The indicator system and its calculation method of the risk assessment of cardio-cerebrovascular disease are presented in Multimedia Appendix 5.

#### SRSC (China)

The SRSC was developed by the Committee of Stroke Screening, Prevention and Treatment Project of the National Health Commission of the People’s Republic of China [18]. Overall, “8+2” stroke risk factors were used to assess the risk stratification of patients, among which “8” refers to hypertension, atrial fibrillation or valvular heart disease, diabetes, smoking history, dyslipidemia, lack of exercise, significant overweight or obesity, and a family history of stroke, and “2” refers to a history of stroke or transient ischemic attack. Individuals with 3 or more of the “8” risk factors for stroke or those with a history of transient ischemic attack or stroke were included in the high-risk group. Those who met 3 or fewer factors but had one of the chronic diseases (including hypertension, diabetes, atrial fibrillation, or valvular heart disease) were included in the medium-risk group. Those who met 3 or fewer factors and had no chronic diseases were included in the low-risk group. The scale was reasonably reliable, with a Cronbach α of .77 and a reliability coefficient of 0.803.

#### 10-year ASCVD Risk Assessment Model (China)

The 10-year ASCVD risk assessment model was proposed by the Guidelines for the Prevention and Treatment of Dyslipidemia in Chinese Adults (2016 Revision) [19]. This evaluation model was based on the average incidence risk of diabetes and hypertension, the LDL-C level, the total cholesterol (TC) level, and the number of combined risk factors (men ≥45 years or women ≥55 years, smoking, and low HDL-C). An average incidence risk of <5% was considered low risk, 5%-9% was considered medium risk, and ≥10% was considered high risk. The Cronbach α was .813, and the reliability coefficient was 0.805.

#### SCORE (Europe)

The SCORE is a 10-year risk of fatal cardiovascular disease model in Europe by the SCORE project [20]. The model included 5 risk factors: age, gender, systolic blood pressure, TC, and smoking. An absolute risk of cardiovascular disease onset within 10 years of <1% was considered low risk, ≥1% but <5% was considered medium risk, ≥5% but <10% was considered high risk, and ≥10% was considered extremely high risk. The Cronbach α was .830, and the reliability coefficient was 0.871.

#### FRS (United States)

The FRS was developed and published by researchers from Framingham Heart Research in 1961 [21]. The model included 7 risk factors: gender, age, blood pressure, TC, HDL-C, smoking, and diabetes. An absolute risk of cardiovascular disease onset within 10 years of <5% was considered low risk, ≥5% but <10% was considered medium risk, and ≥10% was considered high risk. The Cronbach α was .701, the split-half reliability coefficient was 0.826, and the test-retest reliability coefficient was 0.94.

#### Medication Compliance Questionnaire

The Medication Compliance Questionnaire (MCQ) was developed by a team led by Professor Thomas Paraidathathu [22,23]. The MCQ included a total of 7 questions. Questions in the MCQ assessed patients’ intentional and unintentional noncompliance to medication instructions, including reasons for noncompliance. A 4-point Likert scale was used for each question: “4,” none of the time; “3,” sometimes (1-4 times per month); “2,” most of the time; and “1,” all the time. A total score of 27 was considered to indicate compliance, which was the evaluation criterion of drug compliance for each patient in this study. The scores may range from 7 to 28. The Cronbach α was .782, and the interrater reliability of 2 interviewers demonstrated a Cohen kappa statistic of 0.796.

### Assessment Methods of Life Behavior

#### Exercise Behavior

Regular exercise means exercising more than 3 times a week, with each continuous exercise session lasting more than 30 minutes.

#### Diet Behavior

Based on the patient’s self-reported information, the dietary status in the past 2 weeks of the survey was determined to be the following: balanced diet, mainly meat and vegetables, mainly vegetarian, salt loving, oil loving, or sugar loving.

#### Smoking

Smoking was defined as smoking one or more cigarettes per day and smoking for 6 months or more.

#### Drinking

According to the “Dietary Guidelines for Chinese Residents (2022),” the definition of alcohol consumption was an average daily alcohol consumption of ≥15 g in the past 2 weeks.

### Diagnosis Standard of Hypertension and Diabetes

#### Hypertension

Essential hypertension was in accordance with the 2023 “Guidelines for the Prevention and Treatment of Hypertension in China.” Hypertension was defined as a systolic blood pressure ≥140 mmHg or a diastolic blood pressure ≥90 mmHg.

#### Diabetes

Diabetes was in accordance with the 2023 American Diabetes Association Diabetes Diagnosis and Treatment Standards. With regard to glucose testing, diabetes was considered as a fasting blood sugar level of ≥7.0 mmol/L, or an oral glucose tolerance test result or blood glucose level in 2 hours after a meal of ≥11.1 mmol/L, with or without clinical symptoms such as polydipsia, overeating, polyuria, and weight loss.

### Ethical Considerations

All the human experiments were performed in accordance with the principles of the Declaration of Helsinki. All experimental protocols were approved by the Research Ethics Committee of Pingshan Hospital of Southern Medical University (approval number: KY-2019-001-01). The personal information of samples involved in the study was not disclosed.

### Statistical Methods

SPSS 20.0 software (IBM Corp) was used to establish a database and conduct statistical analysis. Measurement data have been expressed as mean (SD), and comparisons of these data before and after the intervention were performed using a paired *t* test. Count data have been expressed as rate or constituent ratio, and comparisons of these data before and after the intervention were performed using a paired chi-square test. Differences with *P*≤.05 were considered statistically significant.

## Results

### Demographic Characteristics

The total population in the experimental streets before the intervention was 118,126, including 70,603 men and 46,980 women (men-to-women ratio of 1:0.665), and there were 5713 (4.8%) elderly people aged older than 60 years. Among the residents, 27,275 (23.1%) had a primary school or lower education level, 43,801 (37.1%) had a junior high school education level, 23,814 (20.2%) had a high school (technical secondary school) degree, and 23,235 (19.7%) had a university or higher education level. There were 3566 patients with hypertension and 1200 patients with diabetes registered in the community health service center.

### Residents’ Medical Treatment Situation

The number of chronic patients transferred from community health service centers to hospitals has increased from 79 in 2020 to 1634 in 2021 and 2381 in 2022 through digital health management. The number of patients transferred from hospitals to community health service centers has increased from 185 in 2020 to 1760 in 2021 and 3569 in 2022. The number of elderly people aged older than 65 years in the experimental streets who actively go to community health service centers for physical examination increased from 4876 in 2020 to 5579 in 2021 and 6809 in 2022.

### Changes in Residents’ Health Literacy

**Figure 6:**
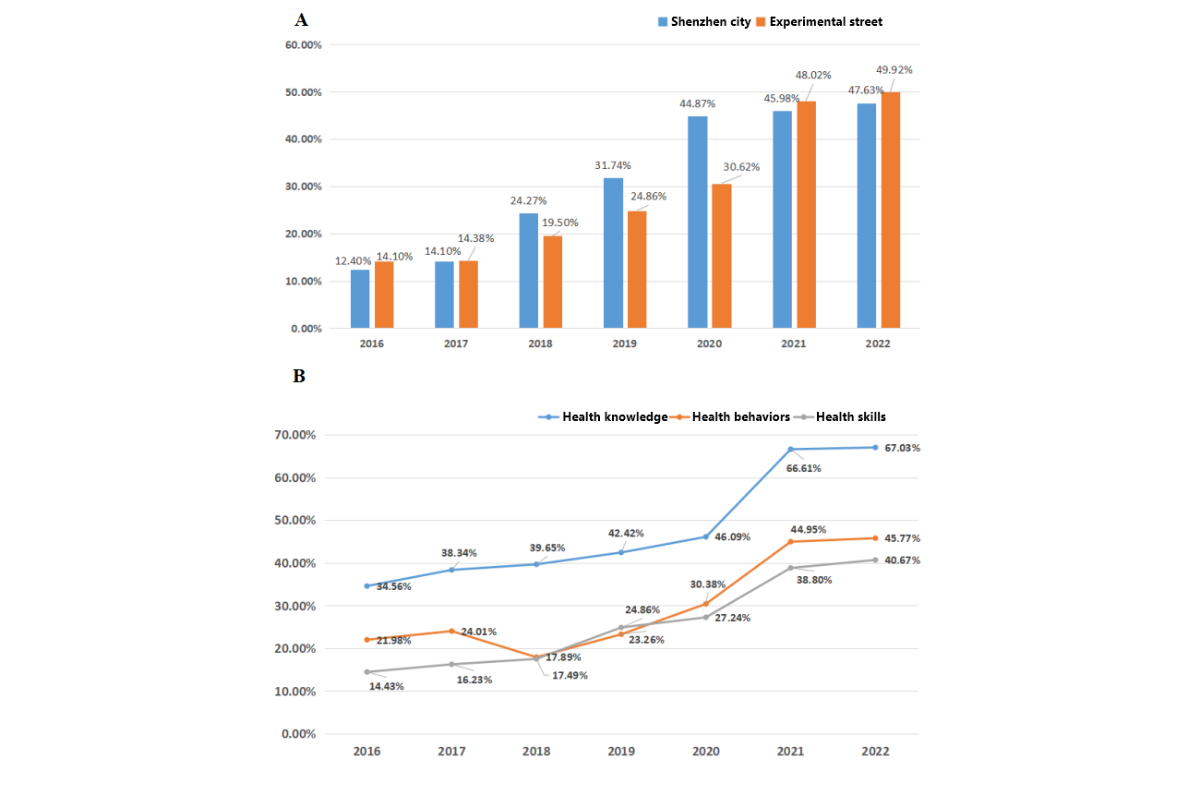
Changes in the health literacy of residents before and after the intervention. (A) Health literacy of residents in Shenzhen city and the experimental streets from 2016 to 2022. (B) Health knowledge, behavior, and skills of residents in the experimental streets from 2016 to 2022.

In order to monitor residents’ health literacy, a health literacy questionnaire survey was conducted annually among 1000 adult residents aged 18 years or older in our region. The survey results showed that the health literacy of residents in the experimental streets rose slowly from 2017 to 2019, which was lower than the average level of residents in the city. The health literacy of residents in 2019 was 30.6% (3062/10,000). After the 2-year intervention, residents’ health literacy scores significantly improved. In 2022 and 2023, residents’ health literacy reached 48.0% (4802/10,000) and 49.9% (4992/10,000), respectively, with significant differences (*P*<.001). Residents’ health knowledge, health behavior, and health technology literacy showed significant improvements (Figure 6).

### Changes in Residents’ Risks of ASCVD

The residents’ risks of ASCVD in the experimental streets were evaluated by the RACCVD, SRSC, ASCVD, FRS, and SCORE scales. We selected 6996 adult residents (those with hypertension or diabetes, or those aged older than 65 years) who completed the annual physical examination in 2020 and 2022 for ASCVD risk assessment. The results showed that after the 2-year intervention, the risk of ASCVD among the residents reduced, and the high-risk rate of residents in 2022 assessed by the 5 assessment models significantly reduced (*P*<.001) (Table 1).

**Table 1:** Risk of atherosclerotic cardiovascular disease in residents with hypertension or diabetes, or those aged older than 65 years before and after the intervention.

Assessment tool	Year 2020 (before; n=6996), n (%)				Year 2022 (after; n=6996), n (%)				*P* value
	Low risk	Medium risk	High risk or extremely high risk	Low risk		Medium risk	High risk and extremely high risk		
RACCVD^a^	2648 (37.9)	3067 (43.8)	1281 (18.3)	2977 (42.5)		3121 (44.6)	898 (12.8)	<.001	
SRSC^b^	2534 (36.2)	1454 (20.8)	3011 (43.0)	2892 (41.4)		1226 (17.5)	2873 (41.1)	<.001	
ASCVD^c^	3178 (45.4)	1652 (23.6)	2166 (31.0)	3572 (51.1)		1022 (14.6)	2402 (34.3)	<.001	
FRS^d^	4260 (60.9)	2113 (30.2)	623 (8.9)	4431 (63.3)		2128 (30.4)	437 (6.2)	<.001	
SCORE^e^	2708 (38.7)	3267 (46.7)	1021 (14.6)	3115 (44.5)		3088 (44.1)	793 (11.3)	<.001	

^a^RACCVD: risk assessment model for cardio-cerebrovascular disease.

^b^SRSC: Stroke Risk Scorecard.

^c^ASCVD: 10-year atherosclerotic cardiovascular disease.

^d^FRS: Framingham scale.

^e^SCORE: Systematic Coronary Risk Evaluation.

### Management Effect of Hypertension and Type 2 Diabetes Before and After the Intervention

#### Coverage of Patients With Hypertension and Type 2 Diabetes

The number of managed patients with hypertension was 3566 in 2020 and increased to 4285 in 2022. The standardized management rate of community hypertension increased from 59.6% (2124/3566) in 2020 to 75.0% (3212/4285) in 2022. The number of managed patients with diabetes increased from 1200 in 2019 to 2430 in 2022. The standardized management rate increased from 55.8% (670/1200) in 2020 to 69.4% (1686/2430) in 2022.

#### Effect of Blood Pressure and Blood Glucose Control

A total of 2091 patients with hypertension and 888 patients with diabetes who were enrolled in 2020 and who completed the 2-year internet-based management were statistically analyzed. The average blood pressure of patients with hypertension decreased from 148/96 mmHg before the intervention to 137/82 mmHg after the intervention, and the blood pressure control rate of patients with hypertension increased from 51.7% (1081/2091) to 81.2% (1698/2091). The average fasting blood glucose and glycated hemoglobin levels of patients with diabetes were 7.9 mmol/L and 8.2%, respectively, before the intervention and decreased to 6.4 mmol/L and 6.3%, respectively, after the intervention, and the blood sugar control compliance rate increased from 42.0% (373/888) to 73.0% (648/888).

#### Changes in Physical Examination Indicators

We evaluated the changes in patients’ physical examination indicators before and after the intervention. The BMI, waist circumference, blood uric acid, TC, triglyceride, and serum LDL-C values of patients with hypertension decreased significantly after the intervention compared with the values before the intervention (*P*<.05). The BMI, waist circumference, aspartate aminotransferase, TC, triglyceride, and LDL-C values of patients with diabetes decreased significantly after the intervention compared with the values before the intervention (*P*<.05). The data are presented in Tables 2 and 3.

**Table 2:** Changes in physical examination indicators in patients with hypertension (n=2091).

Variable	Before the intervention, mean (SD)	After the intervention, mean (SD)	*P* value
BMI (kg/m^2^)	25.67 (5.58)	23.52 (3.86)	<.001
Waist circumference (cm)	87.55 (12.28)	84.11 (13.12)	<.001
Aspartate transaminase (U/L)	25.50 (23.76)	25.07 (3.35)	.41
Alanine transaminase (U/L)	23.73 (11.27)	23.45 (10.22)	.40
Blood uric acid (μmol/L)	395.9 (99.52)	345.10 (64.23)	<.001
Total cholesterol (mmol/L)	5.15 (1.17)	4.61 (1.01)	<.001
Triglyceride (mmol/L)	2.23 (1.70)	2.12 (1.62)	.03
LDL-C^a^ (mmol/L)	2.80 (1.03)	2.23 (1.01)	<.001
HDL-C^b^ (mmol/L)	1.49 (0.54)	1.50 (0.42)	.50

^a^LDL-C: low-density lipoprotein cholesterol.

^b^HDL-C: high-density lipoprotein cholesterol.

**Table 3:** Changes in physical examination indicators in patients with type 2 diabetes (n=888).

Variable	Before the intervention, mean (SD)	After the intervention, mean (SD)	*P* value
BMI (kg/m^2^)	24.67 (3.61)	23.14 (4.52)	<.001
Waist circumference (cm)	86.27 (13.24)	81.74 (11.85)	<.001
Aspartate transaminase (U/L)	26.22 (16.41)	37.81 (12.66)	<.001
Alanine transaminase (U/L)	21.35 (10.22)	21.01 (12.54)	.53
Blood uric acid (μmol/L)	363.10 (85.83)	341.20 (71.96)	<.001
Total cholesterol (mmol/L)	5.27 (1.18)	4.71 (1.25)	<.001
Triglyceride (mmol/L)	2.33 (2.41)	2.11 (1.42)	.02
LDL-C^a^ (mmol/L)	2.86 (1.07)	2.41 (1.14)	<.001
HDL-C^b^ (mmol/L)	1.29 (1.53)	1.33 (2.56)	.69

^a^LDL-C: low-density lipoprotein cholesterol.

^b^HDL-C: high-density lipoprotein cholesterol.

#### Changes in Medical Behavior

The self-measured blood pressure and blood glucose behaviors among the patients were investigated at the time of enrollment and after the 2-year internet-based management. At the time of enrollment, 25.4% (531/2091) and 26.0% (231/888) of patients with hypertension and type 2 diabetes, respectively, self-measured their blood pressure and glucose regularly (more than once a week). After the 2 years of management, 71.2% (1489/2091) and 69.4% (616/888) of patients with hypertension and type 2 diabetes, respectively, self-measured their blood pressure and glucose regularly, with a statistically significant difference (*P*<.001). The MCQ was used to evaluate patient compliance. After the 2-year intervention, the drug compliance of patients with hypertension increased from 33.6% (703/2091) to 73.30% (1532/2091), while that of patients with diabetes increased from 36.0% (320/888) to 75.8% (673/888) (*P*<.001). The data are presented in Table 4.

**Table 4:** Changes in medical behavior and life behavior among patients with hypertension and type 2 diabetes.

Variable		Before the intervention, n (%)	After the intervention, n (%)	*P* value
**Patients with hypertension (n=2091)**				
	Self-measured blood pressure regularly	531 (25.4)	1489 (71.2)	<.001
	Drug compliance	703 (33.6)	1532 (73.30)	<.001
	Regular exercise	740 (35.4)	1296 (62.0)	<.001
	Balanced dietary behavior	1641 (78.5)	1790 (85.6)	<.001
	Smoking	309 (14.8)	217 (10.4)	<.001
	Drinking	226 (10.8)	222 (10.6)	.84
**Patients with type 2 diabetes (n=888)**				
	Self-measured glucose regularly	231 (26.0)	616 (69.4)	<.001
	Drug compliance	320 (36.0)	673 (75.8)	<.001
	Regular exercise	359 (40.4)	713 (80.3)	<.001
	Balanced dietary behavior	687 (77.4)	786 (88.5)	<.001
	Smoking	125 (14.1)	117 (13.2)	.58
	Drinking	263 (29.6)	137 (15.4)	<.001

#### Changes in Life Behavior

Balanced dietary behavior, regular exercise, and smoking control among patients with hypertension significantly improved after the 2-year intervention than before the intervention (balanced diet rate: 1790/2091, 85.6% vs 1641/2091, 78.5%; regular exercise rate: 1296/2091, 62.0% vs 740/2091, 35.4%; smoking rate: 217/2091, 10.4% vs 309/2091, 14.8%; *P*<.001). Similarly, balanced diet behavior, regular exercise, and drinking behavior among patients with diabetes significantly improved after the 2-year intervention than before the intervention (balanced diet rate: 786/888, 88.5% vs 687/888, 77.4%; regular exercise rate: 713/888, 80.3% vs 359/888, 40.4%; drinking rate: 137/888, 15.4% vs 263/888, 29.6%; *P*<.001). On the other hand, drinking behavior among patients with hypertension and smoking behavior among patients with diabetes did not significantly improve. The data are presented in Table 4.

## Discussion

### Principal Findings

Active health is a new medical model initiated by Chinese scholars, which emphasizes the active health idea of “active discovery, scientific evaluation, active adjustment, and health promotion” [24,25]. From the perspective of the concept of active health, for individuals, active health is a medical model that promotes people’s subjective initiative for health through effective methods, focuses on actively improving health literacy, focuses on improving health behavior, and actively monitors and intervenes in the human body through various medical means to promote health and eliminate diseases. For the population, active health is a new health model that adheres to government leadership, fully mobilizes the enthusiasm of the entire society, emphasizes that individuals are the “first responsible person” for health, and promotes a healthy lifestyle with the support of new technologies such as informatics and biomics, achieving effective monitoring, accurate prediction, and active intervention, and promoting the health of the whole population. This model advocates the cooperation of governments, enterprises, medical institutions, individuals, and other parties, moving from passive medical care to active health care for everyone [9,26,27]. Active health has become one of the hot topics for scholars in China.

In recent years, many scholars in China have explored factors related to active health, such as the integration of medicine and sports, community chronic disease management, wearable active health devices, home care, smart communities, and biomechanics. However, most involved theoretical research, while empirical research was scarce [28]. There are currently no reports involving digital active health for an experimental street. Research has shown that digital medicine is an inevitable trend for the management of community chronic diseases. In developed countries, chronic disease management based on internet technology has been widely applied, and good results have been achieved. Our research results also showed that internet-based management had a positive effect on improving the compliance of chronic disease patients and improving their health behaviors.

A recent study [29] showed that the prevalence of cardiovascular risk factors has increased significantly in China from 2002 to 2018, with obesity increasing from 7.1% in 2002 to 16.4% in 2018, overweight/obesity increasing from 29.9% to 50.7%, hypertension increasing from 18.8% to 27.5%, diabetes increasing from 2.6% to 11.9%, and dyslipidemia increasing from 18.6% to 35.6%. The mortality rate of chronic noncommunicable diseases has increased from 533/100,000 in 2012 to 685/100,000 in 2018, an increase of 28.5% [29]. More than 50% of Chinese men currently smoke, and an increasing number of adults lack physical activity. According to research [29,30], the prevalence of chronic noncommunicable diseases in China is rising, while management is stagnant. It is estimated that the prevalence and management of obesity, hypertension, and diabetes in 2030 will not be appropriate, and global or national goals will not be achieved. Therefore, there is an urgency to implement effective intervention models to improve the living behavior of Chinese residents and enhance the effectiveness of chronic disease control. Previous research [12,26] found that the majority of chronic disease patients in China are currently identified by social and family health records, hospital physical examinations, and treatment processes. However, the discovery and management of patient health issues have been relatively delayed, and patient participation in their own health management is not high. At present, China has established various methods to collect patient health information. However, community health service data are difficult to summarize and integrate with another platform, forming many information islands [30,31]. Therefore, carrying out internet-based active health management based on internet technology assistance, which integrates public health and clinical services, is an important approach to achieve intelligent health management of chronic diseases throughout the cycle.

Based on the current situation and existing problems of chronic diseases in China, our team built a digital health platform, and to our knowledge, we are the first to build an active health information platform and carry out digital active health management of the community in China. Our active health information platform could fully use information technology to enrich chronic disease prevention and treatment methods and work content, and promote network service applications such as appointment diagnosis and treatment, online follow-up, disease management, and health management. It effectively realized the interconnection of residents’ wearable devices, residents’ applets, community health service center information systems, hospital information systems, and government information systems. To our knowledge, it is rare in China to achieve the interconnection and sharing of this information. Through the 2-year application of the digital health platform, we effectively improved the efficiency of the 2-way referral of patients in the experimental streets between community health service centers and hospitals, and effectively improved the effectiveness of community chronic disease management.

We used the applet and network resources developed, and through deep cooperation between the government and health institutions, we took multiple measures to create an active and healthy environment for the residents on the experimental streets. We organized a large number of comprehensive active health activities for the residents. Medical institutions took the initiative to provide a series of active health services for community residents. After the 2-year continuous intervention, the levels of health knowledge, health behavior, and health technology literacy among residents significantly improved, and the total health literacy of residents increased from 30.6% before the intervention to 49.9% after the intervention. The practice has proven that the improvement of residents’ health knowledge and health behavior is a long process that requires the assistance of multiple departments and the implementation of multichannel and sustained health education and promotion. An effective auxiliary tool is an important element in improving the effectiveness of health education. The results of this study suggest that with the use of convenient information platforms, residents can easily record their home testing results and smoking and drinking behaviors, and evaluate their health status. Moreover, health education and health services from the family doctor team can be easily obtained through an applet. These functions can encourage residents to actively obtain health knowledge, actively monitor health indicators, and actively participate in health interventions, which could play a positive role in improving residents’ health literacy and improving health behaviors.

We realized that risk assessment and management are very important in health management [32,33]. Therefore, in the active health information platform, we established a risk management information system and configured various evaluation tools in the system, including our self-developed evaluation model and widely used stroke cards and scales (ASCVD, SCORE, and FRS) from domestic and international sources. The system could interconnect the “i Active Health” applet, community health information system, hospital information system, and network map. The patient’s risk assessment score and risk level of cardio-cerebrovascular disease could be automatically calculated in real time, allowing early identification of high-risk groups, accurate positioning, early warning, and early diagnosis and treatment. In the past 2 years, we conducted ASCVD risk assessment and hierarchical management for adult residents who completed annual health examinations in community medical institutions and were registered in community health service centers. The practice has proven that we have effectively reduced the risk of ASCVD among residents in the experimental streets by virtue of the information platform, collected health information of residents in an all-round way through the medical team, and carried out health risk assessment and hierarchical management. This was a useful attempt to carry out the entire process management of ASCVD.

Focusing on the problems of a low disease control rate, low medication compliance, and unhealthy life behavior of chronic disease patients, we built a refined management model of hypertension and diabetes based on internet technology, and established a multidisciplinary management team consisting of general practitioners, community nurses, health managers, specialists, clinical pharmacists, dietitians, and sports rehabilitation specialists. Online and offline fine management for patients with hypertension and diabetes in the community were provided. We selected 2091 patients with hypertension and 888 patients with diabetes who were enrolled in 2020, and they completed a 2-year intervention for statistical analysis. The results showed that after the 2-year internet-based management for hypertension and diabetes, the patients’ blood pressure and blood sugar decreased significantly, and their control rate of blood pressure and blood sugar increased significantly. Moreover, patients’ BMI, waist circumference, blood uric acid, total cholesterol, and serum LDL-C decreased after the intervention compared to the values before the intervention. Furthermore, patients’ self-measured blood pressure, blood sugar, medication adherence, and other medical behaviors significantly improved after the intervention, and their healthy behaviors, such as balanced dietary habits and regular exercise, significantly increased. However, the drinking behavior of patients with hypertension did not significantly improve. To explore the reasons, we consulted relevant literature and conducted interviews with some patients. Some articles reported that internet-based hypertension health interventions did not significantly improve drinking behavior, which is consistent with the results of this study. Interviews with patients found that most patients had a significant increase in their awareness of the dangers of drinking, and their alcohol consumption was controlled after the intervention. However, drinking is an effective way of communication for Chinese people and might be a need for work [34], and they did not significantly reduce the frequency of drinking. Therefore, our further research will continue to attempt to enhance the effectiveness of an intervention for alcohol consumption behavior in patients with hypertension.

The results suggested that interdisciplinary team collaboration based on internet technology to carry out refined management of chronic diseases and create an online and offline precise health closed-loop service system might have a good effect on improving patient health behavior and improving the disease control rate [35-38]. Based on the above results, our digital health management model has a good effect on the health of both residents and patients and thus has high medical application value. However, digital medicine has not yet been widely promoted and applied worldwide though it could effectively improve the effectiveness of chronic disease management. According to the findings of this research and the literature, we have some strategic suggestions. First, further improve the convenience and equipment dependence of internet-assisted disease management technology with the help of artificial intelligence technology, and constantly improve the effectiveness and popularization of the internet-based chronic disease management model to allow more patients to participate and improve the management effect. Second, strengthen health education, change the old ideas on chronic disease management for both the chronic disease management team and patients, and encourage both doctors and patients to actively try new models of chronic disease management.

In conducting digital active health interventions for residents, we have realized 2 crucial factors. First, in-depth cooperation between the government and health institutions is important to achieve an active health system, which is essential for improving residents’ active health literacy. Usually, the work performed by the health system is related to enhancing knowledge, guiding health behaviors, and intervening in health behaviors, but it is difficult to improve the health environment. With deep participation by the government, the difficulties in improving the health environment, integrating resources, and organizing residents to participate in active health activities can be overcome. Second, the internet and artificial intelligence play crucial roles in enhancing residents’ learning and participation enthusiasm. At present, it is difficult to achieve good health intervention effects by simply using traditional health education methods, such as posters, flyers, and lectures. Therefore, digital health platforms and wearable devices, which are convenient to use, have a good sense of experience, are strongly interactive, and facilitate continuous intervention, play catalytic roles in improving the effectiveness of active health management [39,40]. We realize that the active health management model is new in China, and it is very difficult to build active health experimental streets. Moreover, we are aware that there are difficulties in promoting and applying this model. At present, there is a long process to change from “passive health” to “active health” for Chinese residents, health systems, and the government. However, the digital active health management model may provide direction for China to comprehensively carry out active health services in the future.

### Limitations

Although this study pioneered the exploration of a digital active health management model in China and achieved some satisfactory results, there were some limitations. First, owing to the funding and manpower constraints of the research group, we only selected 2 streets as experimental streets for intervention research, which may not fully represent the current community health status in China. Second, we adopted a single-arm study design for comparison before and after the intervention but were unable to establish a control street for the general population in this region. Third, this study carried out a 2-year intervention and only analyzed the residents’ health literacy, medical behavior, and life behavior, as well as the effect of hypertension and diabetes health management. It failed to compare the long-term outcomes and health economic indicators. Thus, the effectiveness of our digital active health management model needs to be further validated through studies involving larger sample sizes, longer assessment periods, and more in-depth analyses in the future.

### Conclusions

This study demonstrates that our digital health platform can effectively achieve the interconnection and exchange of different health information. The digital active health management carried out with the assistance of this platform improved the effectiveness of community chronic disease management. Thus, the platform is worth promoting and applying in practice.

## Appendix

Multimedia Appendix 1 Registration guidelines of the “i Active Health” applet.

Multimedia Appendix 2 Training materials for using the “i Active Health” applet.

Multimedia Appendix 3 Health index indicators and algorithm.

Multimedia Appendix 4 Internet-based refined management of hypertension and diabetes operation manual.

Multimedia Appendix 5 The indicator system and its calculation method of the risk assessment of cardio-cerebrovascular disease.

## Abbreviations

ASCVD: atherosclerotic cardiovascular disease
FRS: Framingham scale
HDL-C: high-density lipoprotein cholesterol
LDL-C: low-density lipoprotein cholesterol
MCQ: Medication Compliance Questionnaire
RACCVD: risk assessment model of cardio-cerebrovascular disease
SCORE: Systematic Coronary Risk Evaluation
SRSC: Stroke Risk Scorecard

## Data Availability

The datasets generated during and analyzed during this study are available from the corresponding author upon reasonable request.
