# Supplementary material for: Digital Health Platform for Improving the Effect of the Active Health Management of Chronic Diseases in the Community: Mixed Methods Exploratory Study
Source: J Med Internet Res. 2024 Nov 18;26:e50959. doi: 10.2196/50959 (PMC11612601; doi:10.2196/50959)
Supplement: Multimedia Appendix 1 [file jmir_v26i1e50959_app1.pdf]

i主动健康
i-Active Health

# “i主动健康”小程序注册指引

## STEP 01

微信扫一扫右侧二维码
或微信主页下拉搜索
小程序“i主动健康”

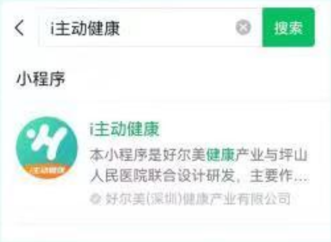


## STEP 02

进入小程序，使用手机号授权登录

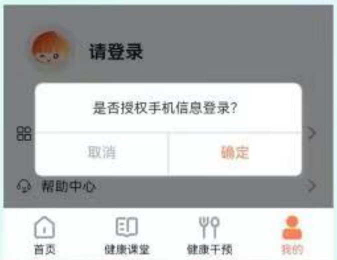


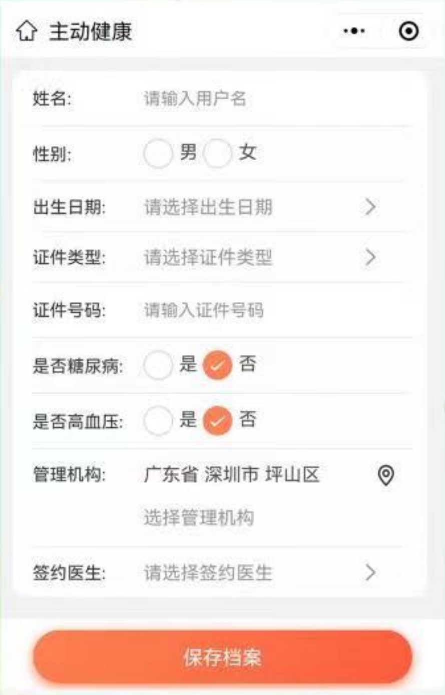


## STEP 03

①输入姓名，选择性别、出生日期

②证件类型一般选择“居民身份证”或符合的其他选项

③输入证件号码

④选择是否有高血压或糖尿病

⑤选择当前所在管理机构(XX社康)

⑥选择签约医生

⑦点击“保存档案”即为注册成功
