# Supplementary material for: Digital Health Platform for Improving the Effect of the Active Health Management of Chronic Diseases in the Community: Mixed Methods Exploratory Study
Source: J Med Internet Res. 2024 Nov 18;26:e50959. doi: 10.2196/50959 (PMC11612601; doi:10.2196/50959)
Supplement: Multimedia Appendix 2 [file jmir_v26i1e50959_app2.pdf]

南方医科大学坪山总医院
Pingshan general hospital of Southern Medical University

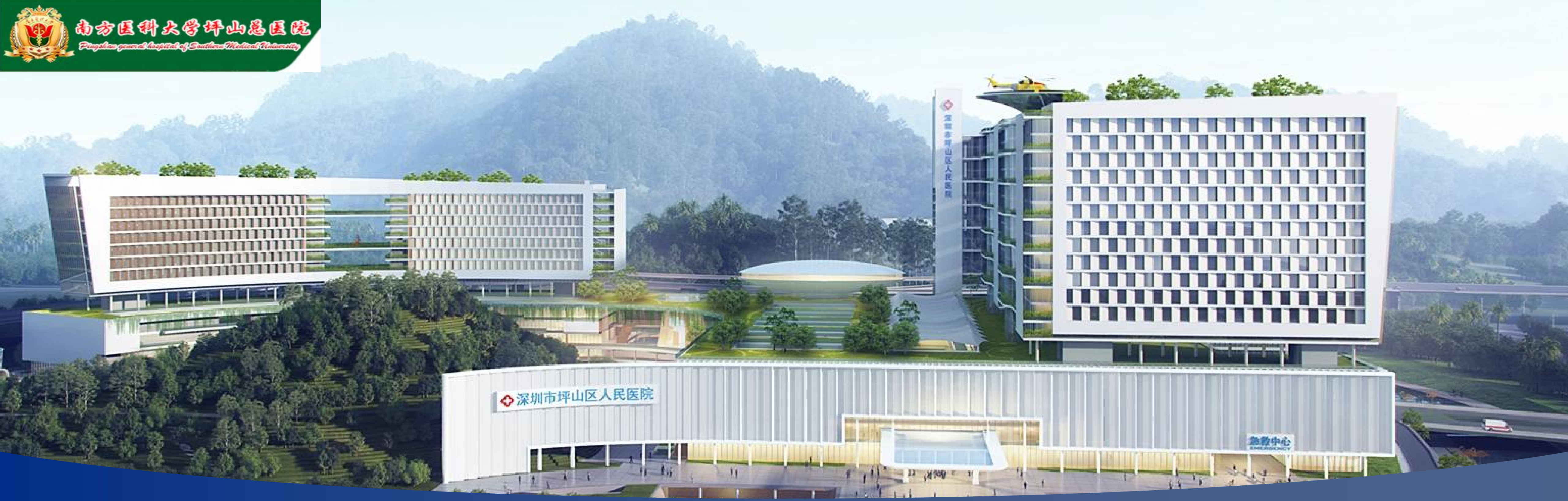

“i主动健康”小程序使用 培训资料

居民何时进行注册?

① 就诊前

② 随访时

③ 义诊时

医护人员可根据《i主动健康-小程序居民注册操作流程》引导居民在就诊前进行小程序注册。

小程序对医疗就诊系统的帮助:

- 小程序将在不久后推广到坪山人民医院及所有社康进行使用。居民通过在小程序上填写健康指数问卷，定期上传血压、血糖等体检结果，获得综合分值。
- 医护人员可根据最终得分进行风险评价。分值达到高危范围的患者将会被推送至医院系统，有利于推动高危患者的转诊和随访进程。
- 小程序联动社康系统和医院体检数据，可全面展现居民的基本健康状况，对于慢病管理更加精准有效。

i主动健康

一、小程序居民注册操作流程

一、小程序居民注册操作流程（共4步）

第一步：使用微信搜索i主动健康，或扫码进入i主动健康小程序

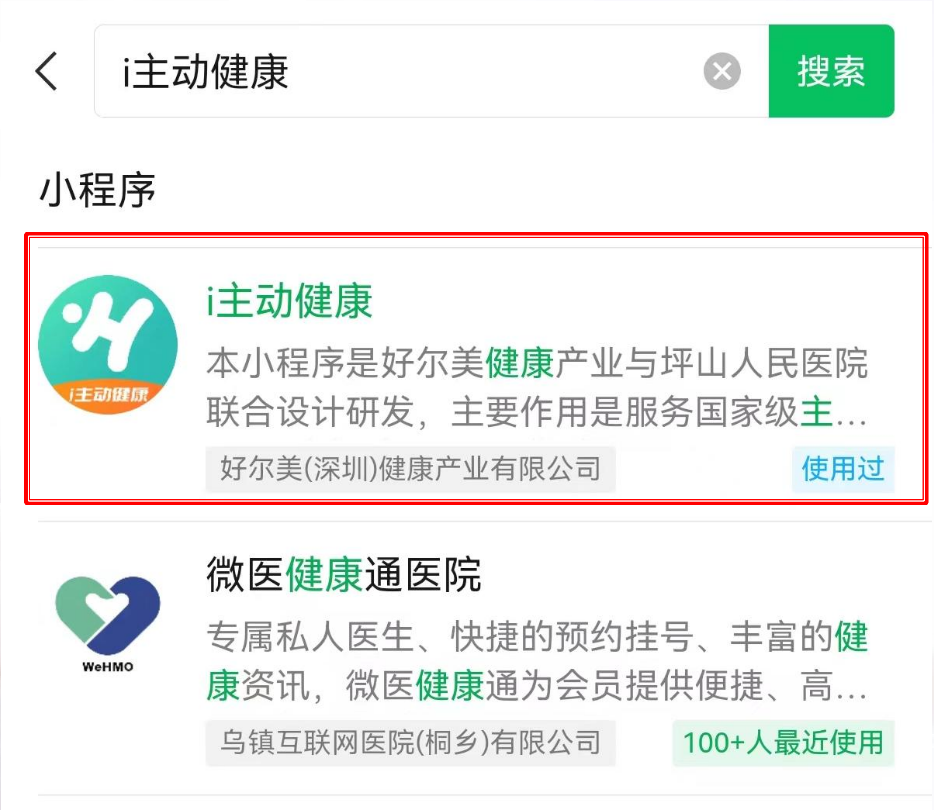

一、小程序居民注册操作流程（共4步）

第二步：微信授权登录

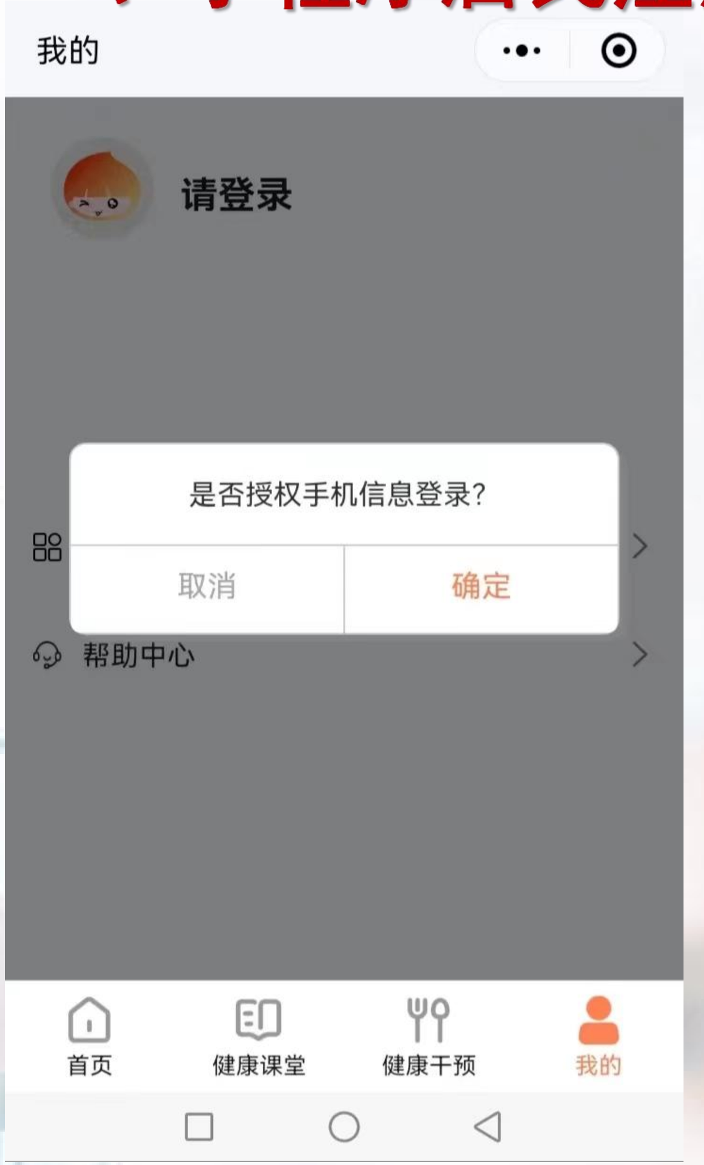

一、小程序居民注册操作流程（共4步）

第三步：点击首页左上角分数处，进入注册信息页面。绑定社康机构和签约医生。

(以下信息均为必填项)

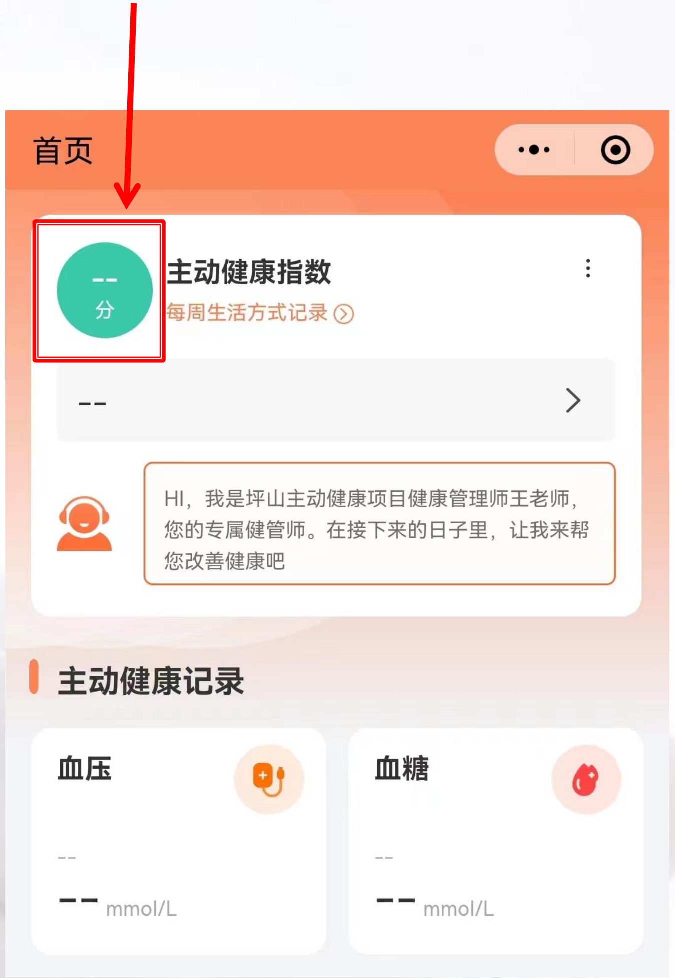

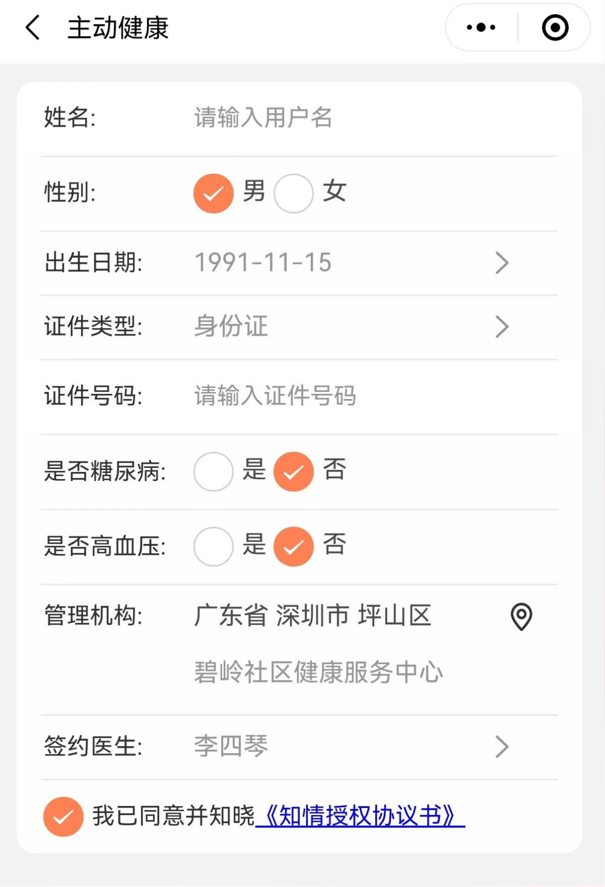

一、小程序居民注册操作流程（共4步）

第四步：填写主动健康指数测评问卷（必填项）

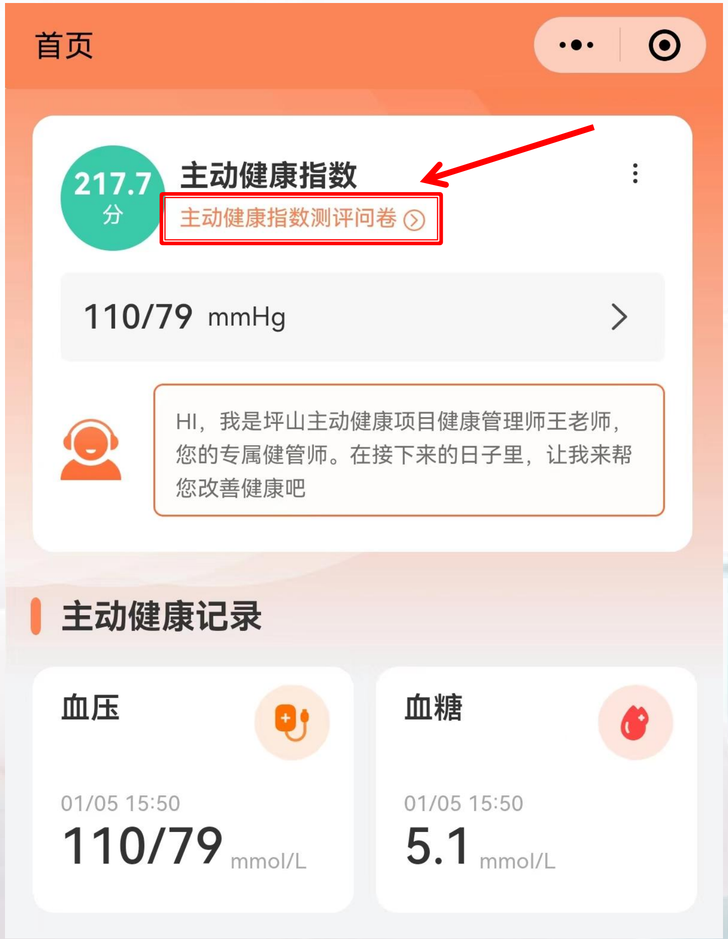

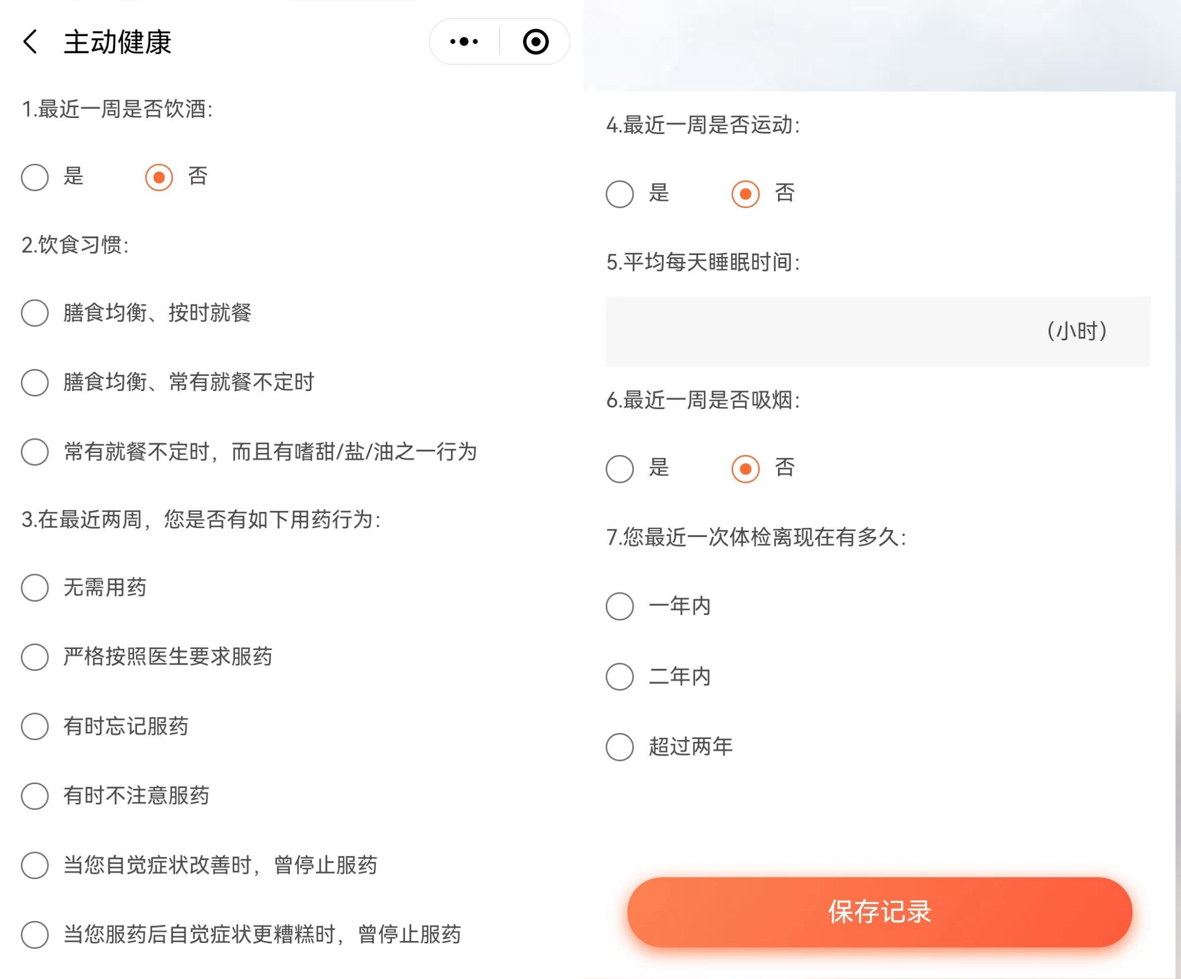

i主动健康

二、小程序居民日常使用流程

二、小程序居民日常使用流程

1、添加血压记录，可手动输入，或配合已发放的设备自动测量上传

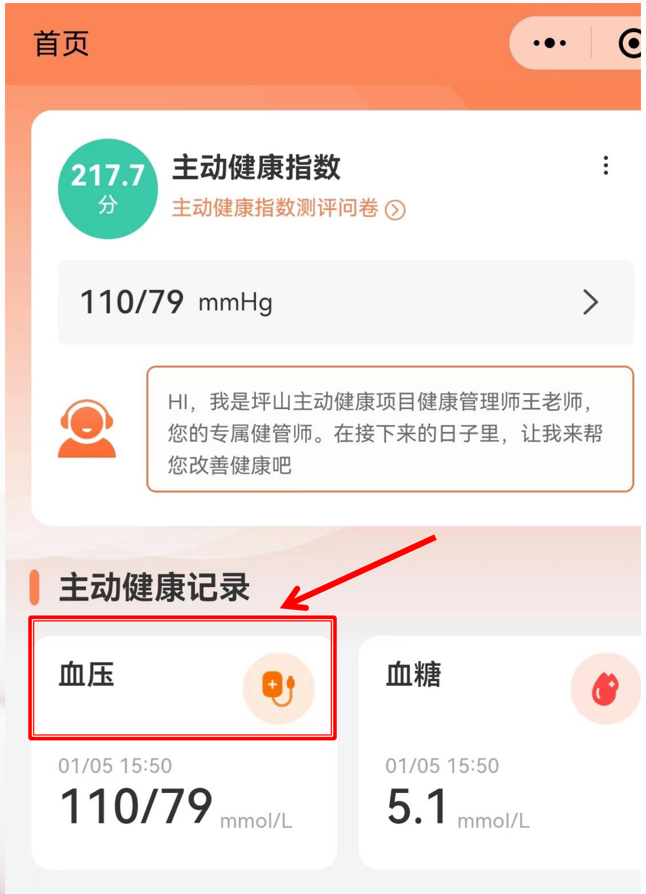

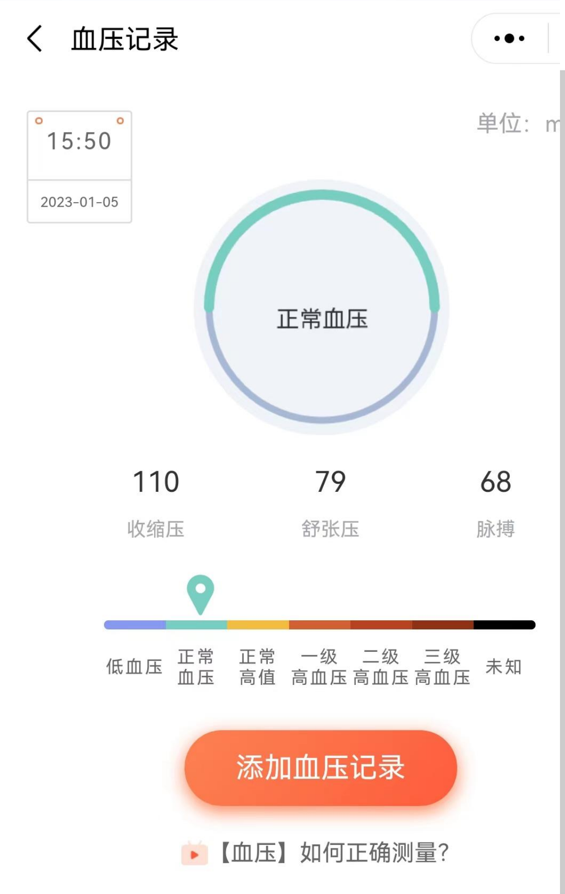

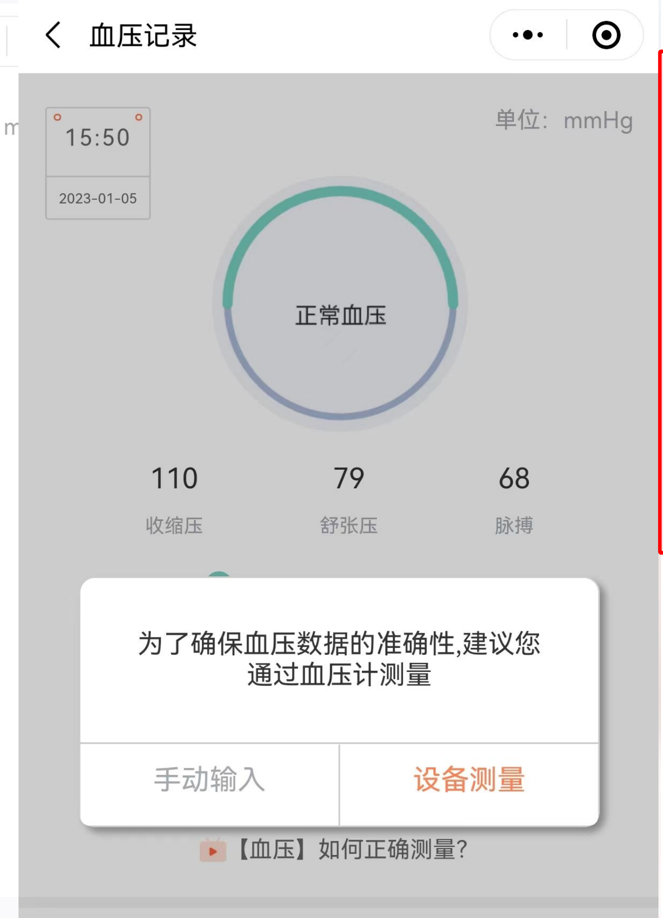

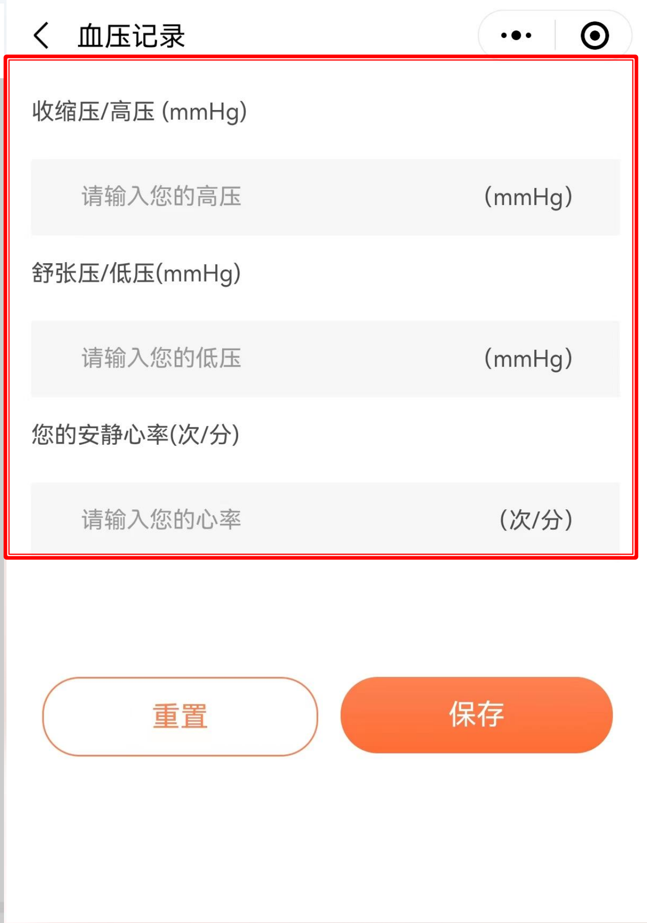

二、小程序居民日常使用流程

2、添加血糖记录，可手动输入，或配合已发放的设备自动测量上传

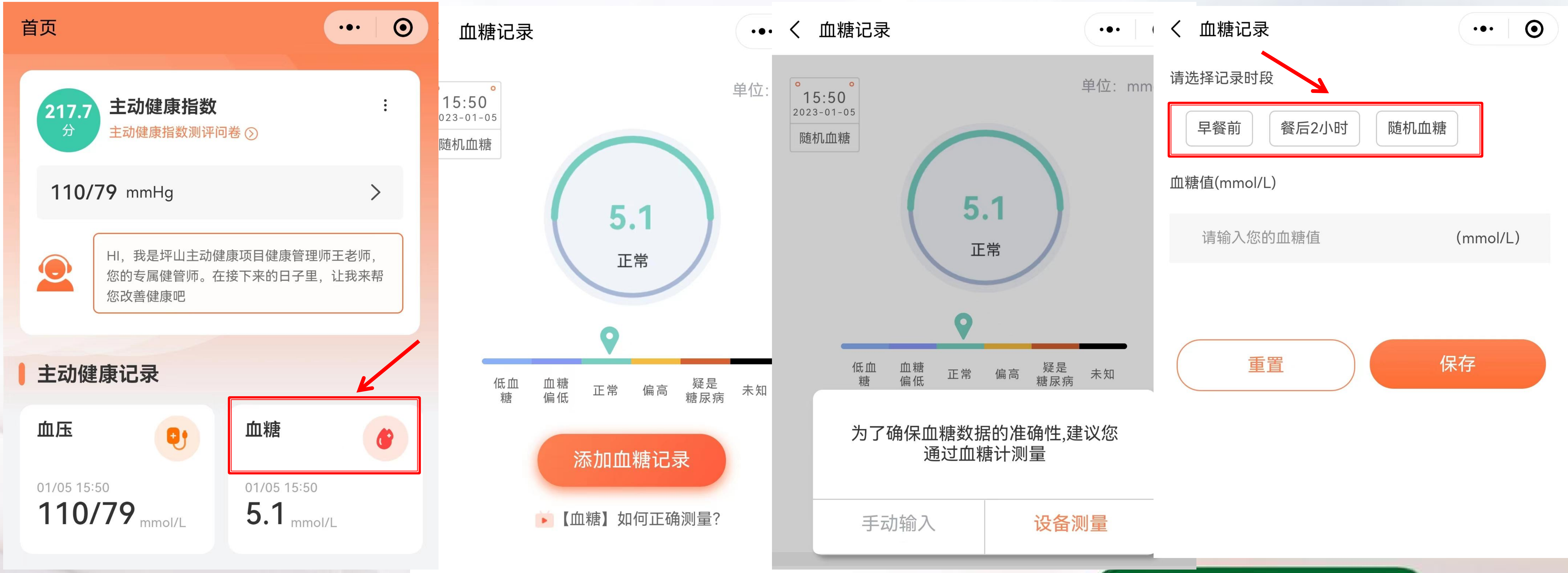

二、小程序居民日常使用流程

3、体检报告录入。若患者在坪山人民医院已有体检记录，系统将自动匹配体检结果，医生可在社康系统中查看体检数据。可手动输入。若不填写，会影响最终得分的准确性。

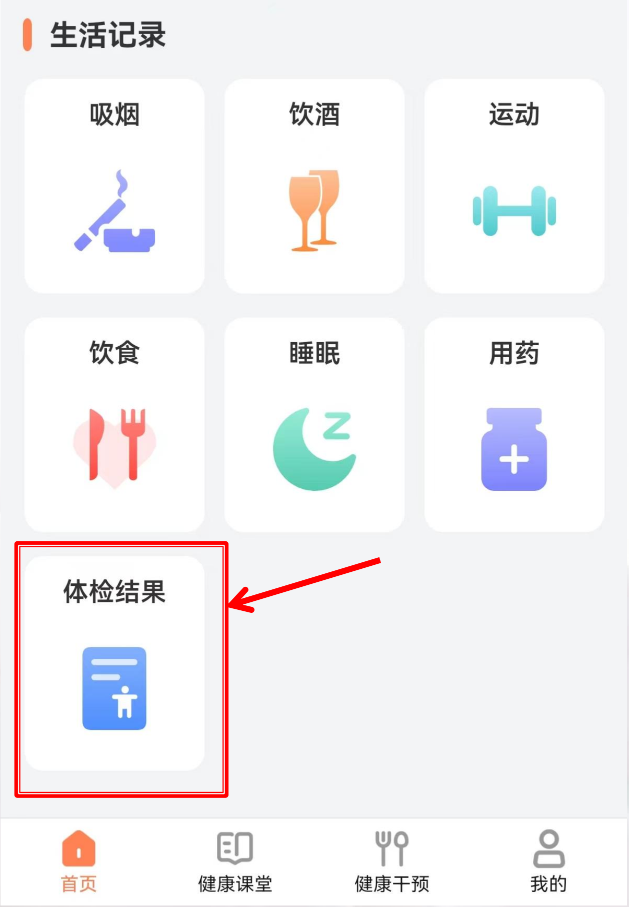

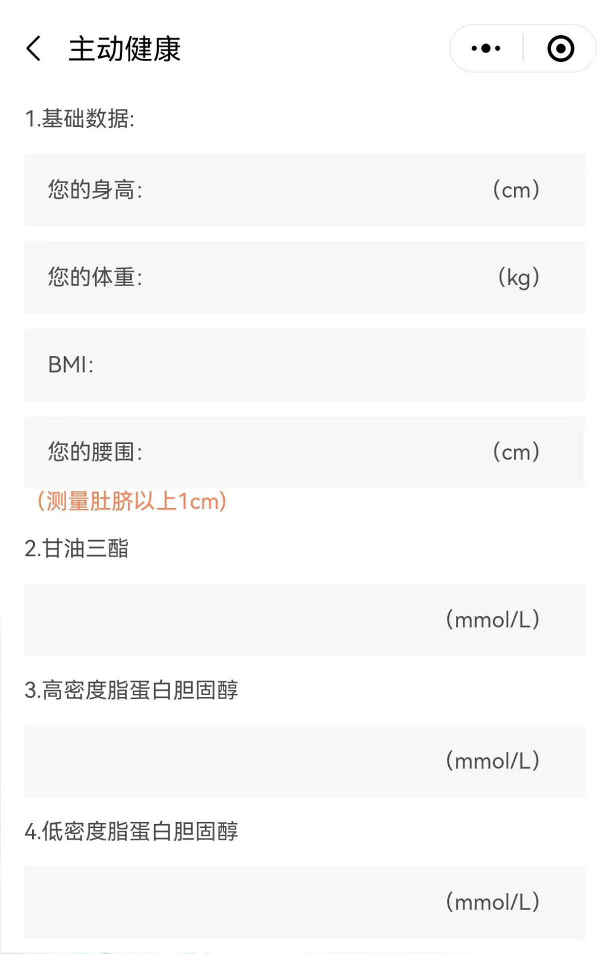

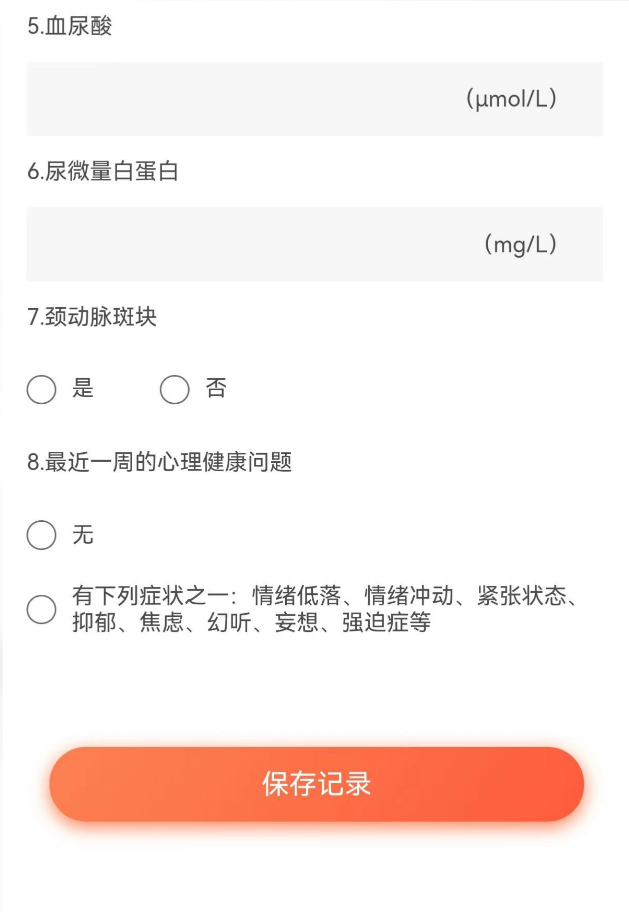

i主动健康

三、小程序医护人员操作流程

三、小程序医护人员操作流程

1.登录社康系统 http://172.26.38.210:8086/phisps/

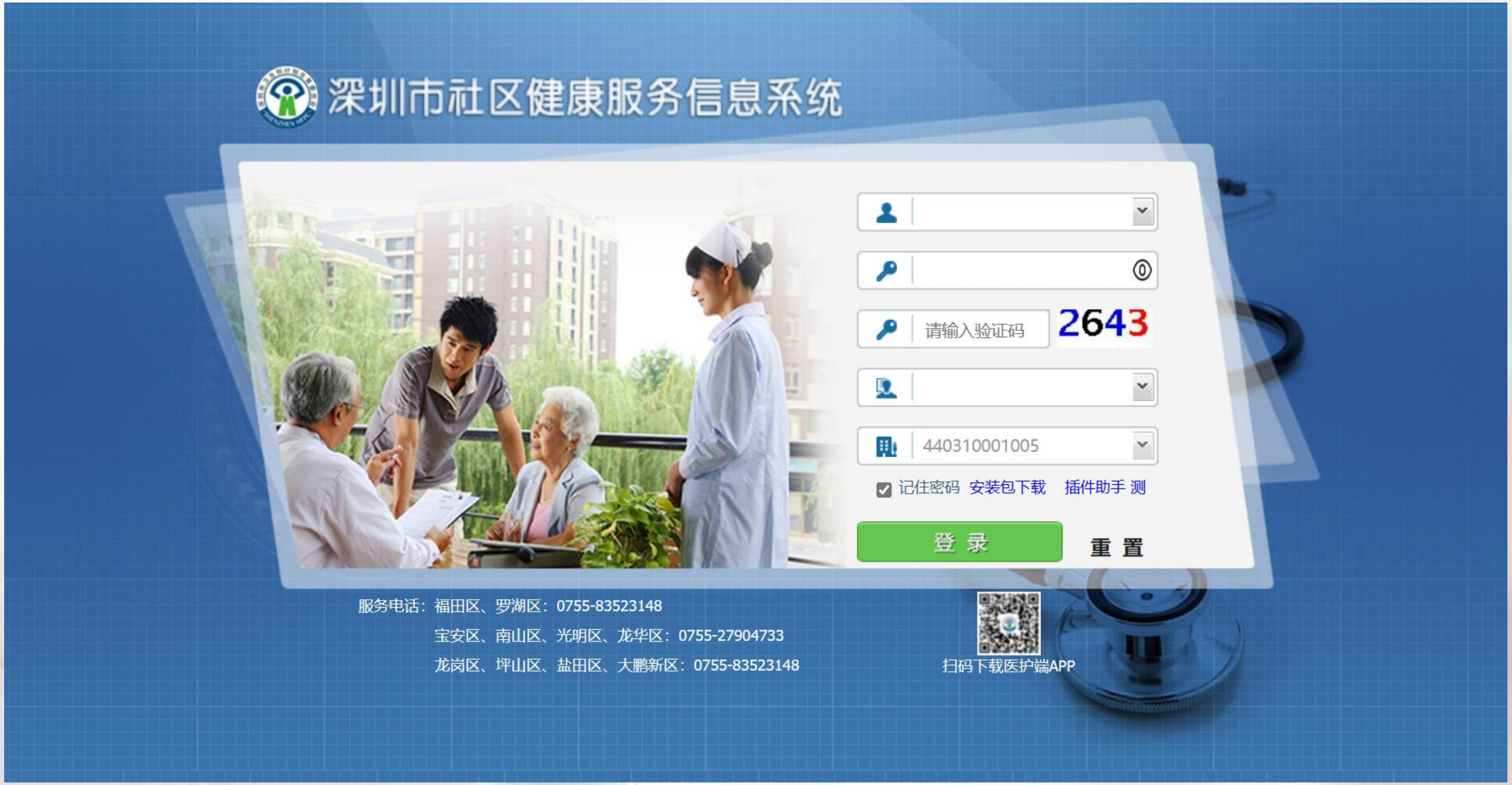

三、小程序医护人员操作流程

2.点击【档案管理】

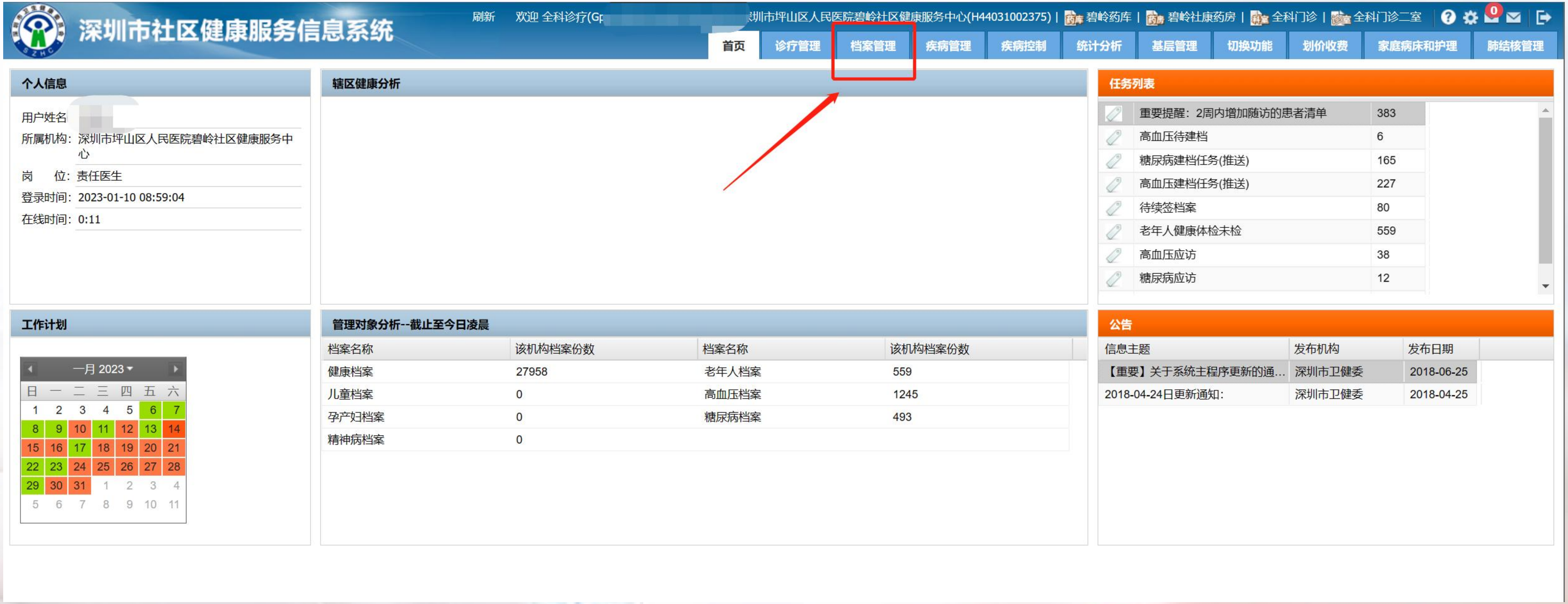

三、小程序医护人员操作流程

3.点击【重点人群管理】

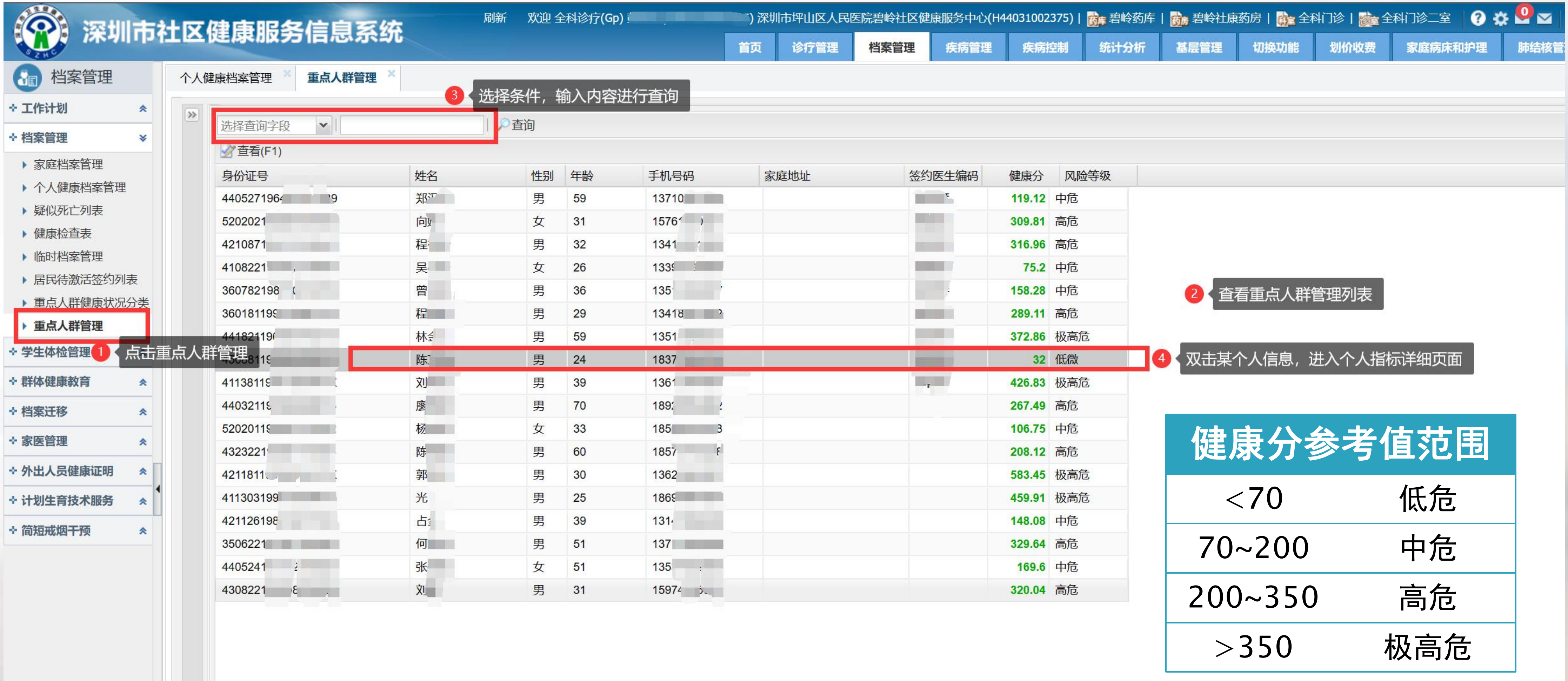

健康分参考值范围	
<70	低危
70~200	中危
200~350	高危
>350	极高危

三、小程序医护人员操作流程

4.【重点人群管理】个人各项指标数据

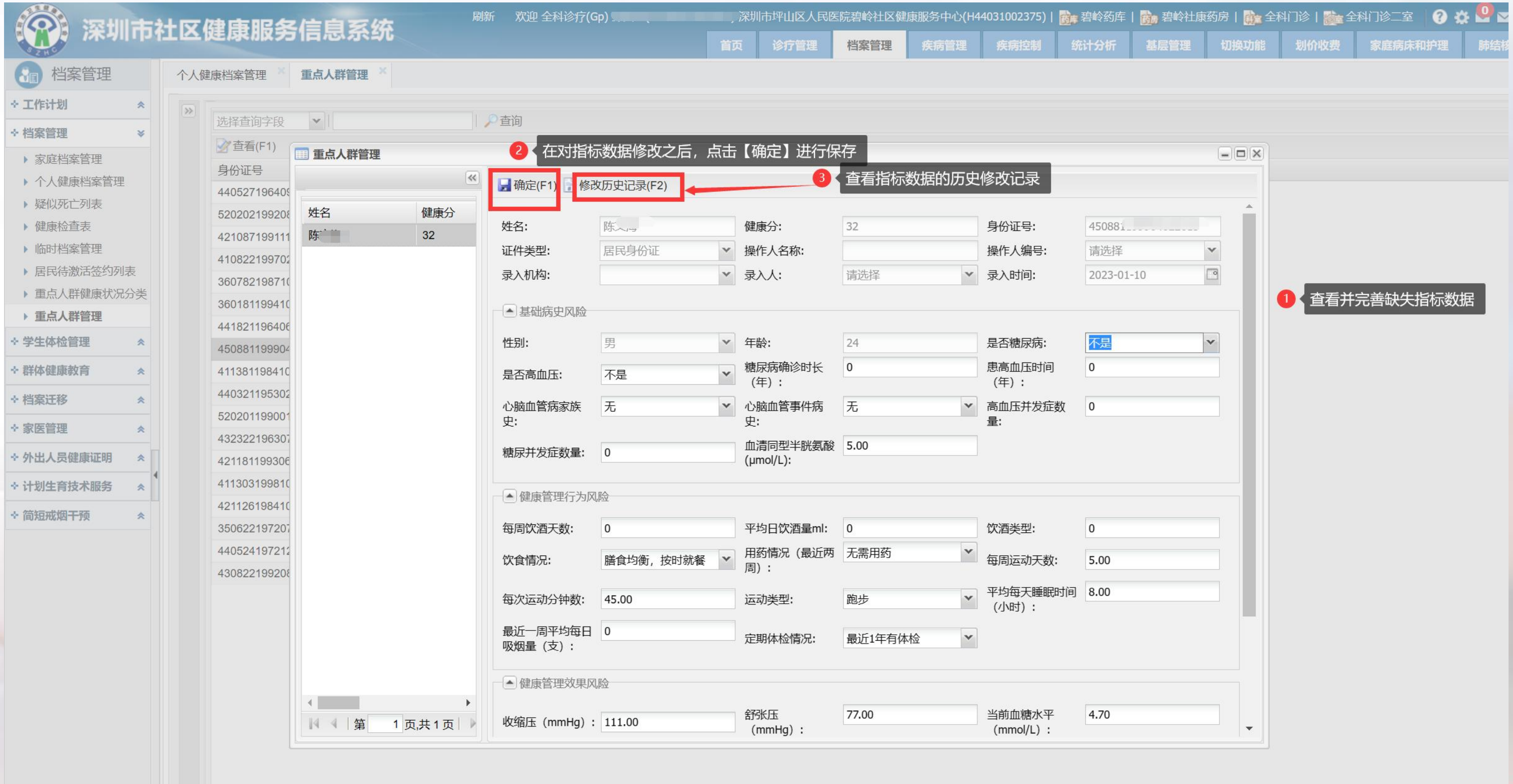

三、小程序医护人员操作流程

5.【修改历史记录】

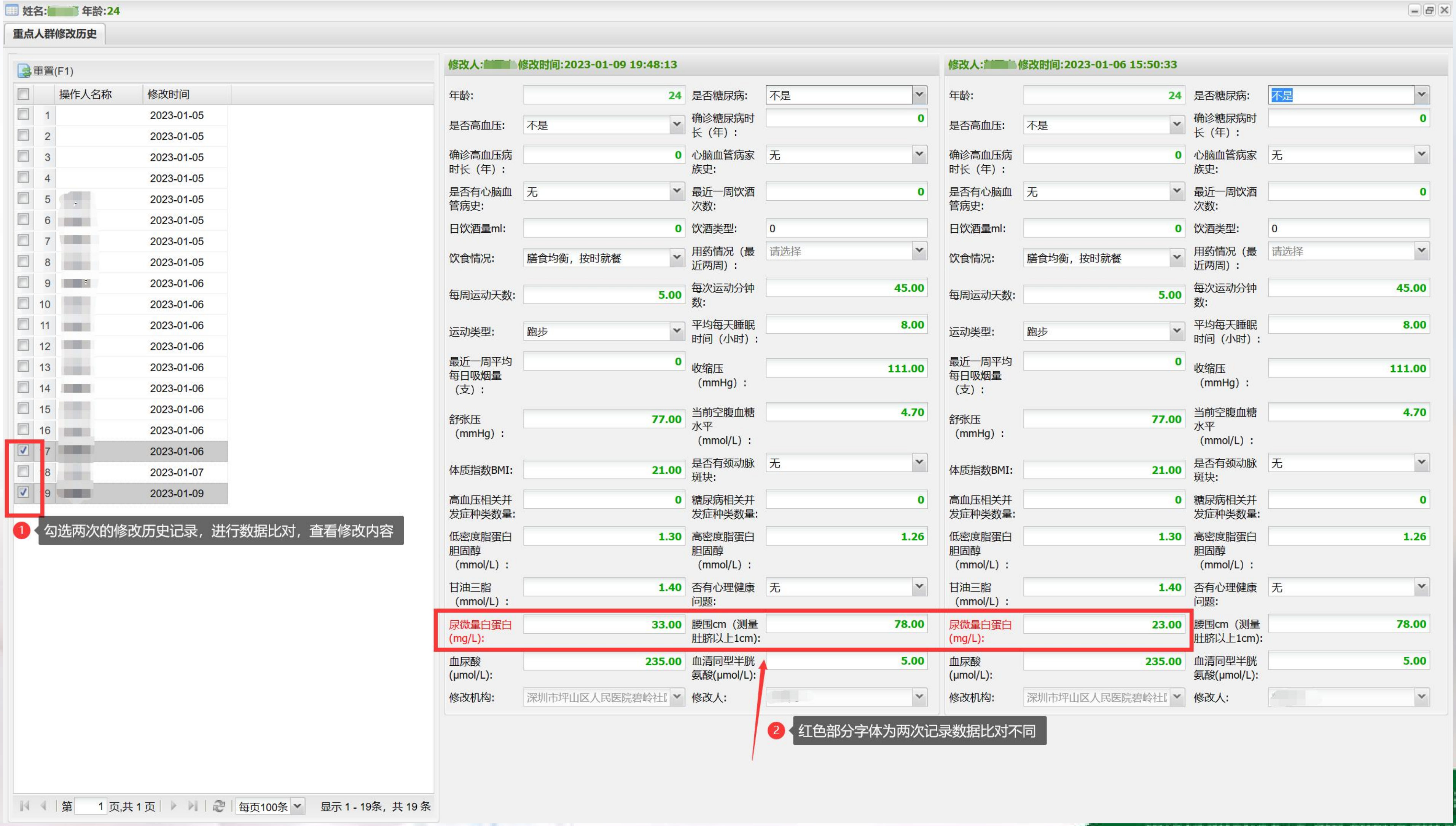

健康数据采集（血压测量规范）

1

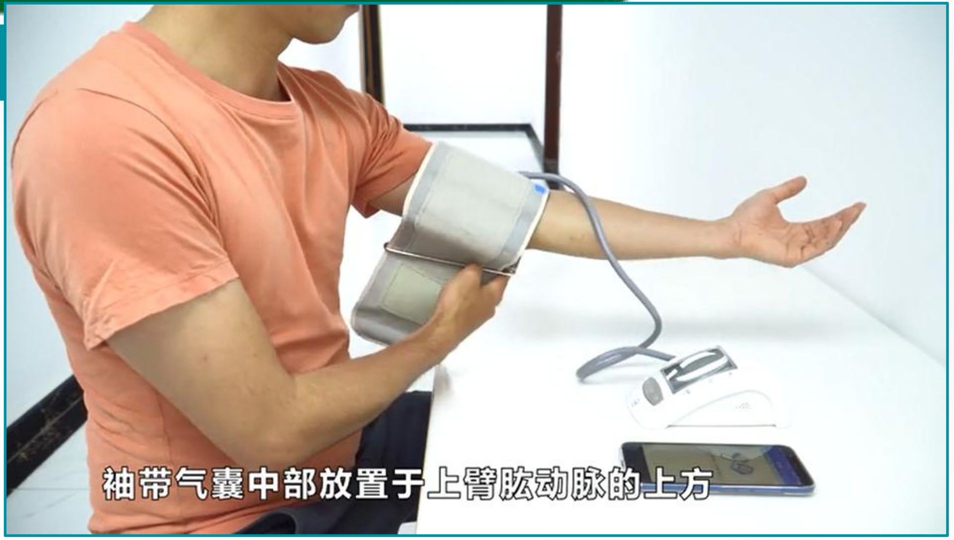

2

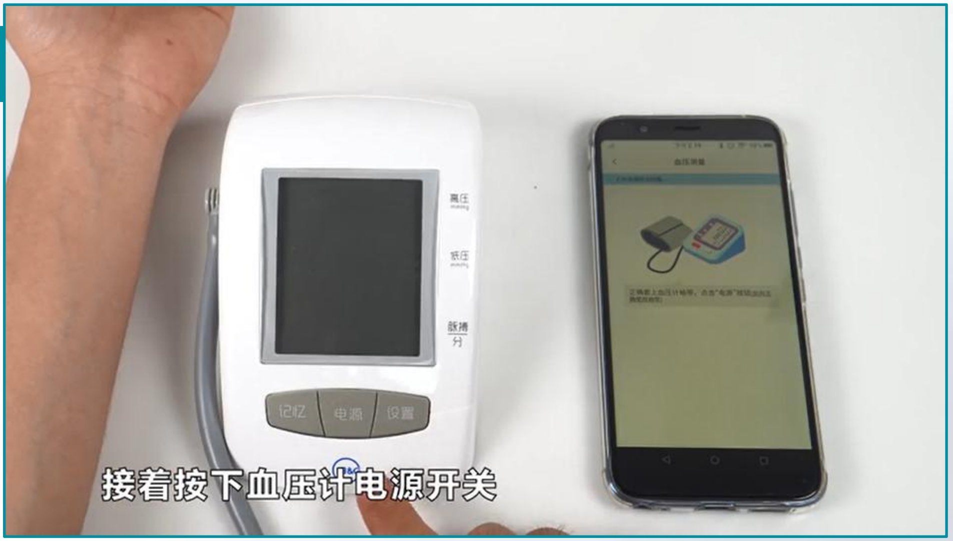

3

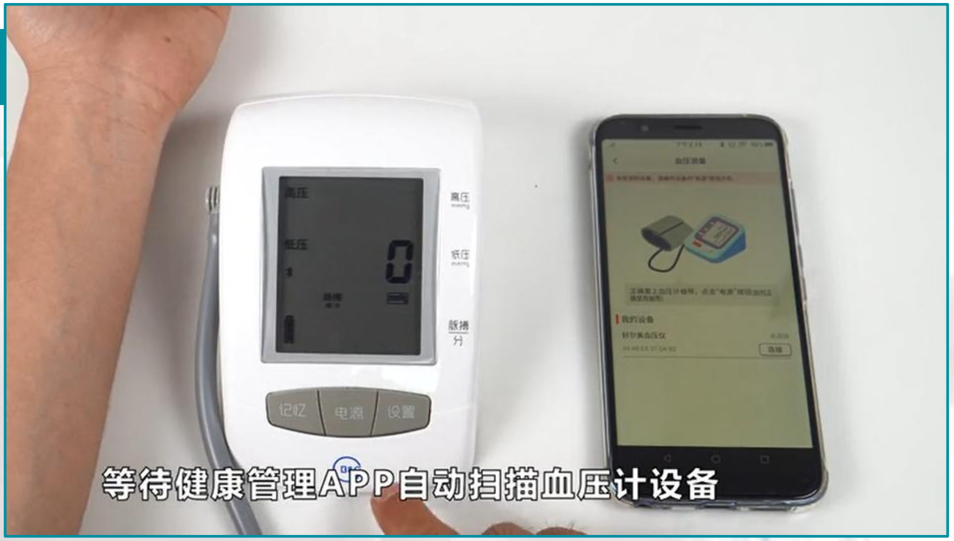

4

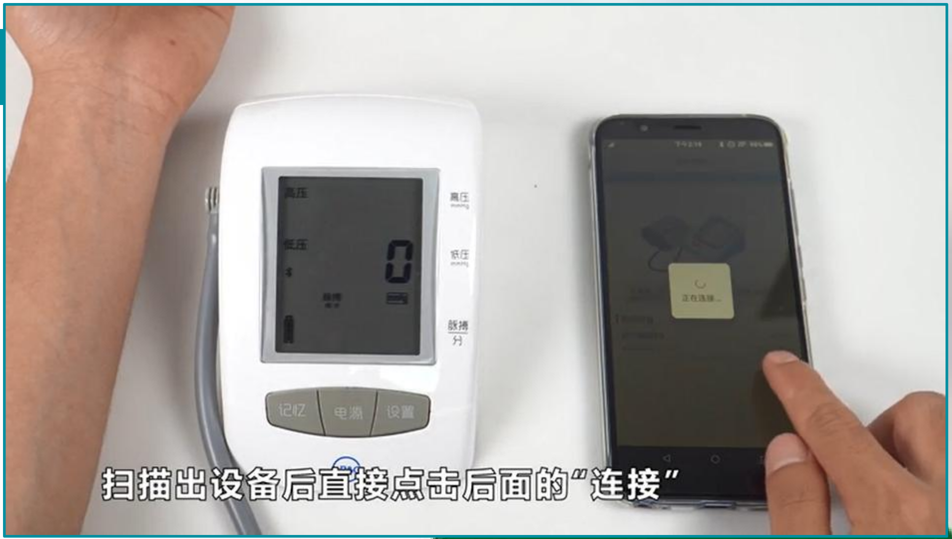

健康数据采集（血压测量规范）

5

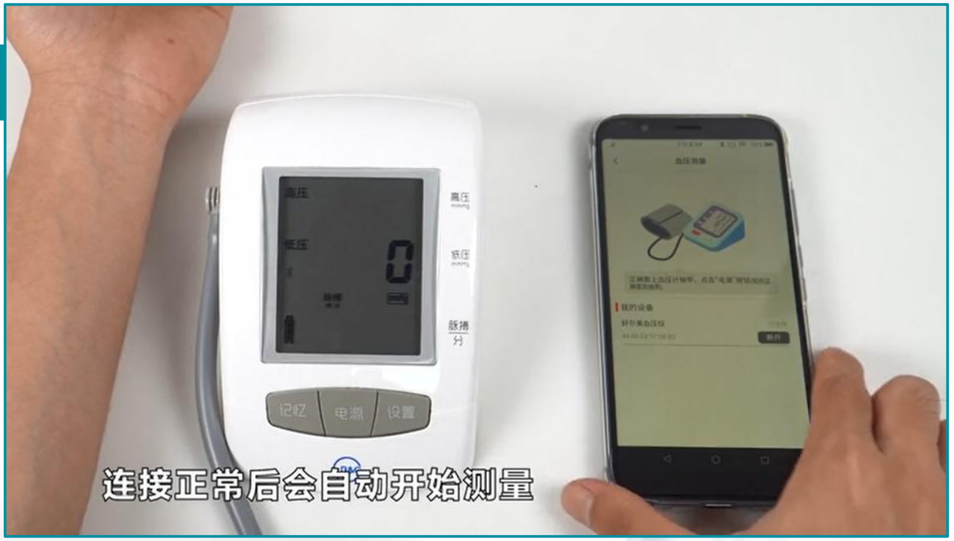

6

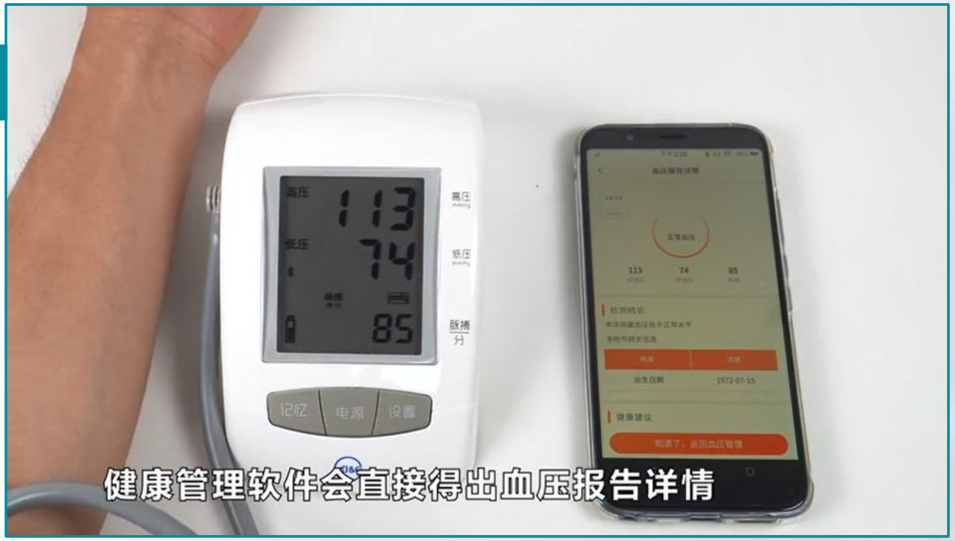

健康数据采集（血糖测量规范）

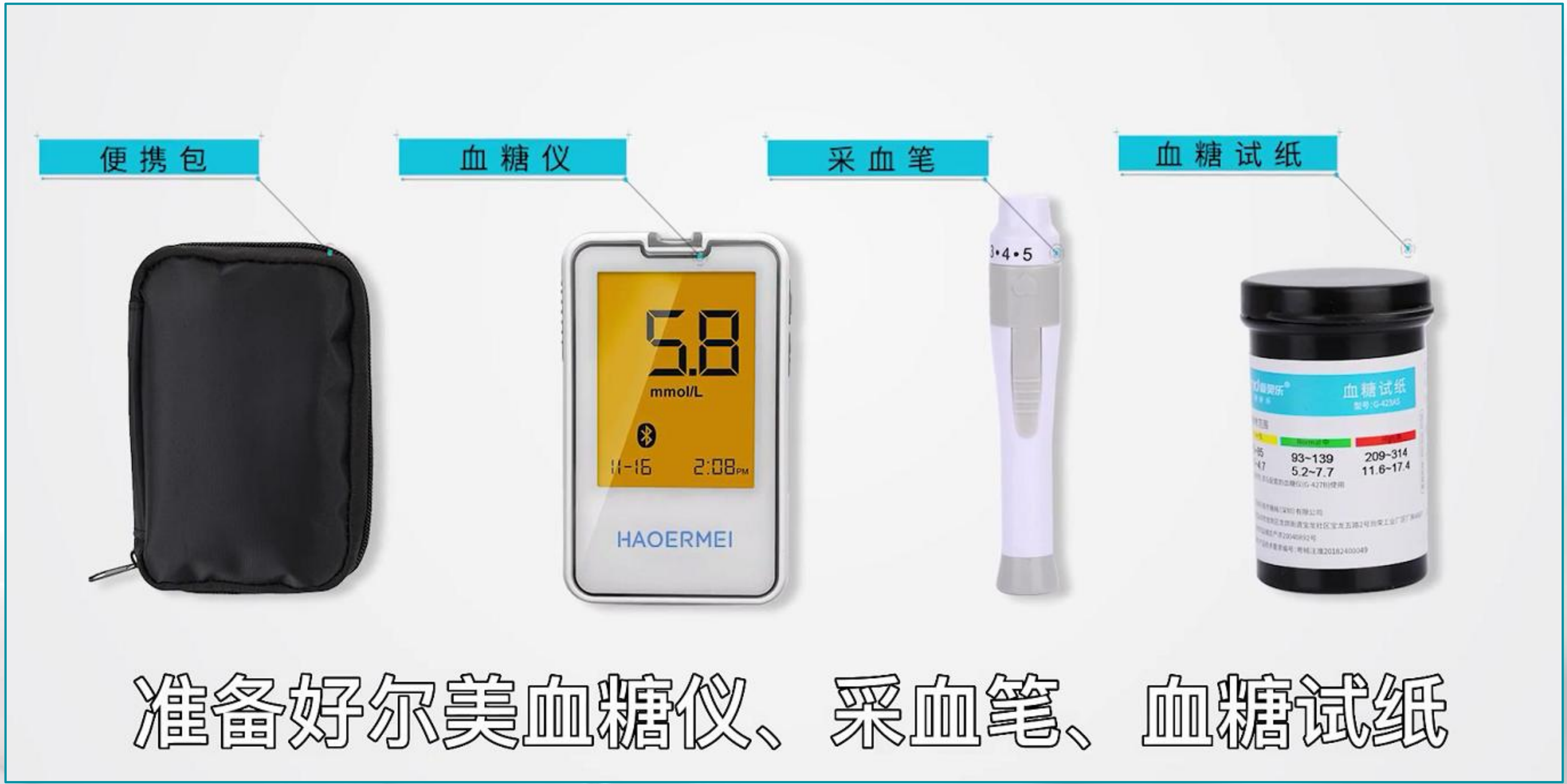

健康数据采集（血糖测量规范）

1

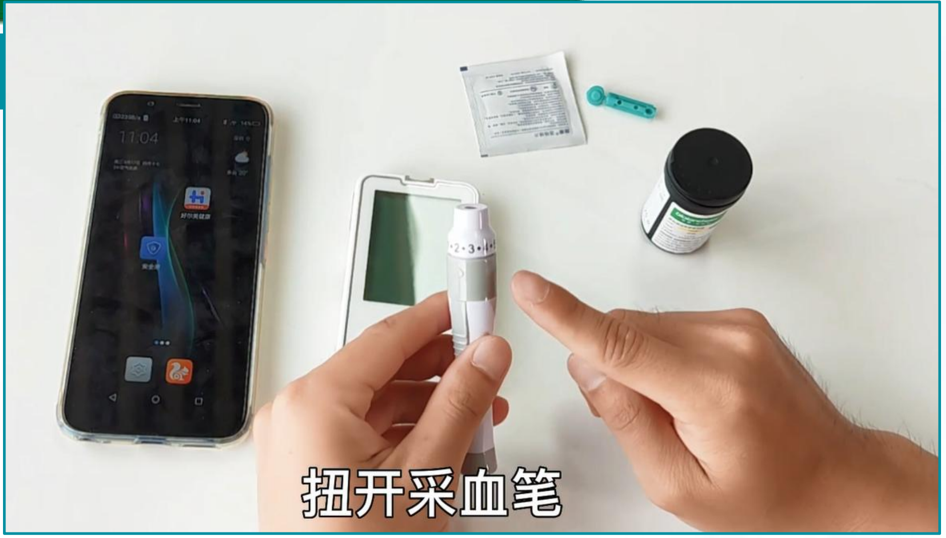

2

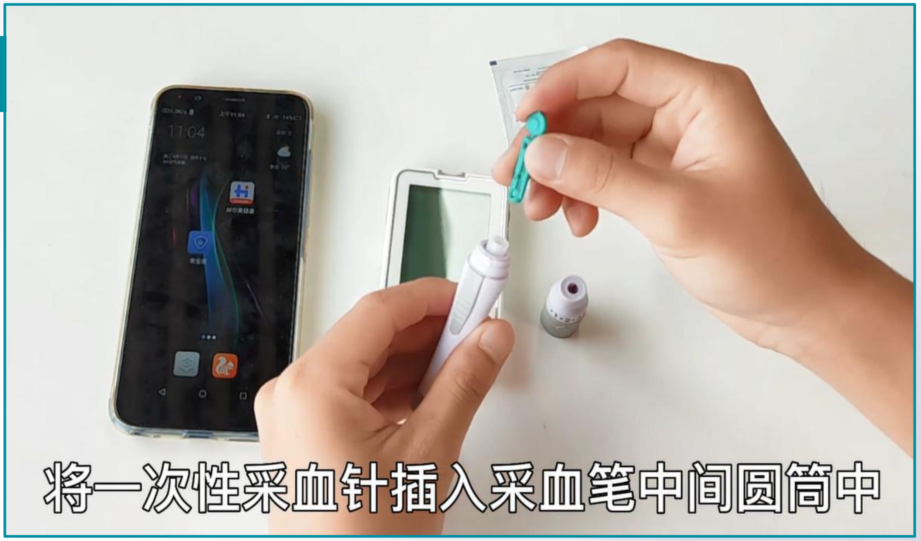

3

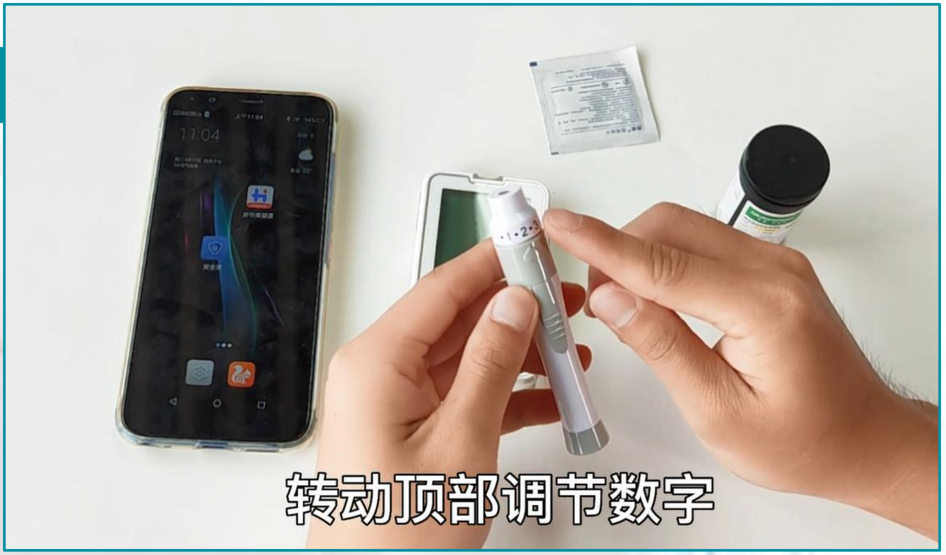

4

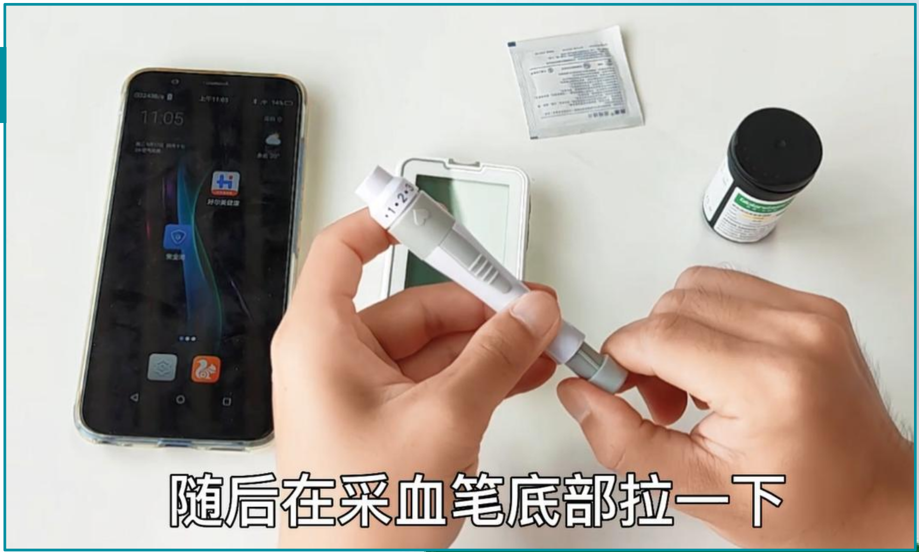

健康数据采集（血糖测量规范）

5

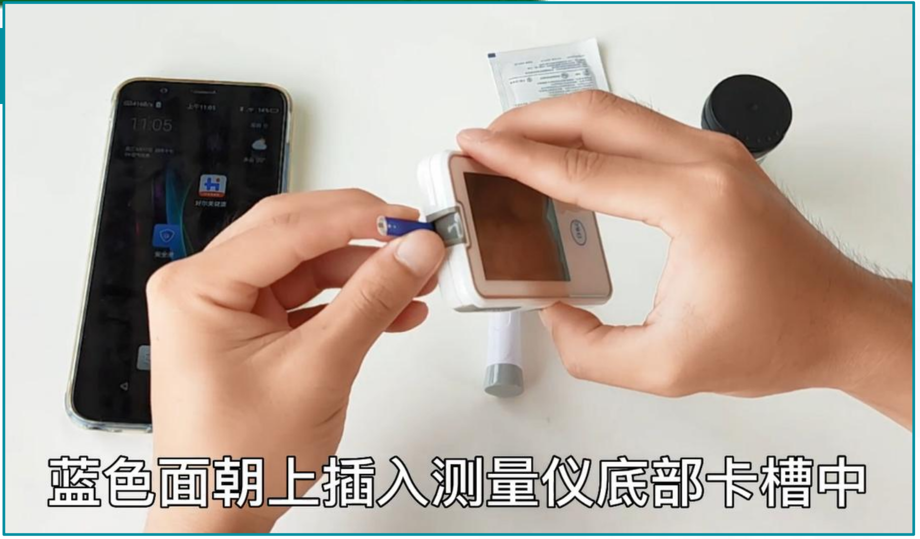

6

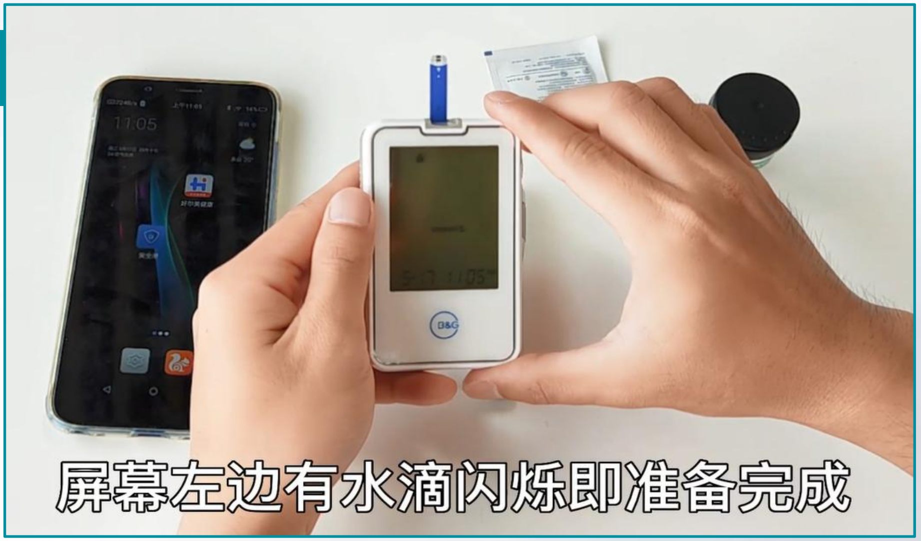

7

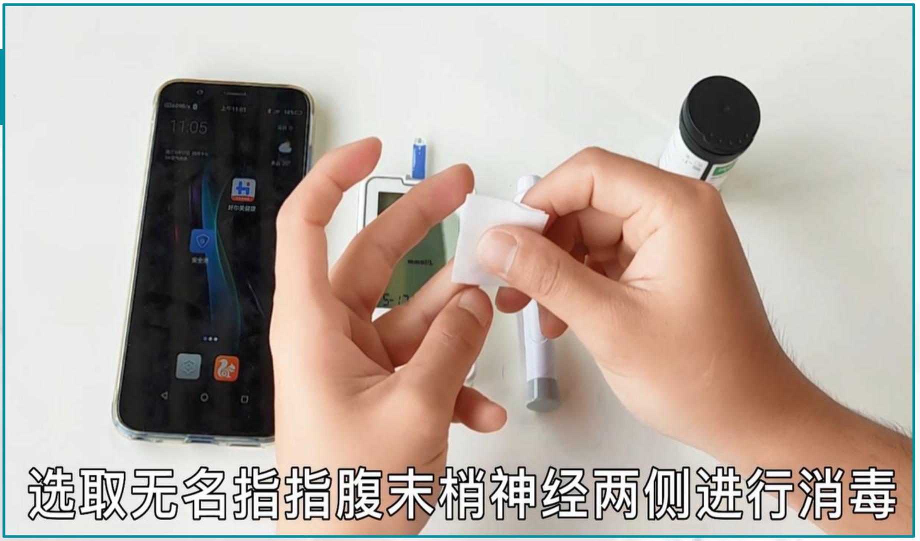

8

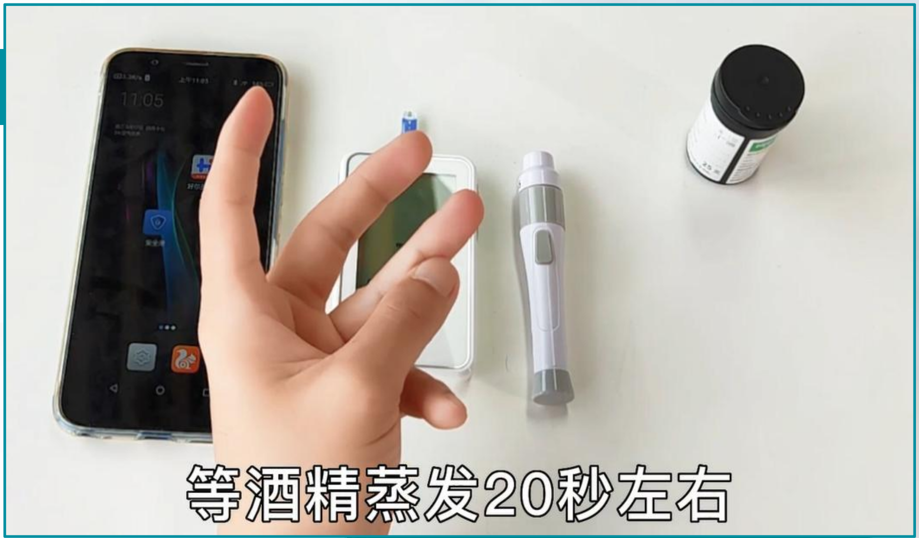

健康数据采集（血糖测量规范）

9

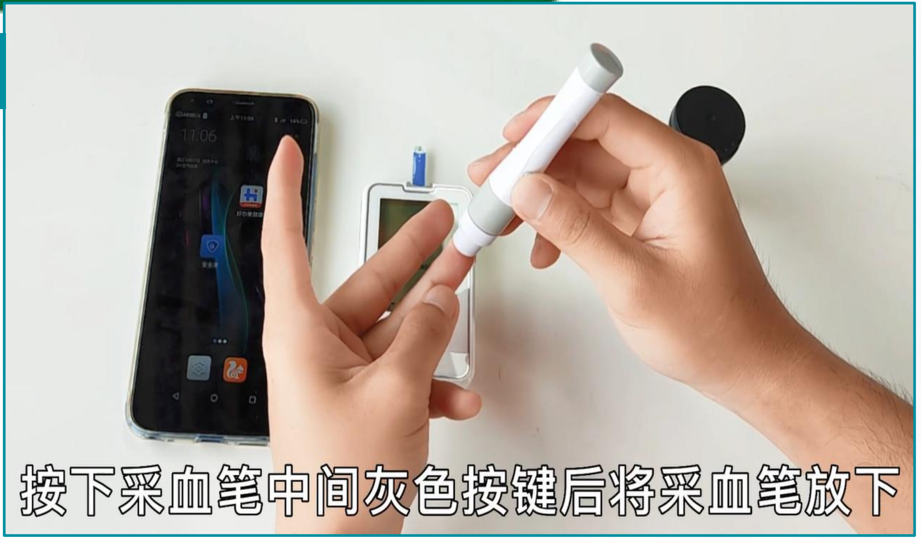

10

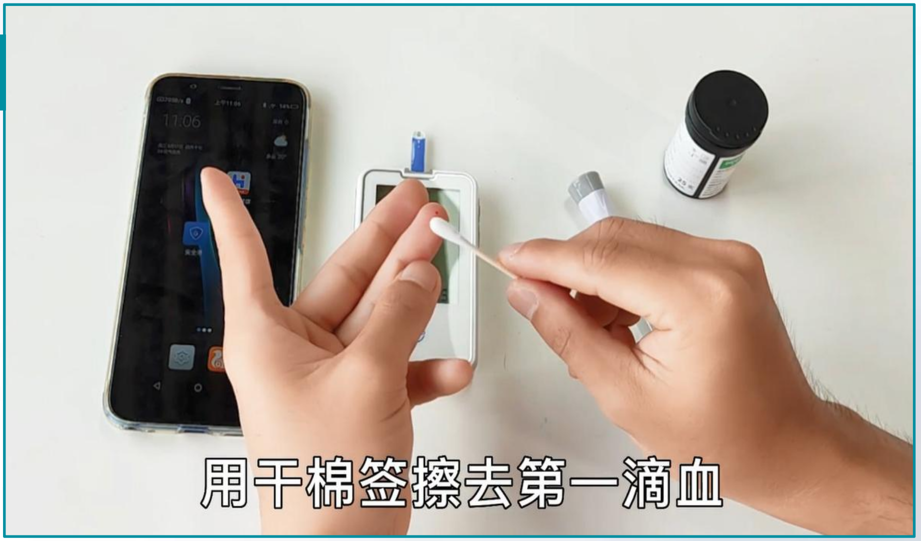

11

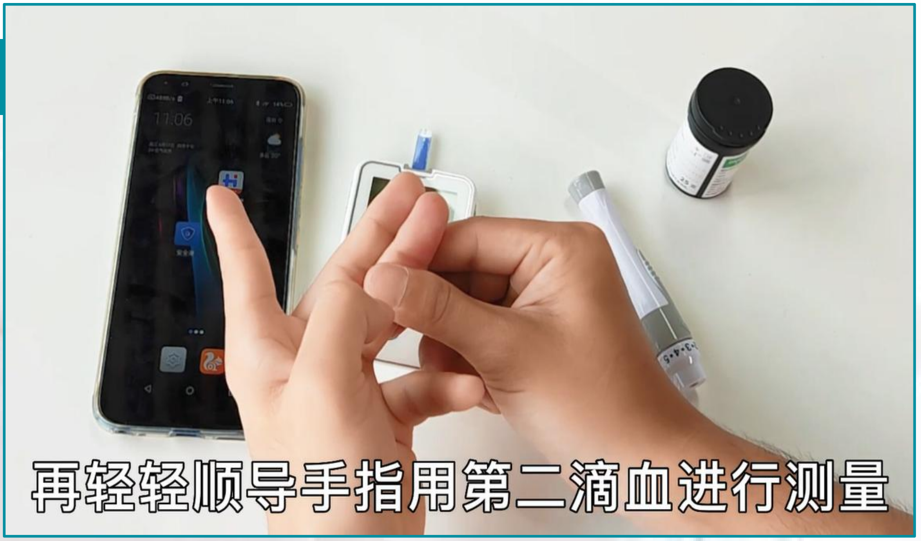

12

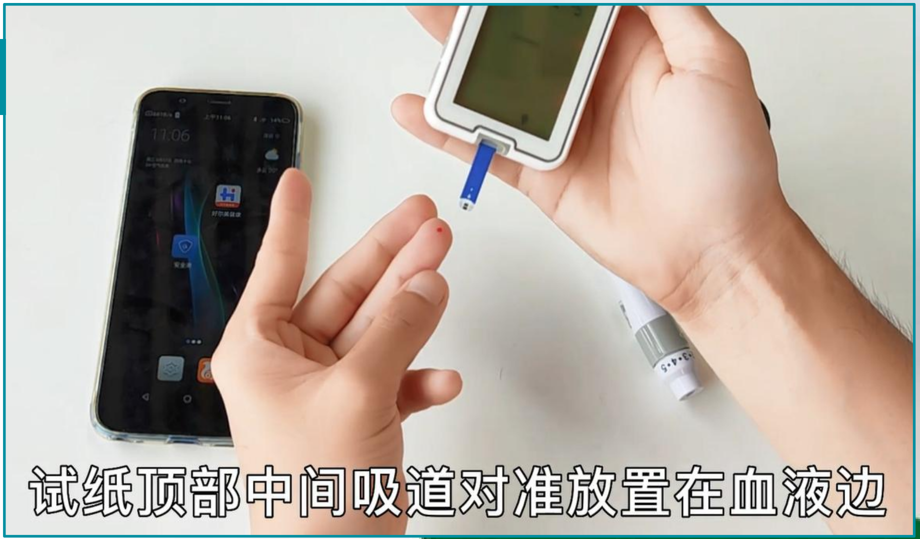

健康数据采集（血糖测量规范）

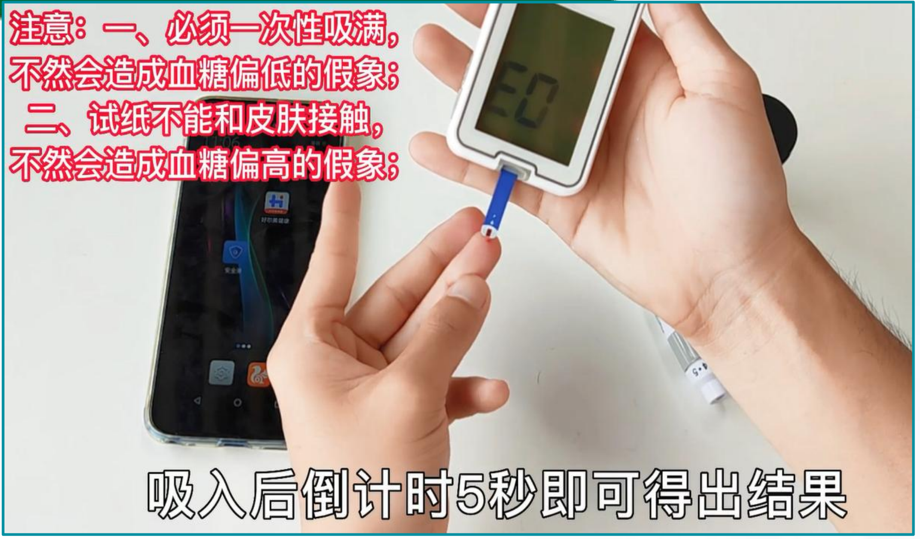

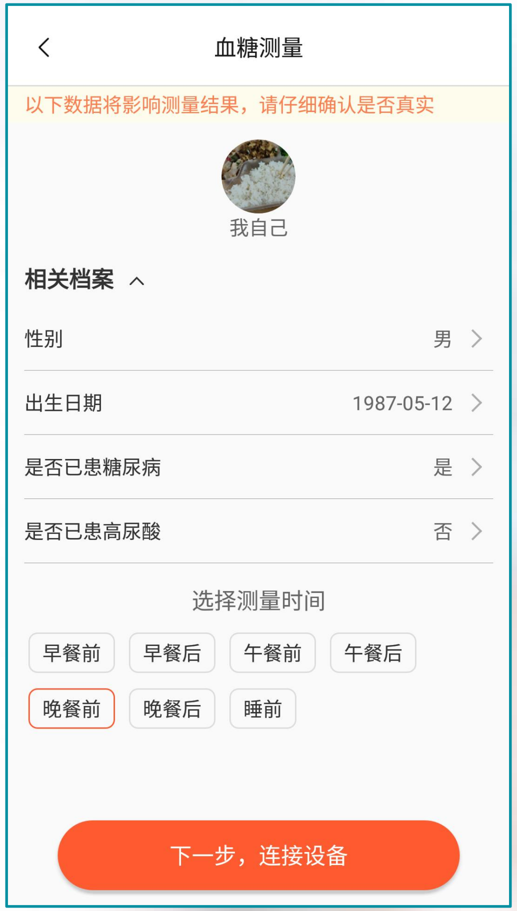

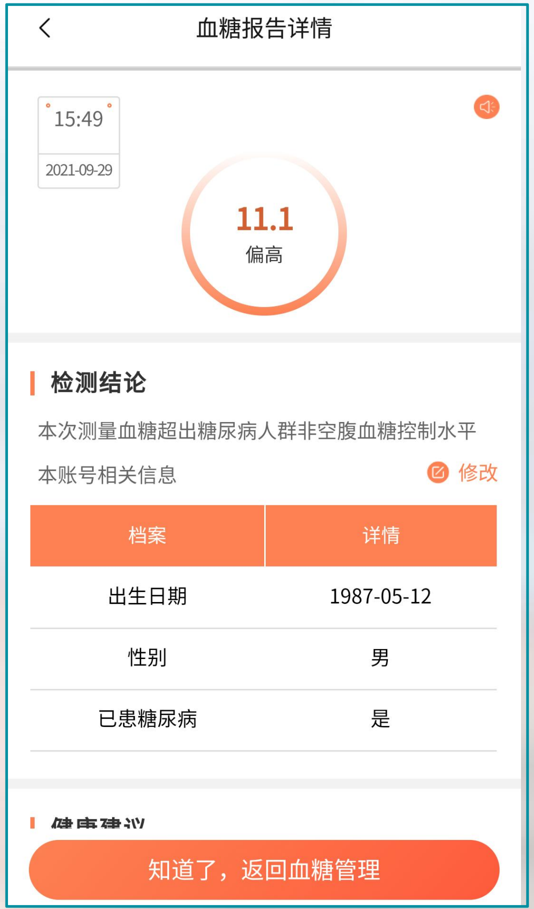

测量完成后要注意耗材处理：

1.采血针
2.酒精棉片
3.棉签
4.血糖试纸

南方医科大学坪山总医院
Pingshan general hospital of Southern Medical University

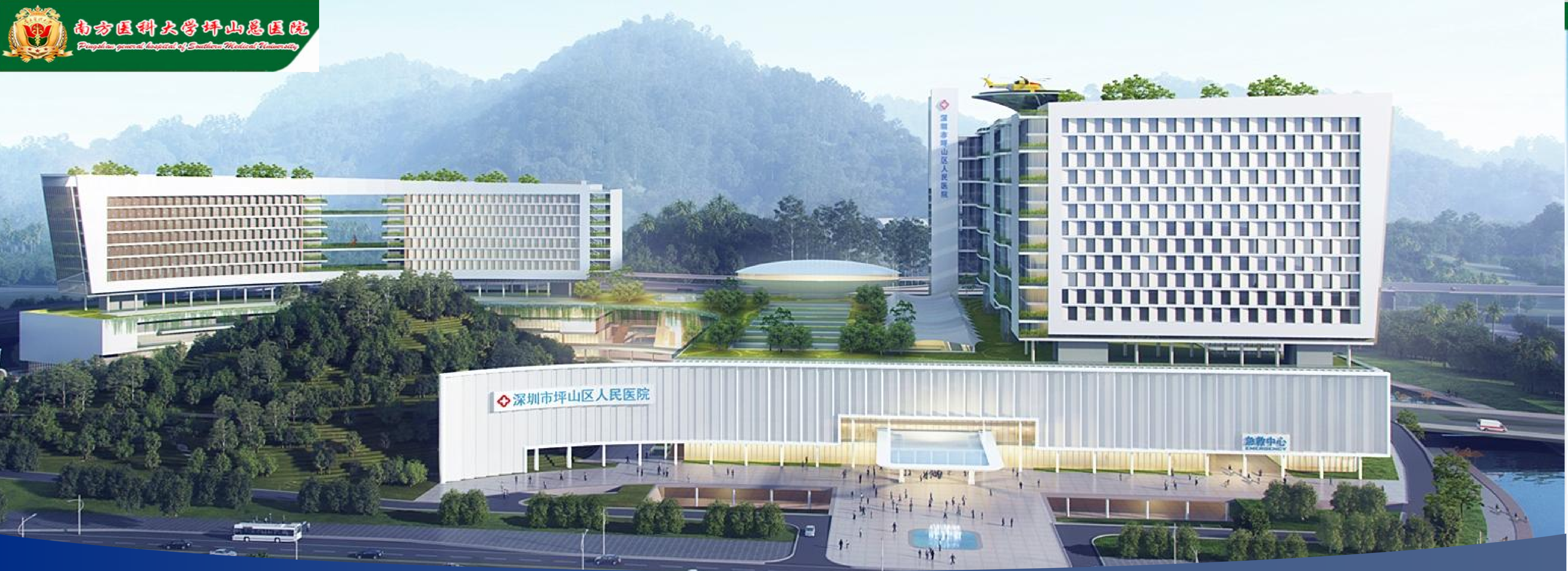

谢谢观看
