# Supplementary material for: Digital Health Platform for Improving the Effect of the Active Health Management of Chronic Diseases in the Community: Mixed Methods Exploratory Study
Source: J Med Internet Res. 2024 Nov 18;26:e50959. doi: 10.2196/50959 (PMC11612601; doi:10.2196/50959)
Supplement: Multimedia Appendix 3 [file jmir_v26i1e50959_app3.docx]

**居民主动健康指数**

**居民主动健康指数得分=1000-主动健康行为得分-健康管理结果**

**（一）主动健康行为**

| **序号** | **指标分类** | **指标名称** | **权重系数** | **变量赋值** |
| --- | --- | --- | --- | --- |
| 1 | 健康管理行为  **权重：50%** | 吸烟 | 8.6 | 0-10 |
| 2 |  | 饮酒 | 8.4 | 0-10 |
| 3 |  | 运动 | 8.4 | 0-10 |
| 4 |  | 饮食 | 8.6 | 0-10 |
| 5 |  | 睡眠 | 6 | 0-10 |
| 6 |  | 用药 | 8 | 0-10 |
| 7 |  | 体检 | 2 | 0-10 |

**（二）健康管理结果**

| **序号** | **指标分类** | **指标名称** | **权重系数** | **变量赋值** |
| --- | --- | --- | --- | --- |
| 8 | 健康管理效果**权重：50%** | 当前血压水平 | 6 | 0-10 |
| 9 |  | 当前血糖水平 | 6 | 0-10 |
| 10 |  | 体质指数BMI/腰围 | 4 | 0-10 |
| 11 |  | 甘油三脂 | 5.6 | 0-10 |
| 12 |  | 高密度脂蛋白胆固醇 | 4 | 0-10 |
| 13 |  | 低密度脂蛋白胆固醇 | 7.6 | 0-10 |
| 14 |  | 高尿酸血症 | 3 | 0-10 |
| 15 |  | 微量白蛋白尿 | 3 | 0-10 |
| 16 |  | 颈动脉斑块 | 8.2 | 0-10 |
| 17 |  | 心理疾患 | 2.6 | 0-10 |
