# Supplementary material for: Digital Health Platform for Improving the Effect of the Active Health Management of Chronic Diseases in the Community: Mixed Methods Exploratory Study
Source: J Med Internet Res. 2024 Nov 18;26:e50959. doi: 10.2196/50959 (PMC11612601; doi:10.2196/50959)
Supplement: Multimedia Appendix 4 [file jmir_v26i1e50959_app4.docx]

**Internet-based refined management of hypertension and diabetes operation manual**

**“互联网+”高血压糖尿病精细化管理操作手册**

**——基于互联网技术的社区高血压糖尿病“五师共管”模式**

**一、社区高血压糖尿病“互联网+五师共管”概述**

根据国家重点研发计划“健康管理应用示范区建设及应用示范效应评估”坪山示范区项目实施的需要，本课题组创建了主动健康信息平台，建立构建具有坪山特色的“五师共管”社区慢性病管理模式，制定五师共管中的“五师”的培养标准和操作流程。

本管理模式，结合社康中心和医院慢病患者或社区亚健康人群的健康信息，借助i主动健康小程序、智慧健康App和健康管理平台，实现全方位健康信息收集、健康风险评估和健康管理等服务，同时结合专业的“五师共管”团队（[家庭医生团队（医师+护师+药师）、健康管理师、营养师、心理咨询师、运动康复师]完成个性化健康干预，打造线上线下精准健康闭环服务体系，最终实现社区高血压、糖尿病患者个性化精准健康管理目标。其中，数字化精准健康管理依托相关健康管理APP和管理后台的相关功能落地实施，而线上线下个体化闭环健康管理由全科医生团队完成。

**二、干预对象与分组**

**1.研究现场：**

1.1试验街道：选取碧岭街道和石井街道为实验街道，在完成国家基本公共卫生服务项目高血压糖尿病健康管理的基础上，开展“五师共管”干预。

1.2对照街道：以坪山街道、马峦街道为对照组，采用传统管理。

1. **研究对象**

2.1试验组对象选取：以深圳市坪山区碧岭街道和石井街道的社区健康服务中心登记在册，并自愿参与本项目的社区高血压、糖尿病患者为试验组研究对象。其中，选取高血压患者、糖尿病患者各150名（共300名）发放可穿戴设备，开展

严格的“五师共管”模式管理，其余的在社康中心登记在册的高血压糖尿病患者（约2700人）不发放可穿戴设备，开展“五师共管”模式管理。

2.2发放设备患者标准

1. 在社康中心登记在册的确诊为高血压病、或/和2型糖尿病的患者；
2. 听力和意识良好、能听懂普通话；
3. 有智能手机，并懂得接收阅读微信的信息；
4. 近半年在本社康有过门诊行为；
5. 能完成测量次数要求与医生随访要求（每周至少2次测量数据上报，接受医生/医疗助理的电话或微信回访，之后根据病情进行照护和随访，能完成相关的健康打卡和线上学习课程）

（6）最终发放名单，由全科医生团队确定

2.3 排除标准

（1）听力和意识不良好、无法进行如常交流者。

（2）不能完成定期自测血压、血糖者。

（3）不接受随访，不参加本项目组织的相关线上或线下健康管理获得者。

2.4 对照组对象：

选取坪山街道、马峦街道所在社康中心登记再次的社区高血压、糖尿病患者为对照组研究对象。对照组采用常规管理。

**三、社康健康管理团队的建立**

1.1团队构成：根据社康中心慢病管理的需求，管辖5万人以上的区域社康中心建立一个到数个全科五师团队[家庭医生团队（医师+护师+药师）、健康管理师、营养师、心理咨询师、运动治疗师]参与本项目的高血压病和糖尿病患者管理。而规模较少的社康中心，每个团队最少有1名全科医生（团队长）、一名护士、一名健康管理师，而营养师、心理咨询师、运动治疗师可多家社康中心共用。

1.2团队长要求：具有获得医师资格3年以上，具有高血压和/或糖尿病管理的经验；具有参与本项目的热情，并能全程参与本项目的患者管理。

1.3“五师”的主要职责

| **“五师共管”各岗位职责明细** | |
| --- | --- |
| **分 类** | **主 要 内 容** |
| 家庭医生团队（医师+护师+药师） | 1. 制定和执行个体化诊疗方案、病情评估和必要时的转诊 2. 与患者签订家庭医生服务合约。 3. 对（高、糖）患者进行定期随访，带来团队完成国家基本公共卫生服务项目健康管理的内容。 4. 对（高、糖）患者进行周期性健康体检。 |
| 营养师 | 1. 负责营养膳食与健康调查并记录，了解患者的疾病史，对患者的饮食习惯和身体营养状况做出评估，制作营养膳食方案。 2. 为居民和患者制定个性化的饮食处方。 3. 对患者的饮食情况进行监督和指导，给出调理方案，并对其体重、BMI、血糖值等指标进行跟踪记录。 4. 通过线上开展患者和居民的营养健康教育和营养指导。 5. 通过线下讲座或电话咨询指导患者正确认识营养观念。 |
| 健康管理师 | 1. 根据病历、健康问卷、健康咨询、体检报告等收集到患者以往的健康信息，建立健康管理档案。 2. 制定个性化健康评估，出具健康干预方案。 3. 通过线上，线下管理患者的日常随访与健康教育，指导患者实现自我健康管理。 4. 向全科医师反馈患者的病情变化，安排患者的随诊时间及全科-专科，社区-医院间双向转诊的相关事宜。 |
| 运动康复师 | 1. 负责患者身体指标动态监测、评估、方案制定与跟踪。 2. 为居民和患者制定个性化的运动处方。 3. 负责患者运动功能评定，包含肌力、关节运动范围(ROM)、平衡能力、体位转移能力、步行能力及步态的评定，并制定和执行体疗方案。 4. 负责康复训练全过程的安全把控、数据监测与管理。 5. 开展慢病生活方式健康教育活动（营养、运动、训练、用药等）。 |
| 心理咨询师 | 1. 依据设备仪器等相关检测数据，对患者的心理人格发展、智力、社会化及生活事件等进行全面心理评估。 2. 通过系统的心理咨询，帮助患者走出心理困惑，收获心灵成长和人格健康。 3. 对患者的心理健康情况进行定期监测与记录。 4. 通过线上、电话等途径给与患者心理咨询和心理辅导。 5. 开展线下心理咨询 6. 组织心理健康相关的健康讲座和活动。 |

**四、“五师共管”健康干预实施**

**（一）干预重点**

**1. Ⅱ型糖尿病的健康干预重点工作**

1.1掌握Ⅱ型糖尿病诊断标准、非药物干预内容、药物治疗路径、健康管理控制目标，开展30岁以上居民测血糖活动，社区和医院通过赠送居家血糖监测设备，在患者、预后康复者、高危人群筛查血糖，居民健康档案建立过程中询问等方式发现糖尿病患者或糖尿病前驱期人群，安装主动健康管理APP，建立慢病健康档案。

1.2掌握Ⅱ型糖尿病患者健康管理服务对象、随访管理评估内容、分类干预要求和健康体检项目，明确日常饮食、运动随访服务记录表单规范填写方法。

1.3熟悉Ⅱ型糖尿病筛查途径、精准健康管理服务流程和指标定义；熟悉Ⅱ型糖尿病高危人群判定标准和”五师共管“健康干预要求，对患者和前驱期人群每月提供2次面对面的随访（包括线上随访）。

1.4糖尿病患者每半年至少进行一次全面的健康检查，可与随访相结合。内容包括血压、血糖、血脂、体重及体格、视力、听力、活动能力等一般检查，并将检查结果填入APP；高血糖或糖尿病前驱期群体，发现血糖异常的第一个月，每天至少监测两次血糖值，如没有居家检测设备，需要到社区设立的主动健康项目体验点进行免费检测。

**2.高血压的健康干预重点工作**

2.1通过广泛宣传和教育，开展35岁以上居民测血压活动，社区和医院通过赠送居家血压监测设备，在患者、预后康复者、高危人群筛查高血压，居民健康档案建立过程中询问等方式发现高血压患者，并安装主动健康管理APP，建立慢病健康档案。

2.2对确诊的高血压患者，每月提供2次面对面的随访（包括线上随访）。每次随访全科医生团队要询问病情，进行血压测量和评估，对用药安全进行咨询、”五师共管“团队要对饮食、运动、心理等进行健康指导，并通过APP记录。

2.3高血压患者每年至少进行一次全面的健康检查，可与随访相结合。内容包括血压、血糖、血脂、体重及体格、视力、听力、活动能力等一般检查，并将检查结果填入APP；高血压或疑似高血压群体，发现血压异常的第一个月，每天至少监测1次血压，如没有居家检测设备，需要到社区设立的主动健康项目体验点进行免费检测。

1. **发放可穿戴设备患者的干预流程**

1.开展全方位的宣传：各社康中心利用机构内宣传栏、宣传海报、电子显示屏、横幅等形式，加大宣传推广本项目有关知识，提高本项目的知晓率，鼓励患者积极参与，创造良好舆论环境。

2.邀请患者入组，签订设备使用协议，发放设备：经确诊入组的高血压糖尿病患者持身份证到发放机构领取，工作人员负责讲解血压计、血糖仪、智能手环的相关操作、注意事项；患者签署设备使用协议后领取设备、试纸配件和用户手册，拉患者进入健康管理微信群。


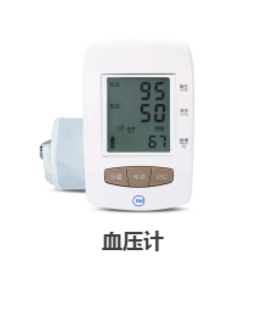

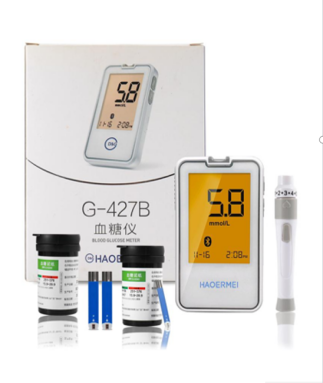

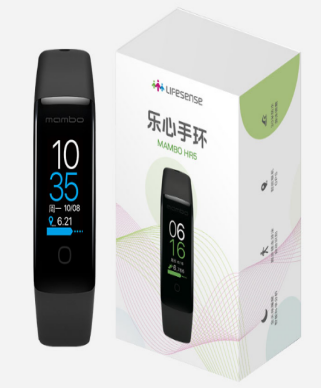

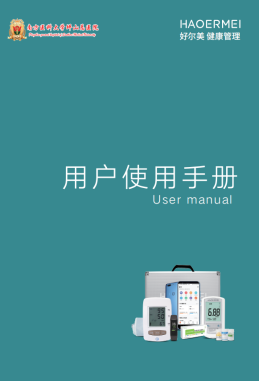


3.信息录入：协助患者注册 i主动健康小程序、或下载“坪山医健”APP，并进行注册、核对和录入患者信息（前期本项目组已经把相关患者的数据导入小程序和APP中）。


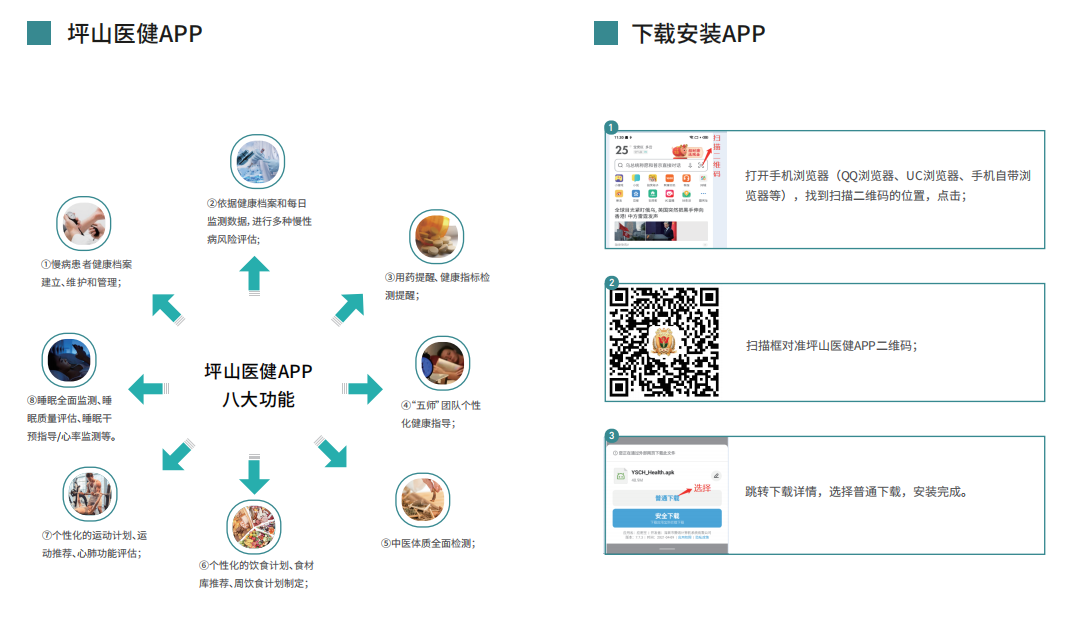


4.完成患者入组快速测评量表、主动健康素养评估问卷。

5.现场教会患者使用血压计、血糖仪、智能手环的使用，培训患者使用 i主动健康小程序、“坪山医健”APP，并需要完成的健康打卡、线上课程学习等。


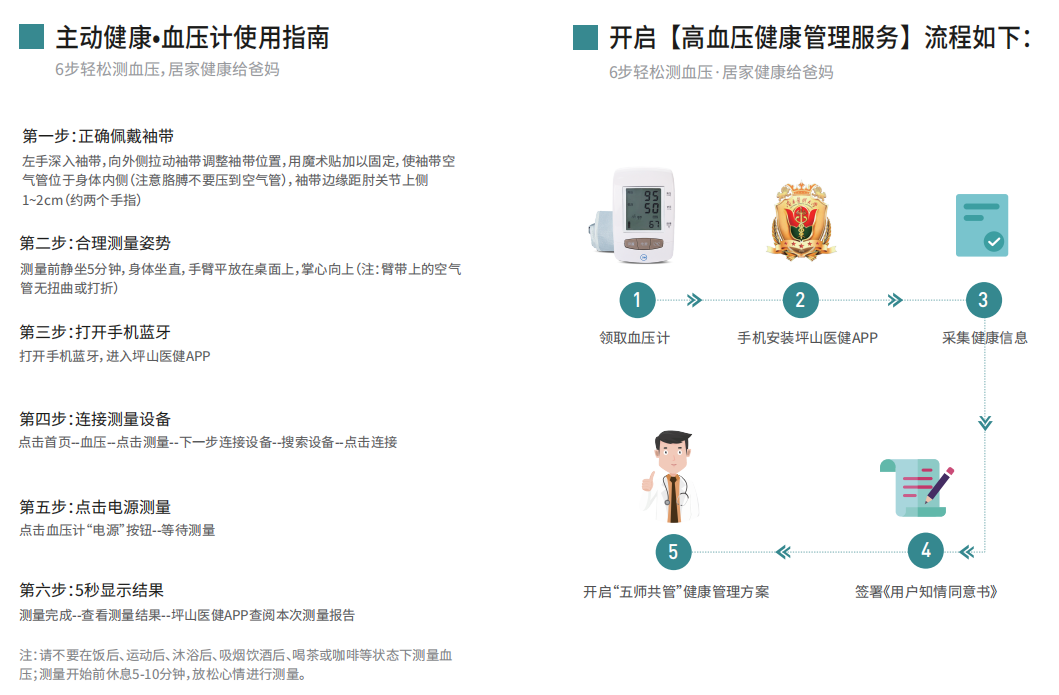

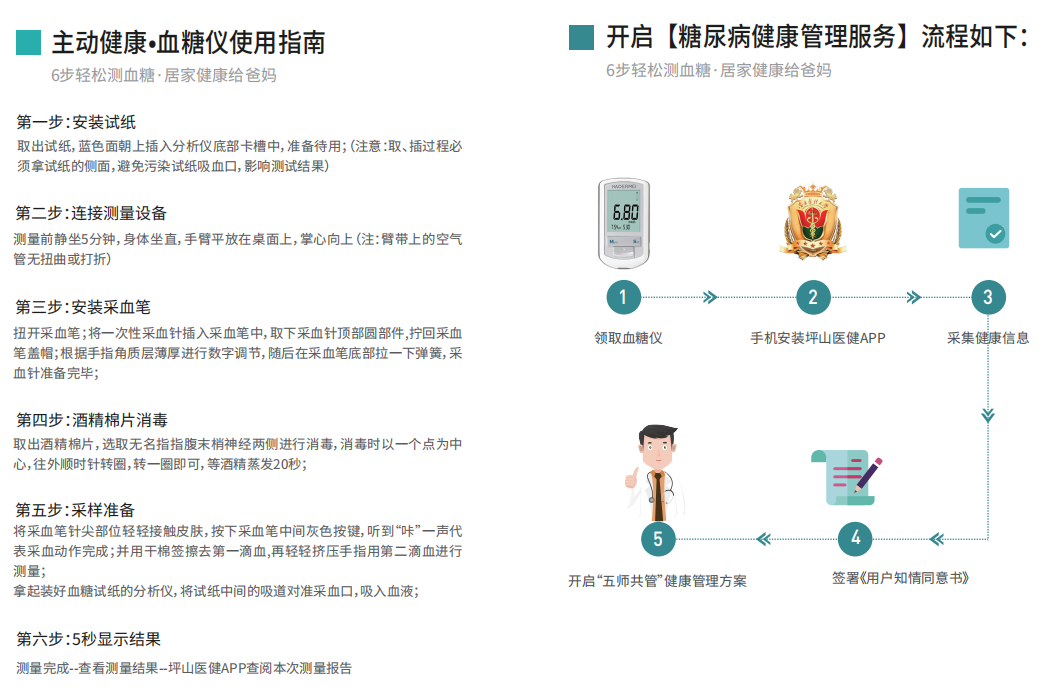


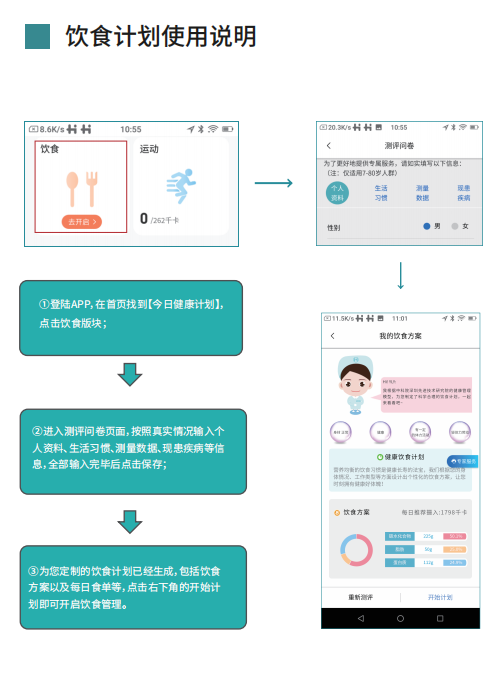

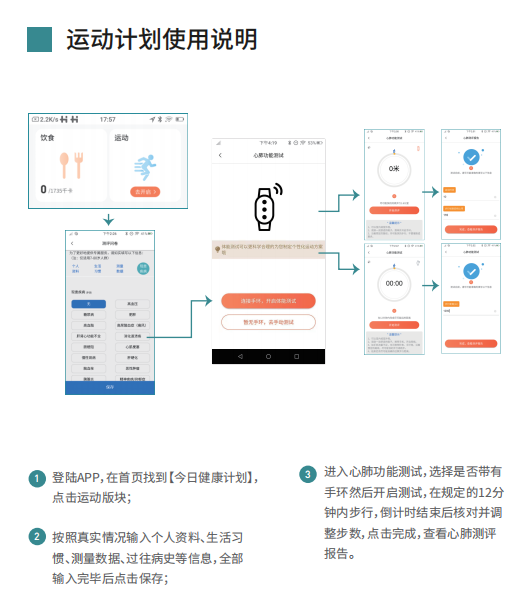


**
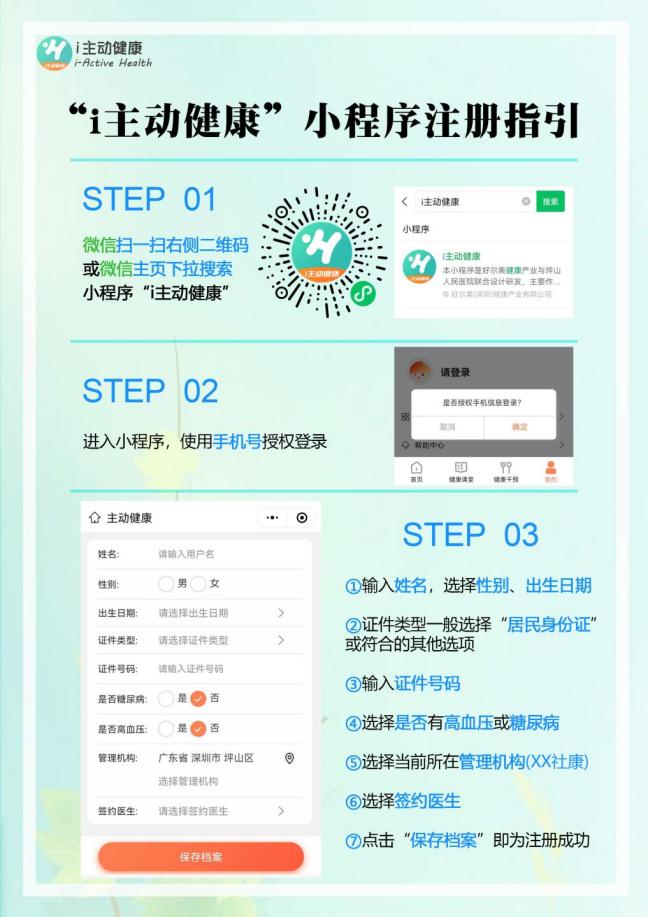
**

**6.开展“互联网+五师共管”健康干预**

**患者入组流程**

1.1开展全方位的宣传：各社康中心利用机构内宣传栏、宣传海报、电子显示屏、横幅等形式，加大宣传推广本项目有关知识，提高本项目的知晓率，鼓励患者积极参与，创造良好舆论环境。

1.2邀请患者入组，签订设备使用协议，发放设备：经确诊入组的高血压糖尿病患者持身份证到发放机构领取，工作人员负责讲解血压计、血糖仪、智能手环的相关操作、注意事项；患者签署设备使用协议后领取设备、试纸配件和用户手册，拉患者进入健康管理微信群。

1.3信息录入：在“i主动健康小程序”或“坪山医健”APP中注册、核对和录入患者信息。

1.4完成患者入组快速测评量表、主动健康素养评估问卷。

1.5现场教会患者使用血压计、血糖仪、智能手环的使用，培训患者使用“i主动健康小程序”或“坪山医健”APP、健康打卡、线上课程学习等。

**（3）开展“互联网+五师共管”健康干预流程**

①患者通过血压计、血糖仪检测终端自测血压/血糖，数据将自动传输。

②患者本人及家属、五师团队同时获得患者的检测结果。

③主动健康管理信息平台对患者智能分组、量化测评和管理。

④“i主动健康小程序”和“坪山医健”APP为患者和家属提供形式多样的、融学习与娱乐于一体的健康教育活动。

⑤患者家属督促糖尿病患者在家的服药、饮食、运动干预情况。

⑥五师团队通过微信群、电话和现场会等方式对患者及家属进行随访、进行健康教育（包括用药，饮食，运动，心理辅导、合理作息的健康教育），并将患者的状况及时反馈给全科医生。

⑦全科医生实时动态掌控患者的病情，对需要服务的患者进行有针对性的诊治和健康管理，对于血压、血糖控制不良的患者，转诊到专科的专家就诊。

⑧邀请专家每月举办一场慢性病健康管理讲座，并定期举办患者和家属交流会了解管理进展。（见图1）。


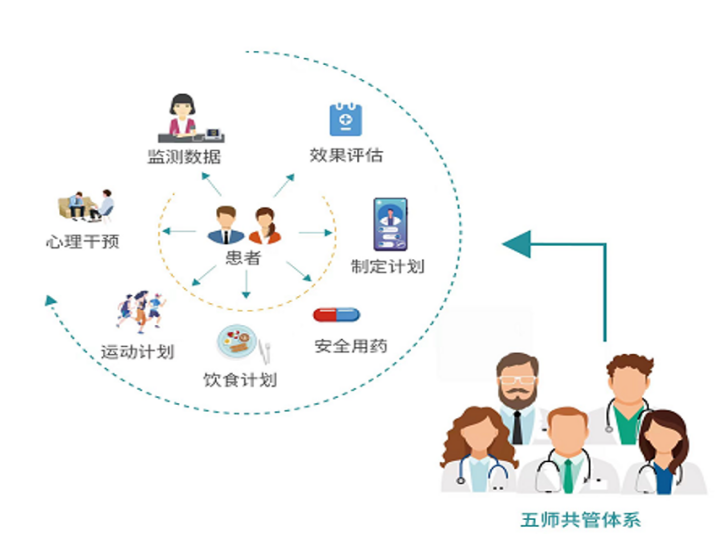


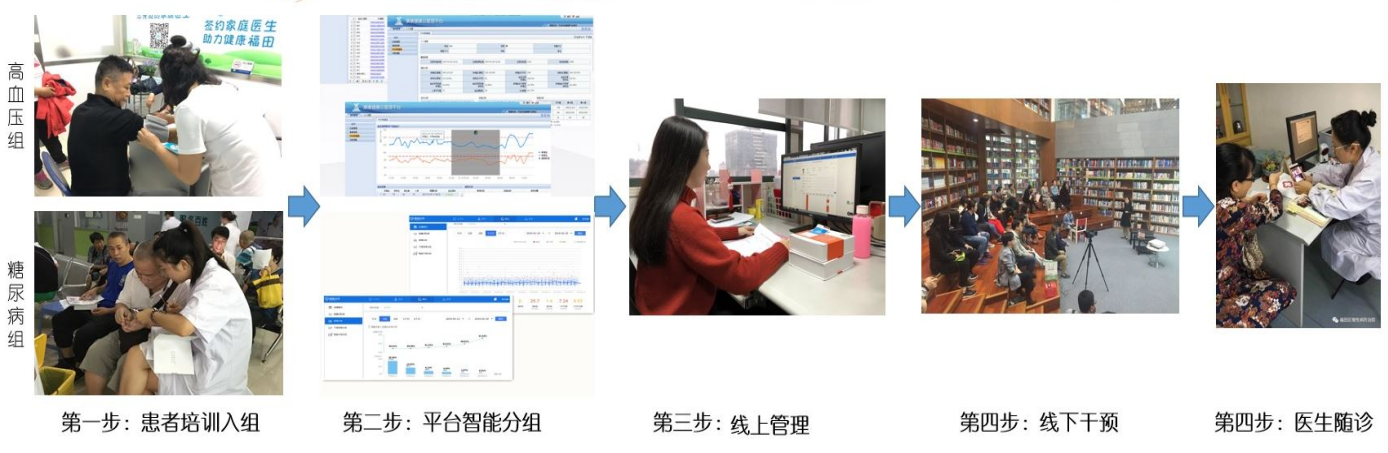


**图1.“互联网+”五师共管社区慢性病新模式示意图**

1. **不同干预期的要求：**

本管理模式，分强化管理期（1个月）、巩固期（3个月）和持续干预期三个阶段。由五师团队分工配合完成，并根据患者的需要请心血管科医生、内分泌科医生专科医师予以诊治。

**1.1强化管理期（1个月）**

①管理对象：慢性病（高血压、糖尿病）患者

②随访次数：每周两次

③随访方式：电话、线上随访、面访，线上记录随访和干预工作。

**第一周（入组诱导）**

①分工：主要由健康管理师或社区护士完成患者的随访和指导。

②本周共随访3次，包括入组第二天的电话随访，后续电话或微信随访2次。

③每天观察患者的数据，并在患者群中发送慢病管理相关知识的短信，回答患者提出的问题。

④患者入组第二天，在小程序或APP上查阅患者相关信息，完成首次电话回访：包括了解患者对“主动健康”检测设备（血压计、血糖仪、运动手环等）的使用情况，嘱其坚持每日检测血压/血糖一至两次，坚持佩带运动手环，并按照原来运动习惯；

⑤教会患者记录膳食打卡，并嘱咐患者保持原有的饮食习惯；

⑥建立小组线上群聊，补充教学，更好地引导患者；

⑦第二、三次随访，均要询问患者设备使用情况、血压和（或）血糖变化情况、用药情况（如有服药）、饮食、运动和睡眠情况，并反馈患者饮食、运动和睡眠的不合理之处，并给出改善建议。

**第二、三周（强化干预）**

①分工：家庭医师团队：对血糖血压控制不良的患者调整生活行为和药物行为。五师团队：完成患者的随访和健康行为指导。

②健康管理师或社区护士每周随访2次，电话或微信均可，记录干预工作。

③每天观察患者的数据，并在患者群中发送慢病管理相关知识的短信，回答患者提出的问题。

④随访中指导患者制定个性化饮食处方和运动处方，制定阶段目标，指导患者按照处方执行，要求患者每天进行饮食打卡。为患者确定相对固定的运动量及有效运动量，完成规范运动的阶段目标。指导患者应用食物交换份丰富食物品种和烹饪技巧，并注重特殊疾病的饮食调配和注意事项。

⑤要求患者坚持检测血压/血糖每周两次以上，坚持佩带运动手环。

⑥全科医生根据患者血压和（或）血糖的分级情况、药物的使用、个人的依从性，对其进行分组管理并给予干预：

**第四周（强化小结）**

①分工、随访频次、随访内容与2-3周相同。

②分析患者对饮食处方和运动处方的完成情况，做理化指标、运动量变化趋势、三大营养素变化趋势、三餐比例变化趋势，分析是否实现管理的阶段目标，进一步规范运动与饮食行为。

③全科医生分析患者的血压、血糖控制情况、用药情况，根据患者情况可适当调整其使用药物。

**1.2巩固管理期（3个月）**

①巩固期从入组第2个月开始到第4月结束。

②随访频率：每周一次，健康管理师或社区护士采用电话或微信随访均可，线上记录随访和干预工作。

③门诊复诊：1次以上。

④健康管理师在信息系统中每天观察患者的数据，并在患者群中发送慢病管理相关知识的短信，回答患者提出的问题。五师团队分工开展诊疗、营养、运动、心理干预。

⑤随访和线上指导内容：患者对饮食处方和运动处方的完成情况，相对固定患者的运动行为和饮食习惯；检查、巩固各项干预措施的落实情况，建立起健康的生活方式。

⑥门诊复诊内容：完成国家基本公共卫生服务项目要求的随访和体格检测内容，详细了解患者对饮食处方和运动处方的完成情况。对血糖血压控制不良的患者，分析主要原因，给与健康指导或调整药物，对于随访两次依然未由患者者，转专科医生处诊治。

⑦填写主动健康评估问卷，做阶段性评估。

**1.3持续干预期**

①持续干预期从入组第5个月开始到项目结束。

②随访频率：健康管理师或社区护士每周一次，采用电话或微信随访均可，线上记录随访和干预工作。五师团队分工开展诊疗、营养、运动、心理干预。

③门诊复诊：每3个月随访1次以上。

④每天观察患者的数据，并在患者群中发送慢病管理相关知识的短信，回答患者提出的问题。

⑤随访、门诊复诊的内容、干预方法同巩固管理期。

⑥填写主动健康评估问卷，做年度管理效果评估。

（三）不发放可穿戴设备的高血压、糖尿病患者的干预要求

（1）对登记在册每位患者，完成每季度一次以上的面对面随访：按要求完成诊疗、随访表的问询、体查和健康指导。

（2）每次来社康中心随访前一天，发送短信或电话提醒患者。

（3）在课题组研究生的协助下，在诊前筛查开展患者的风险评价和主动健康课题相关问卷测评（逐步开展包括主动健康素养、社会支持、家庭功能、自我效能、心理健康问卷测评等）。

（4）协助患者注册小程序或下载坪山医健APP，用于后续的主动健康管理。

（5）建立社区慢病患者微信群，全科医生团队每天推送一条健康教育信息，并安排团队成员回复患者在群里提出的问题。

（6）每月开展主动健康和健康管理的讲座（内容包括慢病的自我管理、用药、运动、饮食处方等）。

（7）待疫情允许时，组织患者线下活动（患者交流会、集体活动、知识竞赛、技能培训等，自我管理小组活动）

**五、“互联网+五师共管”健康管理培训**

**（一）针对医护团队，“五师共管”健康管理服务的培训内容**

（1）五师共管“健康管理服务流程和规范培训：营养指导、运动行为、心理干预等操作规范和业务流程；

（2）“五师共管”健康管理服务项目培训包括：营养、运动、健康管理、心理干预等专业，及其干预方法、流程、规范动作；

（3）“五师共管”健康管理重点健康管理项目培训包括：“六高”健康管理方案（高血糖、高血压、高血脂、高尿酸、高体重、高同型半胱氨酸）

（4）“五师共管”健康管理重点项目和个性化健康管理项目；

**六、社区高血压病患者主动健康监测和效果评估**

1.定性方法：通过访谈、督导、检查等方式，了解社康中心在患者管理中存在的问题、困难和建议，为进一步完善项目工作提供参考。

2.定量方法：通过问卷调查、体格检查、临床辅助检查、实验室检测和数据分析。分析患者的主动健康素养、主动检测、主动干预行为、自我效能、生活质量的改善情况；患者血压、血糖、血脂、体重和尿微量白蛋白等指标水平。

3.依从性生活质量评价：通过依从性问卷、心理卫生相关量表和生活质量表等工具评价实验组和对照组患者在干预前后卫生服务依从性、心理健康和生活质量变化情况。

4.卫生经济学评价：对“互联网+五师共管””慢性病健康管理模式进行成本效益和成本效果分析。

**社区高血压病患者主动健康监测和效果评估内容**

**说明：**社区高血压人群的检测指标主要是“居民主动健康素养”+国家基本公共卫生项目中关于高血压健康管理的指标，及其相关体检结果的动态变化结果。

|  | 数据获取方法 | 备注 |
| --- | --- | --- |
| **一、主动学习情况 界定主动被动**  经常阅读健康相关书籍、公众号、网页情况（每周4天以上）  （是、否）  观看或收听健康讲座或视频 （每周1次以上）  （是、否）  有健康相关问题，主动咨询医务人员  （是、否） | 主动健康素养问卷调查 |  |
| **二、高血压相关知识掌握情况**  高血压基本知识掌握情况  （完全掌握、部分掌握、不掌握）  基本的营养管理知识掌握情况  （完全掌握、部分掌握、不掌握）  基本的运动管理知识掌握情况  （完全掌握、部分掌握、不掌握）  降压药物知识掌握情况  （完全掌握、部分掌握、不掌握） | 主动健康素养问卷调查 |  |
| **三、主动健康监测情况**  定期自我监测血压频率  （每天监测血压、每周监测血压、每月监测血压、每半年监测血压、每年监测血压、很少测血压、从来没有测血压）  定期体检情况  （每年体检、2年体检一次、三年以上体检一次）  定期自我监测体重  （每天称体重、每周称体重、每月称体重、每半年称体重、每年称体重、很少称体重）  定期自我监测血糖  （每天监测血糖、每周监测血糖、每月监测血糖、每半年监测血糖、每年监测血糖、很少测血糖、从来没有测血糖）  定期做心理健康测评情况  （每天做心理健康测评、每周做心理健康测评、每月做心理健康测评、每半年做心理健康测评、每年做心理健康测评、很少做心理健康测评、从来没有做过心理健康测评）  配置了可穿戴设备患者的数据活度（如有） | 主动健康素养问卷调查  主动健康信息平台 |  |
| **四、生活行为情况**  睡眠（平均每天睡眠时间）  睡眠时是否打鼾 （是、否）  饮食口味习惯  （无特别、喜欢偏咸、喜欢偏淡、喜欢偏甜、喜欢偏辣、喜欢油炸）  饮酒的频率  （从来不喝酒、偶尔礼节性喝酒、每月喝酒、每周喝酒≥1次、每周喝酒≥3次、每天喝酒  日饮酒量（平均 两）  饮酒种类  （白酒、啤酒、红酒、黄酒、其他)  抽烟状况（日吸烟量）  运动（运动锻炼的频率、每次锻炼的时间、锻炼的方式）  平时参加社区、单位或学校组织的健康相关活动情况。  （从不参加、偶尔参加、经常积极参加） | 主动健康素养问卷调查、  社康信息系统  主动健康信息平台 |  |
| **五、主动健康干预情况**  就医依从性（当您与社康中心/医院有约定就诊时间，您是否都按时就诊？ 是、偶尔不按时、都不按时  用药依从性情况（当您要使用降压药时）  （规律、间断、不服药、无需服药）  针对健康行为改变情况  （控盐情况、饮食行为的改善、运动行为的改善、心理状况的调整、工作岗位的调整、其他调整，无作任何改变） | 主动健康素养问卷调查、  社康信息系统 |  |
| 1. **病情控制情况**   血压控制情况:  高血压并发症:  (新发心绞痛、新发心肌梗死、新发脑卒中、糖尿病、慢性肾脏疾病、高脂血症、其他)  是否有主要症状  （头晕、头痛、头胀、眩晕、心悸、胸闷、胸痛、视物模糊、肢体无力、肢体运动受阻、失眠、其他）  居民体检指标  血常规（血红蛋白、白细胞 、血小板）  尿常规（尿蛋白、尿糖、尿酮体、尿潜血），尿微量白蛋白，尿肌酐）  空腹血糖，糖化血红蛋白  肝功能（血清谷丙转氨酶、血清谷草转氨酶、总胆红素、结合胆红素 非结合胆红素）  肾功能（血清肌酐、血尿素、血尿酸）  血脂（总胆固醇、甘油三酯、血清低密度脂蛋白胆固醇、血清高密度脂蛋白胆固醇）  肿瘤筛查（癌胚抗原、甲胎蛋白），女性的宫颈涂片  心电图、胸部X线片、腹部B超、双肾、骨密度  转诊情况 | 社康信息系统的年度体检结果 |  |
| **七、居民对项目的满意度**  居民对体验馆的满意度包括：  对环境的满意度 （很满意、满意，一般，不满意）  对提供的检测设备满意度（很满意、满意，一般，不满意）  对提供的服务的满意度（很满意、满意，一般，不满意）  居民对课题组开展的主动健康相关活动的满意度  对组织形式的满意度（很满意、满意，一般，不满意）  对内容的的满意度（很满意、满意，一般，不满意）  对效果的满意度（很满意、满意，一般，不满意） 对高血压相关问题的健康指导满意度（很满意、满意，一般，不满意） | 问卷调查 |  |

**核心指标：**

经常阅读健康相关书籍、公众号、网页情况

常观看或收听健康讲座或视频

高血压基本知识掌握情况

降压药物知识掌握情况

定期自我监测血压频率

定期体检情况

配置了可穿戴设备患者的数据活度

睡眠（平均每天睡眠时间）

饮食口味习惯

饮酒的频率

抽烟状况（日吸烟量）

运动锻炼的频率

就医依从性

用药依从性情况

血压控制情况:

高血压并发症:

居民对高血压相关问题的健康指导满意度

**社区糖尿病患者主动健康监测和效果评估**

**说明：**社区糖尿病人群的检测指标主要是“居民主动健康素养”+国家基本公共卫生项目中关于糖尿病健康管理的指标，及其相关体检结果的动态变化结果。

|  | 数据获取方法 | 备注 |
| --- | --- | --- |
| **一、主动学习情况 界定主动被动**  经常阅读健康相关书籍、公众号、网页情况（每周4天以上）  （是、否）  观看或收听健康讲座或视频 （每周1次以上）  （是、否）  有健康相关问题，主动咨询医务人员  （是、否） | 主动健康素养问卷调查 |  |
| **二、糖尿病相关知识掌握情况**  糖尿病基本知识掌握情况  （完全掌握、部分掌握、不掌握）  基本的营养管理知识掌握情况  （完全掌握、部分掌握、不掌握）  基本的运动管理知识掌握情况  （完全掌握、部分掌握、不掌握）  降糖药物知识掌握情况  （完全掌握、部分掌握、不掌握） | 主动健康素养问卷调查 |  |
| **三、主动健康监测情况**  定期自我监测血糖  （每天监测、每周监测、每月监测、每半年监测、每年监测、很少测、从来没有测）  定期自我监测血压频率  （每天监测血压、每周监测血压、每月监测血压、每半年监测血压、每年监测血压、很少测血压、从来没有测血压）  定期体检情况  （每年体检、2年体检一次、三年以上体检一次）  定期自我监测体重  （每天称体重、每周称体重、每月称体重、每半年称体重、每年称体重、很少称体重）  定期做心理健康测评情况  （每天做心理健康测评、每周做心理健康测评、每月做心理健康测评、每半年做心理健康测评、每年做心理健康测评、很少做心理健康测评、从来没有做过心理健康测评）  配置了可穿戴设备患者的数据活度（如有） | 主动健康素养问卷调查  主动健康信息平台 |  |
| **四、生活行为情况**  睡眠（平均每天睡眠时间）  饮食口味习惯  （无特别、喜欢偏咸、喜欢偏淡、喜欢偏甜、喜欢偏辣、喜欢油炸）  主食摄入 （克/天）  饮酒的频率  （从来不喝酒、偶尔礼节性喝酒、每月喝酒、每周喝酒≥1次、每周喝酒≥3次、每天喝酒  日饮酒量（平均 两）  饮酒种类  （白酒、啤酒、红酒、黄酒、其他)  抽烟状况（日吸烟量）  运动（运动锻炼的频率、每次锻炼的时间、锻炼的方式）  平时参加社区、单位或学校组织的健康相关活动情况。  （从不参加、偶尔参加、经常积极参加） | 主动健康素养问卷调查、  社康信息系统  主动健康信息平台 |  |
| **五、主动健康干预情况**  就医依从性（当您与社康中心/医院有约定就诊时间，您是否都按时就诊？ 是、偶尔不按时、都不按时  用药依从性情况（当您要使用降糖药时）  （规律、间断、不服药、无需服药）  针对健康行为改变情况  （饮食行为的改善、运动行为的改善、心理状况的调整、其他调整，无作任何改变） | 主动健康素养问卷调查、  社康信息系统 |  |
| **六、病情控制情况**  血糖控制情况:空腹血糖，糖化血红蛋白  糖尿病相关症状:多饮、多食、多尿、视力模糊、感染、手脚麻木、下肢浮肿、体重明显下降、其他症状。  糖尿病主要并发症：糖尿病引发的感染性疾病、高血压、高血脂、糖尿病视网膜病变、糖尿病肾脏病变、远端神经病变、糖尿病足、眼睛相关病变、其他。  低血糖反应（ 有 无）  居民体检指标  血常规（血红蛋白、白细胞、血小板）  尿常规（尿蛋白、尿糖、尿酮体、尿潜血），尿微量白蛋白，尿肌酐）  肝功能（血清谷丙转氨酶、血清谷草转氨酶、总胆红素、结合胆红素 非结合胆红素）  肾功能（血清肌酐、血尿素、血尿酸）  血脂（总胆固醇、甘油三酯、血清低密度脂蛋白胆固醇、血清高密度脂蛋白胆固醇）  肿瘤筛查（癌胚抗原、甲胎蛋白），女性的宫颈涂片  心电图、胸部X线片、腹部B超、双肾、骨密度  转诊情况 | 社康信息系统的年度体检结果 |  |
| **七、居民对项目的满意度**  居民对体验馆的满意度包括：  对环境的满意度 （很满意、满意，一般，不满意）  对提供的检测设备满意度（很满意、满意，一般，不满意）  对提供的服务的满意度（很满意、满意，一般，不满意）  居民对课题组开展的主动健康相关活动的满意度  对组织形式的满意度（很满意、满意，一般，不满意）  对内容的的满意度（很满意、满意，一般，不满意）  对效果的满意度（很满意、满意，一般，不满意） 糖尿病相关问题的健康指导满意度（很满意、满意，一般，不满意） | 问卷调查 |  |

**核心指标：**

经常阅读健康相关书籍、公众号、网页情况

常观看或收听健康讲座或视频

糖尿病基本知识掌握情况

降糖药物知识掌握情况

定期自我监测血糖频率

定期体检情况

配置了可穿戴设备患者的数据活度

睡眠（平均每天睡眠时间）

饮食口味习惯

饮酒的频率

抽烟状况（日吸烟量）

运动锻炼的频率

就医依从性

用药依从性情况

血糖控制情况

糖尿病并发症

居民对糖尿病相关问题的健康指导满意度

**服药依从性测评表**

C1 您是否有忘记服药经历 ①是 ②否

C2 您是否有时不注意服药(没按药物说明书或医生处方少服、漏服) ①是 ②否

C3 当您自觉症状改善时,是否曾停药 ①是 ②否

C4 当您服药自觉症状更坏时 ,是否曾停药 ①是 ②否

**家庭关怀度指数问卷**

经常这样 有时这样 几乎很少

D1 当您遇到问题时，可以从家人得到满意的帮助 2 1 0

D2 您很满意家人与您讨论各种事情以及分担问题 2 1 0

的方式

D3 当您希望从事新的活动或发展时，家人都能接受 2 1 0

且给予支持

D4 您很满意家人对我的情绪（喜怒哀乐）表示关心和 2 1 0

爱护的方式

D5 您很满意家人与我共度时光的方式 2 1 0

**生活质量状况调查问卷（SF－36）**

1.总体来讲，您认为您的健康状况是： ①非常好 ②很好 ③好 ④一般 ⑤差

2.跟1年以前比您觉得自己的健康状况是：
 ①比1年前好多了 ②比1年前好一些 ③跟1年前差不多 ④比1年前差一些

⑤比1年前差多了健康和日常活动
3.您的健康状况是否限制了以下日常活动？如果有限制，程度如何？在相应描述后的框内打“√”

| 项 目 | 限制很大 | 有限制 | 毫无限制 |
| --- | --- | --- | --- |
| （1）重体力活动，如跑步、参加剧烈运动等 | □ | □ | □ |
| （2）适度的活动，如扫地、打太极拳等 | □ | □ | □ |
| （3）手提日用品，如买菜、购物等 | □ | □ | □ |
| （4）上几层楼梯 | □ | □ | □ |
| （5）上一层楼梯 | □ | □ | □ |
| （6）弯腰、屈膝、下蹲 | □ | □ | □ |
| （7）步行1600米以上的路程 | □ | □ | □ |
| （8）步行800米的路程 | □ | □ | □ |
| （9）步行100米的路程 | □ | □ | □ |
| （10）自己洗澡、穿衣 | □ | □ | □ |

4、在过去4个星期里，您的工作和日常活动有无因为身体健康的原因而出现以下这些问题？
（1）减少了工作或其他活动时间： ①是 ②不是

（2）本来想要做的事情只能完成一部分： ①是 ②不是
（3）想要干的工作或活动种类受到限制： ①是 ②不是
（4）完成工作或其他活动困难增多（比如需要额外的努力）： ①是 ②不是
5.在过去4个星期里，您的工作和日常活动有无因为情绪的原因（如压抑或忧虑）而出现以下这些问题？
（1）减少了工作或活动时间： ①是 ②不是

（2）本来想要做的事情只能完成一部分： ①是 ②不是
（3）干事情不如平时仔细： ①是 ②不是
6.在过去4个星期里，您的健康或情绪不好在多大程度上影响了您与家人、朋友、邻居或集体的正常社会交往？ ①完全没有影响 ②有一点影响 ③中等影响 ④影响很大 ⑤影响非常大
7.在过去4个星期里，您有身体疼痛吗？
 ①完全没有疼痛 ②有一点疼痛 ③中等疼痛 ④严重疼痛 ⑤很严重疼痛
8.在过去4个星期里，您的身体疼痛影响了您的工作和家务吗？
 ①完全没有影响 ②有一点影响 ③中等影响 ④影响很大 ⑤影响非常大
9.以下这些问题有关过去1个月里您自己的感觉，对每一条问题，您的情况是什么样？请在□里“√”出。

**总体健康情况**

**10.请看下列每一条问题，哪一种答案最符合您的情况？**

| **项 目** | **所有的时间** | **大部分时间** | **比较多时间** | **一部分时间** | **一小部分时间** | **没有这种感觉** | |
| --- | --- | --- | --- | --- | --- | --- | --- |
| （1）您觉得生活充实 | □ | □ | □ | □ | □ | □ | |
| （2）您是一个敏感的人 | □ | □ | □ | □ | □ | □ | |
| （3）您情绪非常不好，什么事情都不能使您高兴 | □ | □ | □ | □ | □ | | □ |
| （4）您心里很平静 | □ | □ | □ | □ | □ | □ | |
| （5）您做事情精力充沛 | □ | □ | □ | □ | □ | □ | |
| （6）您的情绪低落 | □ | □ | □ | □ | □ | □ | |
| （7）您觉得筋疲力尽 | □ | □ | □ | □ | □ | □ | |
| （8）您是个快乐的人 | □ | □ | □ | □ | □ | □ | |
| （9）您感到厌烦 | □ | □ | □ | □ | □ | □ | |
| （10）不健康影响了您的社会生活  （如走访亲戚） | □ | □ | □ | □ | □ | □ | |

**请在每一条问题后“√”出一个答案**

| **项 目** | 绝对  正确 | 大部分正确 | 不能  肯定 | 大部分错误 | 绝对  错误 |
| --- | --- | --- | --- | --- | --- |
| （1）我好像比别人容易生病 | □ | □ | □ | □ | □ |
| （2）我跟周围人一样健康 | □ | □ | □ | □ | □ |
| （3）我认为我的健康状况在变坏 | □ | □ | □ | □ | □ |
| （4）我的健康状况非常好 | □ | □ | □ | □ | □ |

**社会支持评定量表**

下面的问题用于反映您在社会中所获得的支持，请按各个问题的具体要求，根据您的实

际情况写，谢谢您的合作。

**E1、您有多少个关系密切，可以得到支持和帮助的朋友？（只选一项）**

①一个也没有 ②1－2 个 ③3－5 个 ④6 个或 6 个以上

**E2、近一年来您：（只选一项）**

①远离家人，且独居一室 ②住处经常变动，多数时间和陌生人住在一起

③和同学、同事或朋友住在一起 ④和家人住在一起

**E3、您和邻居：（只选一项）**

①相互之间从不关心，只是点头之交 ②遇到困难可能稍微关心

③有些邻居很关心您 ④大多数邻居都很关心您

**E4、您和同事：（只选一项）**

①相互之间从不关心，只是点头之交 ②遇到困难可能稍微关心

③有些同事很关心您 ④大多数同事都很关心您

**E5、从家庭成员得到的支持和照顾（在合适的框内划“√”）**

|  | 无 | 极少 | 一般 | 全力支持 |
| --- | --- | --- | --- | --- |
| a、夫妻（恋人） | 1 | 2 | 3 | 4 |
| b、父母 | 1 | 2 | 3 | 4 |
| c、儿女 | 1 | 2 | 3 | 4 |
| d、兄弟姐妹 | 1 | 2 | 3 | 4 |
| e、其它成员(如嫂子) | 1 | 2 | 3 | 4 |

**E6、过去，在您遇到急难情况时，曾经得到的经济支持和解决实际问题的帮助的来源有：**

（1）无任何来源**(跳至E7)**  （2）下列来源（可选多项）

①配偶 ②其他家人 ③亲戚 ④同事 ⑤工作单位

⑥党团工会等官方或半官方组织 ⑦宗教、社会团体等非官方组织

⑧其它（请列出）：

**E7、过去，在您遇到急难情况时，曾经得到的安慰和关心的来源有：**

（1）无任何来源**（跳至E8）** （2）下列来源（可选多项）

①配偶 ②其他家人 ③亲戚 ④同事 ⑤工作单位

⑥党团工会等官方或半官方组织 ⑦宗教、社会团体等非官方组织；

⑧其它（请列出）：

**E8、您遇到烦恼时的倾诉方式： （只选一项）**

①从不向任何人诉讼 ②只向关系极为密切的 1－2 个人诉讼

③如果朋友主动询问您会说出来 ④主动诉讼自己的烦恼，以获得支持和理解

**E9、您遇到烦恼时的求助方式：（只选一项）**

①只靠自己，不接受别人帮助 ②很少请求别人帮助

③有时请求别人帮助 ④有困难时经常向家人、亲友、组织求援

**E10、对于团体（如党组织、宗教组织、工会、学生会等）组织活动，您：（只选一项）**

①从不参加 ②偶尔参加 ③经常参加 ④主动参加并积极活动

**调查到此结束，感谢您的配合和参与！**
