# Supplementary material for: Digital Health Platform for Improving the Effect of the Active Health Management of Chronic Diseases in the Community: Mixed Methods Exploratory Study
Source: J Med Internet Res. 2024 Nov 18;26:e50959. doi: 10.2196/50959 (PMC11612601; doi:10.2196/50959)
Supplement: Multimedia Appendix 5 [file jmir_v26i1e50959_app5.docx]

**社区成年居民心脑血管疾病风险评估体系**

**说明：1.**信息系统医疗端：展示居民的心脑血管疾病风险总得分，用于分层管理。

**2.**小程序居民端：（1）展示主动健康得分（1000-行为风险得分-管理效果风险得分）;

（2）展示居民主动健康得分在本街道居民中的排名百分比。

**（一）基础病史风险**

| **序号** | **指标分类** | **风险指标** | **权重系数** | **变量赋值** |
| --- | --- | --- | --- | --- |
| 1 | **基础病史风险** | 年龄 | 3.8 | 0-10 |
| 2 |  | 性别 | 1.2 | 0-10 |
| 3 |  | 高血压病病程 | ①无高血压病病程者赋0分；  ②有高血压患者基础100分；  ③根据患病既往用药情况、血压控制情况、临床并发症、患病时间（年）累积得分，总分≤200分 。 | |
| 4 |  | 糖尿病病程 | ①无高糖尿病病程者赋0分；  ②有糖尿病患者基础100分；  ③根据患病既往用药情况、血糖控制情况、临床并发症、患病时间（年）累积得分，总分≤200分  ④如同时患有糖尿病和糖尿病者，两项得分相加×1.2。 | |
| 5 |  | 心脑血管事件病史 | ①无心脑血管病家族史者赋0分；  ②有心脑血管事件病史，但无患高血压糖尿病者，赋分150分。  ③有心脑血管事件病史，也是高血压或/和糖尿病者：高血压得分、糖尿病得分×0.5。 | |
| 6 |  | 心脑血管病家族史 | ①无心脑血管病家族史者赋0分；  ②有心脑血管病家族史的高血压和糖尿病者：高血压得分、糖尿病得分×0.05。  ③如无基础病，按照如下计算。 | |
|  |  |  | 2.0 | 0-10 |
| 7 |  | 高同型半胱氨酸血症 | ①同型半胱氨酸正常者赋0分；  ②高血压和糖尿病者同型半胱氨酸血症（轻度、中度、重度）者：高血压得分/糖尿病得分×0.03、0.06、0.1）。如无基础病，按照如下计算：、 | |
|  |  |  | 4 | 0-10 |

**（二）主动健康行为风险**

| 8 | 健康管理行为风险 | 吸烟 | 3.3 | 0-10 |
| --- | --- | --- | --- | --- |
| 9 |  | 饮酒 | 3.2 | 0-10 |
| 10 |  | 运动 | 3.2 | 0-10 |
| 11 |  | 饮食 | 3.3 | 0-10 |
| 12 |  | 睡眠 | 2 | 0-10 |
| 13 |  | 用药 | 4 | 0-10 |
| 14 |  | 体检 | 1 | 0-10 |

**（三）主动健康管理效果风险**

| 15 | 健康管理效果风险 | 当前血压水平 | 3 | 0-10 |
| --- | --- | --- | --- | --- |
| 16 |  | 当前血糖水平 | 3 | 0-10 |
| 17 |  | 体质指数BMI/腰围 | 2 | 0-10 |
| 18 |  | 甘油三脂 | 2.8 | 0-10 |
| 19 |  | 高密度脂蛋白胆固醇 | 2 | 0-10 |
| 20 |  | 低密度脂蛋白胆固醇 | 3.8 | 0-10 |
| 21 |  | 高尿酸血症 | 1.5 | 0-10 |
| 22 |  | 微量白蛋白尿 | 1.5 | 0-10 |
| 23 |  | 颈动脉斑块 | 4.1 | 0-10 |
| 24 |  | 心理疾患 | 1.3 | 0-10 |

**二、问卷**

**社区成年居民心脑血管疾病风险评估问卷**

1.年龄（岁）

2.性别 男 女

3.患高血压病情况 无 有（初诊时血压情况:收缩压： /舒张压 mmHg）

确诊高血压病时间: 年

4.确诊时初次血糖情况（mmol/L）

确诊糖尿病时间: 年

1. 您既往是否有心脑血管事件病史： 无 有

6.心脑血管病家族史 无 有

7.血清同型半胱氨酸(μmol/L)

8.在近2周，你的是否有如下用药行为：

无须用药

严格按照医生要求服药

您有时忘记服药? 您有时不注意服药?

当您自觉症状改善时，曾停止服药? 当您服药后自觉症状更糟时，曾停止服药?

9.最近一周平均每日吸烟量（支）

10.最近一周饮酒次数： 每次饮酒量（ml）：

饮酒种类：白酒 红酒 黄酒 啤酒 其他

11.运动：每周运动锻炼 天，每次 分钟，

运动的种类：跑步、 快步走、爬山、球类、太极、八段锦，其他

12.饮食习惯

膳食均衡、按时就餐

膳食均衡、常有就餐不定时

常有就餐不定时，而且有嗜甜/盐/油之一行为

1. 平均每天睡眠时间（小时）
2. 你最近一次各人体检离现在有多久

一年内 两年内 超过2年

15.当前血压水平 收缩压/ 舒张压 mmHg

16.当前空腹血糖水平（mmol/L）

17.体质指数BMI：

腰围（测量肚脐以上1cm)

18.甘油三脂（mmol/L）

19.高密度脂蛋白胆固醇（mmol/L）

20.低密度脂蛋白胆固醇（mmol/L）

21.血尿酸(μmol/L)

22.尿微量白蛋白(mg/L)

23.颈动脉斑块 无 有

24.最近一周的心理健康问题 无 有（有下列症状之一：情绪低落、情绪冲动、紧张状态、抑郁、焦虑、幻听、妄想、强迫症等。）

25.目前出现高血压糖尿病相关并发症;

无 有（冠心病、肾功能损伤、眼底改变、糖尿病性周围神经病、糖尿病性自主神经病变、糖尿病足病、微血管病变、房颤）

三、社区成年居民心血管风险疾病评估变量赋分情况

1.年龄

数据来源：居民健康档案个人基本信息、问卷调查

| 年龄（岁） | 变量赋分 |
| --- | --- |
| ＜35 | 0.0 |
| 35-39 | 1.6 |
| 40-44 | 1.7 |
| 45-49 | 1.8 |
| 50-54 | 2.0 |
| 55-59 | 2.4 |
| 60-64 | 3.2 |
| 65-69 | 3.8 |
| 70-74 | 5.4 |
| 75-79 | 7.4 |
| 80-84 | 9.3 |
| ≥85 | 10.0 |

2.性别

数据来源：居民健康档案个人基本信息、问卷调查

| 性别 | 变量赋分 |
| --- | --- |
| 女性（＜50 岁） | 0 |
| 女性（≥50 岁） | 10 |
| 男性 | 10 |

**3.高血压病病程**

数据来源：高血压病管理专项档案数据，健康监测数据

| 高血压病病程 | 变量赋分 |
| --- | --- |
| （1）无 | 0 |
| （2）高血压病患者 | 100 |
| （3）用药情况 规律用药  不规律用药  不用药 | 加0分  加10分  加20分 |
| （4）血压控制情况 血压控制良好  血压控制不良 | 加0分  加20分 |
| （5）高血压并发症 无并发症  有1个并发症  有2个并发症  有3个以上并发症 | 加0分  加10分  加20分  加30分 |
| （6）患高血压病时间（年） | 患病时间每1年加2分  **高血压病病程累计总分不超过200分** |

4.糖尿病病程

数据来源：糖尿病管理专项档案数据，健康监测数据

| 糖尿病病程 | 变量赋分 |
| --- | --- |
| （1）无 | 0 |
| （2）糖尿病患者 | 100 |
| （3）用药情况 规律用药  不规律用药  不用药 | 加0分  加10分  加20分 |
| （4）血糖控制情况 血糖控制良好  血糖控制不良 | 加0分  加20分 |
| （5）糖尿病并发症 无并发症  有1个并发症  有2个并发症  有3个以上并发症 | 加0分  加10分  加20分  加30分 |
| （6）患糖尿病时间（年） | 患病时间每1年加2分  **糖尿病病程累计总分不超过200分** |
| **如同时患有糖尿病和糖尿病者，两项得分相加后×1.2。** | |

5.心脑血管事件病史

数据来源：居民健康档案个人基本信息、患者自填问卷

| 心脑血管事件病史 | 变量赋分 |
| --- | --- |
| 无心脑血管事件病史 | 0 |
| 有心脑血管事件病史 | 高血压得分、糖尿病得分×1.5。 |
|  | 如无患高血压糖尿病者，赋分150分。 |

6.心脑血管病家族史

数据来源：居民健康档案个人基本信息、患者自填问卷

高血压和糖尿病者：高血压得分、糖尿病得分×1.05。如无高血压糖尿病者，按如下计算：

| 心脑血管病家族史 | 变量赋分 |
| --- | --- |
| 无心脑血管病家族史 | 0 |
| 父母、兄弟姐妹中有脑卒中或心血管事件病史 | 10 |

7.高同型半胱氨酸血症

数据来源：居民健康档案、最近一次检测记录

高血压和糖尿病者同型半胱氨酸血症（正常、轻度、中度、重度）：高血压得分、糖尿病得分分别×1.0、1.03、1.06、1.1）。如无基础病，按照如下计算：

| 同型半胱氨酸（µmol/L） | 变量分值赋分 |
| --- | --- |
| 正常（同型半胱氨酸＜≤10） | 0 |
| 轻度（10<同型半胱氨酸≤15） | 3 |
| 中度（15<同型半胱氨酸≤30 ） | 6 |
| 重度（同型半胱氨酸>30） | 10 |

8.吸烟

数据来源：最近一次健康随访记录、患者自填问卷

| 吸烟情况 | 变量赋分 |
| --- | --- |
| 不吸烟 | 0 |
| 最近一周的平均每日吸烟量1-5支 | 2.5 |
| 最近一周的平均每日吸烟量5-10支 | 5 |
| 最近一周的平均每日吸烟量10-20支 | 7.5 |
| 最近一周的平均每日吸烟量大于20支 | 10 |

9.饮酒

数据来源：最近一次健康随访记录、患者自填问卷

| 饮酒情况 | 变量赋分 |
| --- | --- |
| 不饮酒 | 0 |
| 一周饮酒1次，或  平均日饮不超：白酒量50ml、红酒150ml、啤酒750ml. | 2.5 |
| 一周饮酒2次，或  男性平均日饮白酒量50-100ml、红酒150-300ml、啤酒750ml-1500ml. | 5 |
| 一周饮酒3次，或  男性平均日饮白酒量100-150ml、红酒300-450ml、啤酒1500ml-2250ml. | 7.5 |
| 一周饮酒3次以上，或  平均日饮超过白酒量150ml、红酒450ml、啤酒2250ml. | 10 |

**不同种类酒的换算方法：白酒：红酒：啤酒=1：3：15**


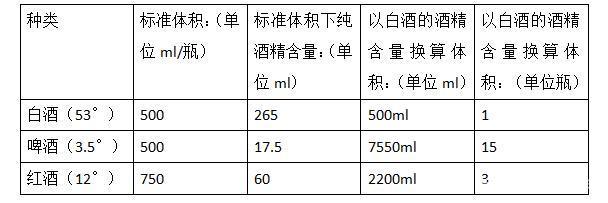


**如按照酒精计算：**

（1）白酒，每瓶53度500ml的白酒，酒精含量为53%，即265ml纯酒精，1ml53度的白酒含0.53ml纯酒精。

（2）红酒的酒精含量一般12度左右，大多采用的是750毫升瓶装，500ml的红酒，酒精含量为60ml，1ml红酒含0.12ml纯酒精。

（3）啤酒的酒精度约3.5%，500ml啤酒含17.5ml酒精，1ml啤酒含0.035ml纯酒精。

10.缺乏运动

数据来源：最近一次健康随访记录、患者自填问卷

| **运动情况** | **变量赋分** |
| --- | --- |
| 经常锻炼（每周锻炼3天及以上，每次30分钟以上） | 0 |
| 偶尔锻炼（每周锻炼1-2天，每次30分钟以上） | 5 |
| 不锻炼（每周锻炼小于1天） | 10 |

**运动消耗热量换算方法，各种运动每半小时消耗的热量如下：**

| 1.快跑≈350kcal  2.慢跑≈328kcal  3.快走≈276kcal  4.慢走≈128kcal  5.原地慢跑≈240kcal  6.游泳量≈260kcal  7.打太极拳≈120kcal  8.跳绳≈400kcal  9.爬山≈250kcal | 10.仰卧起坐≈216kcal  11.篮球≈250kcal  12.打网球≈212kcal  13.排球≈175kcal  14.桌球≈150kcal  15.交谊舞≈150kcal  16.骑单车≈207kcal  17.有氧健身操≈180kcal  18.登楼梯≈175kcal | 19.转呼啦圈≈150kcal  20.散步≈97kcal  21.站立≈60kcal  22.坐姿≈30kcal  23.睡眠≈27kcal  24.滑雪≈300kcal  25.溜冰≈175kcal |
| --- | --- | --- |

11.饮食习惯

数据来源：最近一次健康随访记录、患者自填问卷

| 饮食习惯 | **变量赋分** |
| --- | --- |
| 膳食均衡、按时就餐 | 0 |
| 膳食均衡、常有就餐不定时 | 5 |
| 常有就餐不定时，而且有嗜甜/盐/油之一行为 | 10 |

12.睡眠情况

数据来源：最近一次健康随访记录、患者自填问卷

| 睡眠情况 | **变量赋分** |
| --- | --- |
| 平均每天睡眠时间7-8小时 | 0 |
| 平均每天睡眠时间6-7小时 | 2.5 |
| 平均每天睡眠时间5-6小时 | 5 |
| 平均每天睡眠时间4-5小时 | 7.5 |
| 平均每天睡眠时间小于4小时 | 10 |

13.用药行为

数据来源：最近一次健康随访记录、患者自填问卷

| 用药行为 | **变量赋分** |
| --- | --- |
| 无须用药，或严格按照医生要求服药 | 0 |
| 有1项不依从用药行为 | 4 |
| 有2项不依从用药行为 | 6 |
| 有3项不依从用药行为 | 8 |
| 有4项不依从用药行为 | 10 |

14.体检

数据来源：最近一次健康随访记录、患者自填问卷

| **参加定期体检** | **变量赋分** |
| --- | --- |
| 最近1年有体检 | 0 |
| 最近2年有体检 | 5 |
| 近2年未参加过体检 | 10 |

15.当前血压水平

数据来源：居民健康档案—最近一次体检记录、健康监测或临床诊疗记录


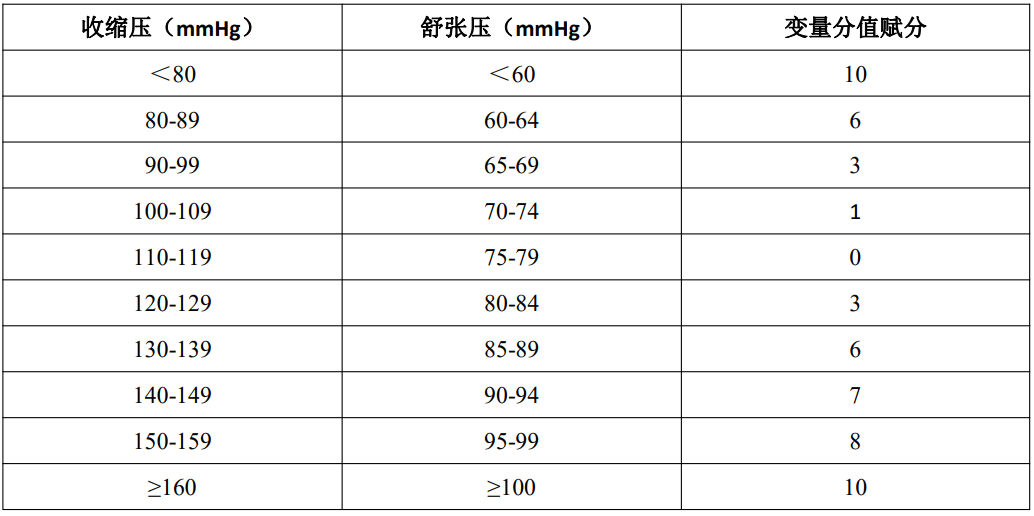


收缩压/舒张压取赋分高值。

16.当前血糖水平

数据来源：居民健康档案—最近一次体检记录、健康监测或临床诊疗记录


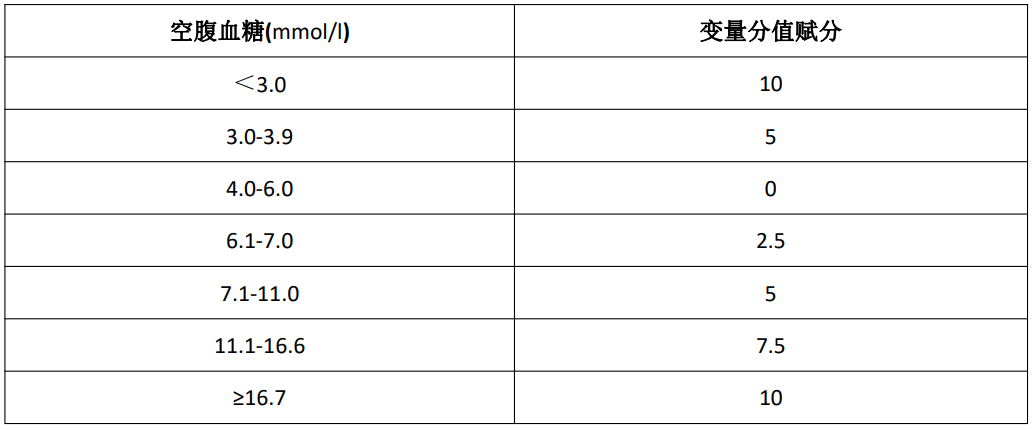


17.体质指数（BMI）

数据来源：居民健康档案、最近一次体检记录或健康随访记录

| 体质指数（BMI） | 变量赋分 |
| --- | --- |
| ＜18.5 | 7.6 |
| 18.5-23.9 | 0 |
| 24-27.9 | 3.2 |
| ≥28 | 10 |

备注：分段设置按照《中国成人超重和肥胖症预防控制指南》中体重过低、正常体重、超重、肥胖的标准进行调整。

**或，**腰围

数据来源：居民健康档案、最近一次体检记录或健康随访记录


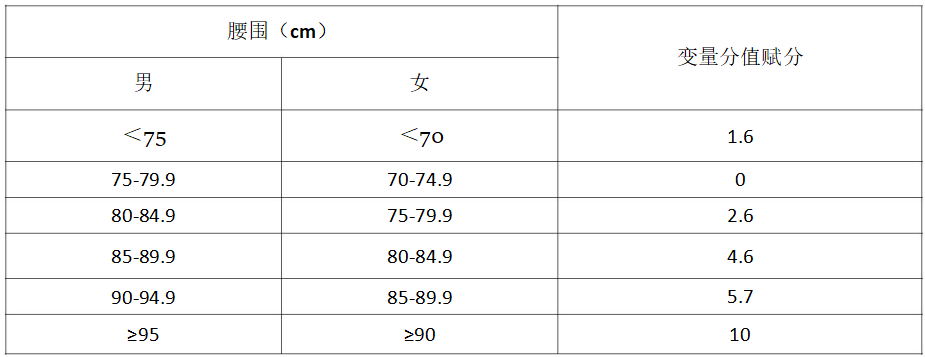


备注：体质指数BMI与腰围两者中取最大值，不重复计分。

18.甘油三脂

数据来源：居民健康档案、最近一次体检记录

| 甘油三脂（mmol/L） | 变量赋分 |
| --- | --- |
| ＜2.26 | 0 |
| ≥2.26 | 10 |

19.高密度脂蛋白胆固醇

数据来源：居民健康档案个人基本信息、最近一次体检记录

| 高密度脂蛋白胆固醇  （mmol/L） | 变量赋分 |
| --- | --- |
| ≥1.04 | 0 |
| ＜1.04 | 10 |

20.低密度脂蛋白胆固醇

数据来源：居民健康档案个人基本信息、最近一次体检记录

| 低密度脂蛋白胆固醇（mmol/L） | 变量赋分 |
| --- | --- |
| ＜4.14 | 0 |
| ≥4.14 | 10 |

21.颈动脉斑块

数据来源：居民健康档案、最近一次检测记录

| 颈动脉斑块 | 变量赋分 |
| --- | --- |
| 无 | 0 |
| 有 | 10 |

22.高尿酸血症

数据来源：居民健康档案、最近一次检测记录


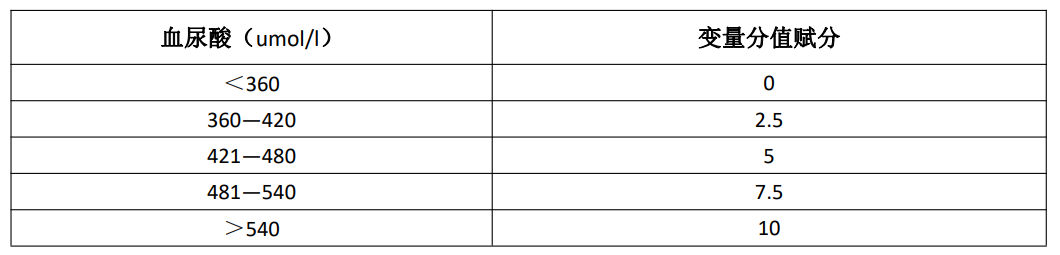


23.微量白蛋白尿

数据来源：居民健康档案、最近一次检测记录


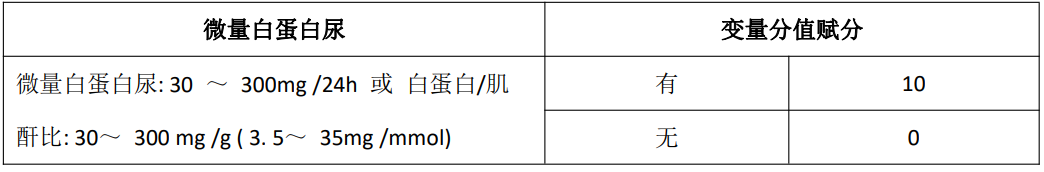


24.心理问题

数据来源：最后的随访记录和临床诊疗记录

| 心理健康问题 | 变量赋分 |
| --- | --- |
| 最近一周无心理健康问题 | 0 |
| 最近一周有出现下列心理健康问题者：情绪低落、情绪冲动、紧张状态、抑郁、焦虑、幻听、妄想、强迫症等。 | 10 |
